# 2023 Beijing Health Data Science Summit

**DOI:** 10.34133/hds.0112

**Published:** 2024-06-07

## Abstract

The 5th annual Beijing Health Data Science Summit, organized by the National Institute of Health Data Science at Peking University, recently concluded with resounding success. This year, the summit aimed to foster collaboration among researchers, practitioners, and stakeholders in the field of health data science to advance the use of data for better health outcomes.

One significant highlight of this year’s summit was the introduction of the Abstract Competition, organized by *Health Data Science*, a Science Partner Journal, which focused on the use of cutting-edge data science methodologies, particularly the application of artificial intelligence in the healthcare scenarios. The competition provided a platform for researchers to showcase their groundbreaking work and innovations.

In total, the summit received 61 abstract submissions. Following a rigorous evaluation process by the Abstract Review Committee, eight exceptional abstracts were selected to compete in the final round and give presentations in the Abstract Competition.

The winners of the Abstract Competition are as follows:

• First Prize: “Interpretable Machine Learning for Predicting Outcomes of Childhood Kawasaki Disease: Electronic Health Record Analysis” presented by researchers from the Chinese Academy of Medical Sciences, Peking Union Medical College, and Chongqing Medical University (presenter Yifan Duan).
• Second Prize: “Survival Disparities among Mobility Patterns of Patients with Cancer: A Population-Based Study” presented by a team from Peking University (presenter Fengyu Wen).
• Third Prize: “Deep Learning-Based Real-Time Predictive Model for the Development of Acute Stroke” presented by researchers from Beijing Tiantan Hospital (presenter Lan Lan).

We extend our heartfelt gratitude to the esteemed panel of judges whose expertise and dedication ensured the fairness and quality of the competition. The judging panel included Jiebo Luo from the University of Rochester (chair), Shenda Hong from Peking University, Xiaozhong Liu from Worcester Polytechnic Institute, Liu Yang from Hong Kong Baptist University, Ma Jianzhu from Tsinghua University, Ting Ma from Harbin Institute of Technology, and Jian Tang from Mila–Quebec Artificial Intelligence Institute. We wish to convey our deep appreciation to Zixuan He and Haoyang Hong for their invaluable assistance in the meticulous planning and execution of the event.

As the 2023 Beijing Health Data Science Summit comes to a close, we look forward to welcoming all participants to join us in 2024. Together, we will continue to advance the frontiers of health data science and work toward a healthier future for all.

**Background:** Wealthy clinical experience and professional medical knowledge of doctors are contained in a large amount of electronic health records (EHRs). However, EHRs often exist in the form of free text. Various text processing operations such as annotation, data structuring, and standardization are necessarily required for data analysis and knowledge representation of EHRs, which costs a lot of resources. With the development of deep learning technology in natural language processing domain, pre-trained language model techniques like BERT have been greatly developed. By utilizing pre-trained language model, EHR documents can be represented into high-dimensional embedding space, which facilitates knowledge representation and feature extraction for EHR document processing. Deep learning approaches combined with pre-trained language models can be an efficient way to help machines to learn clinical experience and professional medical knowledge of doctors contained in EHRs.

**Objectives:** EHRs contain a large amount of clinical experience and professional medical knowledge. In this study, we aim to achieve knowledge representation of EHRs via a pre-trained language model and construct a deep learning approach to predict patient disease diagnosis based on EHR data.

**Methods:** The EHR data used in this research come from the “Huawei Cloud Cup” 2022 Artificial Intelligence Innovation Application Competition. The dataset includes 11,068 EHRs, each of which is composed of patient number, age, gender, chief complaint, current medical history, past medical history, physical examination, auxiliary examination, and discharge diagnosis. A total of 52 kinds of disease are involved in the dataset. The ratio of training set, validation set, and testing set is 6:2:2.

We applied pre-trained language models (BERT and MacBERT) to encode EHRs into numerical vectors for knowledge representation. Different encoding methods were tested to find the optimal strategy to represent the knowledge of diagnosis in the EHRs. A deep learning-based classification model for diagnosis prediction was then trained. Encoded EHR vectors were fed into the classification model, and diagnosis results were predicted by the model.

**Results:** Compared to the best performance proposed in the “Huawei Cloud Cup” 2022 Competition, our optimal method (MacBERT-base-Chinese-512-concatenate) achieves a significant improvement from F1 score = 0.6695 to F1 score = 0.6905.

In addition, the results in Table 1 show that the larger the encoding length, the better the diagnosis prediction performance. Models with max-length = 512 encoding parameter have significantly better performance than models with max-length = 256 encoding parameter. Models with multiple segments encoded have better performance than single segment encoded models. However, different feature integration methods (concatenate and average) do not significantly affect the prediction performance.

**Table 1.** Results of diagnosis prediction performance. Numbers with bold font indicate the best performance.

Models	Precision	Recall	F1 score
BERT-base-Chinese-256-single	0.6001	0.6148	0.6077
BERT-base-Chinese-256-concatenate	0.6081	0.67	0.6376
BERT-base-Chinese-256-average	0.6215	0.6626	0.6414
BERT-base-Chinese-512-concatenate	0.6739	0.7056	0.6893
BERT-base-Chinese-512-average	0.6562	0.7062	0.6803
MacBERT-base-Chinese-256-single	0.6017	0.6285	0.6148
MacBERT-base-Chinese-256-concatenate	0.6119	0.6678	0.6387
MacBERT-base-Chinese-256-average	0.6017	0.679	0.6381
MacBERT-base-Chinese-512-concatenate	**0.667**	**0.7157**	**0.6905**
MacBERT-base-Chinese-512-average	0.6562	0.7267	0.6897

**Conclusion:** We propose a deep learning approach to predict diagnosis using EHRs in this study. Our optimal method achieves a new state-of-the-art prediction performance on “Huawei Cloud Cup” 2022 Competition dataset. This study proves the feasibility of pre-trained language models on knowledge representation of EHRs in Chinese, which may facilitate and inspire AI applications for clinical practice in the future.
