# Supplementary material for: 2023 Beijing Health Data Science Summit
Source: Health Data Sci. 2024 Jun 7;4:0112. doi: 10.34133/hds.0112 (PMC11157085; doi:10.34133/hds.0112)
Supplement: Related texts

# Abstract: 2. A Multi-Criteria Decision Analysis Map for Disease-Level Research Resources Allocation

**Authors:** Wenjing Zhao, Shuang Wang, Jian Du

**Affiliations:** ^1^ National Institute of Health Data Science, Peking University, Beijing, China.; ^2^ Institute of Medical Technology, Peking University, Beijing, China.; * Address correspondence to: dujian@bjmu.edu.cn; † These authors contributed equally to this work.

**Background:** The imbalance^1^,^2^ and unpredictability^3^,^4^ of scientific discoveries pose significant challenges for optimal decision-making about research resources allocation. Science policy has been gradually moving over the past few decades in the direction of addressing social issues. For instance, in biomedical and health fields, health needs, measured by the burden of diseases, are recommended to be considered as one of the factors for allocating fundings at the diseases level.^5^,^6^ However, empirical data always showed an imbalance between the level of burden and research, with over- and under-investigated areas. Only referring to the burden of disease may produce bias since there is heterogeneity in the scientific complexity and difficulty for different diseases. The prevalence level of knowledgeable ignorance (e.g., the absence of fact, understanding, insight, or clarity about a specific disease, which leads us to frame better questions) and significant advances (e.g., providing new treatment opportunities) should also be considered. At the disease level, the burden of disease constitutes the pulling force of science, whereas knowledgeable ignorance and significant advances are the driving force of science.

**Objectives:** The goal of this work is to build a multi-criteria decision analysis map for disease-level resources allocation by integrating epidemiological and large-scale literature data from the three viewpoints: disease burden, knowledgeable ignorance, and significant advances.

**Methods:** In terms of disease burden, the World Bank proposed disability adjusted life years (DALYs) as a composite index in 1993. This measurement is reached by calculating the time lost through premature death and the time lived in a state of less-than-optimal health, loosely referred to as “disability.” The DALYs data used in this study are released by global health estimates (GHEs) of the World Health Organization (WHO) in 2016.

In terms of knowledgeable ignorance, the statements referring to ignorance are identified based on Subject-Predicate-Object structure extracted from literature data in PubMed. In particular, the National Institute of Health Data Science, Peking University’s Structured Medical Knowledge System Platform (cbk.bjmu.edu.cn) was used to retrieve phrases containing the chosen trigger words (“unknown; suspect; unclear; unusual; controversial; consensus; incomplete; conflicting; contrary; debatable; inconsistent; uncertain; unexpected; confusing; paradoxical”) in order to produce “ignorance” statements. Such sentences reflect unsolved scientific problems by describing unknown mechanism or cause, conflicting and inconsistent results, incomplete relationships, and so forth.

In terms of significant advances, Smith indicated post-publication review as the process whereby academia decides whether research matters.^4^ Fortunately, science provides a process for self-purification through scientific comments. A special publishing category, comment, in bibliographic databases like PubMed designates such work as a critique or explanation of a previously published study. Commented papers are, on average, much more cited than non-commented papers. Commented publications from PubMed were utilized in this study as a quantitative representation of significant advances.

In this study, WHO-established GHE cause category according to ICD-10 is used for disease categorization and mapping. By utilizing the MeSH labels provided by PubMed and the primary concordance table created in previous study between ICD-10 and MeSH,^7^ we were able to construct and extend the mapping relationship between MeSH and GHE causes and realize the classification of disease for knowledgeable ignorance and significant advances.

**Results:** Figure 1 provides a landscape of disease burden, ratio of knowledgeable ignorance, and significant advances for 20 categories of diseases, which can be further divided into more detailed subcategories according to the GHE hierarchical level. For instance, the top 2 high-burden NCDs are the diseases with highest knowledgeable ignorance and significant advances, indicating the consistency between health needs with research efforts, and revealing that it is necessary to further strengthen the sustained investment in the research of two diseases.

**Fig. 1.** The multi-criteria decision analysis map for disease-level research resource allocation.

**Conclusion:** Our evidence map supports multi-criteria decision making as compared to only using mortality and prevalence as comparable indicators for the funding allocation by RCDC in NIH.^8^ Statistical models [e.g., subjective expected utility (SEU)] will be designed to assist resource allocation by leveraging the weightiness of each criterion in future study.

## References

[1] Funk RJ, Owen-Smith J. A dynamic network measure of technological change. Manag Sci. 2017;63(3):791–817.

[2] Park M, Leahey E, Funk RJ. Papers and patents are becoming less disruptive over time. Nature. 2023;613(7942):138–144.36600070 10.1038/s41586-022-05543-x

[3] Yaqub O. Serendipity: Towards a taxonomy and a theory*. Res Policy.* 2018;47(1):169–179*.*

[4] Clauset A, Larremore DB, Sinatra R. Data-driven predictions in the science of science. Science. 2017;355(6324):477–480.28154048 10.1126/science.aal4217

[5] Gross CP, Anderson GF, Powe NR. The relation between funding by the National Institutes of Health and the burden of disease. N Engl J Med. 1999;340(24):1881–1887.10369852 10.1056/NEJM199906173402406

[6] Institute of Medicine (US) Committee on the NIH Research Priority-Setting Process. *Scientific opportunities and public needs: Improving priority setting and public input at the National Institutes of Health*. Washington (DC): National Academies Press (US);1998.20845560

[7] Yegros-Yegros A, van de Klippe W, Abad-Garcia MF, Rafols I. Exploring why global health needs are unmet by research efforts: The potential influences of geography, industry and publication incentives. Health Res Policy Syst. 2020;18(1):1–1432414373 10.1186/s12961-020-00560-6PMC7227286

[8] https://report.nih.gov/funding/categorical-spending#/

# Abstract: 3. A Two-Stage Model for Dietary Nutrient Recommendation and Mortality Risk Evaluation in PD Patients

**Authors:** Yueying Wu, Liantao Ma, Yasha Wang, Wen Tang

**Affiliations:** ^1^ Peking University, Beijing, China.; ^2^ Department of Nephrology, Peking University Third Hospital, Beijing, China.; * Address correspondence to: wangyasha@pku.edu.cn (Y.W.); tanggwen@126.com (W.T.); † These authors contributed equally to this work.

**Background:** Effective diet and nutritional management is crucial for patients with end-stage renal disease (ESRD) as excess intake can burden the kidneys, while inadequate intake may increase the risk of malnutrition and mortality. Accurate diet management is particularly important for ESRD patients receiving peritoneal dialysis (PD). Given the differences in the physical conditions of patients with chronic disease, precise dietary plans based on individual patients’ needs is essential for better outcomes.

Current research methods have only attempted to explore the relationship between diet and patient survival risk or provide rough dietary recommendations based on single baseline dietary data and examination data, using traditional Cox regression methods or machine learning methods. However, there has yet to be a focus on long-term follow-up data and dietary records at the nutritional level for survival analysis or personalized dietary recommendations.

Overall, while some of the dietary data analysis research approaches and findings provide informative insights, their level of personalization, nutritional granularity, follow-up density, accuracy of conclusions, and ease of use fall short in meeting the dietary and nutritional recommendations needed for PD patients.

**Objectives:** This study proposes a model that integrates long-term follow-up data and diverse dietary nutrient information to investigate the correlation between different types of nutrient intake and the risk of mortality among patients with PD. Based on the analysis of nutrition-related mortality risk, the study ultimately aims to identify precise and appropriate dietary intake thresholds that can effectively reduce the risk of mortality for PD patients.

**Methods:** In this study, we propose a two-stage model consisting of mortality risk prediction and dietary nutrient threshold determination to evaluate the impact of diet on patient mortality risk and provide personalized dietary recommendations based on patient subtypes (Fig. 2).

**Fig. 2.** Dietary nutrition recommendation research overview for PD patients.

In the first stage, we utilized the Cox method for mortality risk prediction and examined the statistical reliability of the results. In the second stage, we employed the restricted cubic spline method for mortality risk fitting to identify appropriate dietary nutrient intake ranges for recommendation and calculate 95% confidence intervals. To test for nonlinear relationships between dietary nutrients and mortality risk (two-tailed test, *P* < 0.05), we used analysis of variance (ANOVA). We provided precise upper and lower dietary nutrient recommendations for nonlinear mortality risk relationships, while upper or lower limit recommendations were provided for linear relationships.

Our model incorporates long-term dietary and follow-up data, with initial visit data used as an adjustment variable. Specifically, we collected clinical follow-up and demographic data from 13,091 visits of 656 patients with PD at Peking University Third Hospital over a period of 12 years. Each patient had approximately 20 visits, including 17 indicators, with an average interval of 2.7 months and an average follow-up time of 4 years. On average, each patient had 10 dietary records, including 26 indicators, with one record every 4 months. To handle uneven time intervals and ensure data diversity, we calculated time-averaged values for each patient’s dietary and follow-up data.

In summary, our study integrates long-term follow-up data and fine-grained dietary nutrient information and proposes a two-stage dietary nutrient recommendation model that simultaneously evaluates mortality risk. We validated the effectiveness of the recommended results based on retrospective cohort data by comparing the concordance between real-world intake and recommendation.

**Results and Conclusion:**

*Population contribution:* We provided personalized dietary recommendations for PD patients based on their albumin subtypes, a novel approach in the field. Despite the clinical attention given to albumin as a marker of health and nutrition, there are still no comprehensive studies on dietary nutrients based on different albumin level.

*Nutrient scope:* We comprehensively explored the relationship between 26 dietary nutrient elements and PD patient survival risk, including macronutrients such as protein, calories, daily protein intake (DPI), and sodium, which have been frequently studied, as well as micronutrients such as copper, magnesium, selenium, riboflavin, and nicotinic acid, which have received less attention.

*Result format:* For the first time, we have established that there exists a nonlinear relationship between dietary nutrient intake and survival risk. Based on this nonlinear relationship, we have obtained precise threshold ranges for the intake of various nutrients in the diet.

*Result details:* Our research reveals that calorie intake and mortality risk have a nonlinear relationship when patients’ types are not differentiated. We propose a daily intake range of 1,524.73 to 2,383.3 kcal/d for calories. Specifically, we recommend a calorie intake of 1,501 to 1,992 kcal/d for low-albumin patients, 1,481 to 1,972 kcal/d for medium-albumin patients, and a calorie intake exceeding 1,560 kcal/d for high-albumin patients. Our study also shows a nonlinear relationship between protein intake and mortality risk in all patient types. We recommend a protein intake range of 50.29 to 74.76 g/d, with a high-protein group recommended to consume 51.29 g/d and a low protein group recommended to consume 52 to 62 g/d.

Clinical practitioners can verify the validity of our methods and the rationality of our conclusions. Our conclusions can be substantiated by a vast body of existing research while also supplementing and enhancing existing findings.

# Abstract: 4. Air Pollution and Risk of Incident Chronic Kidney Disease among Diabetic Patients: An Exposure–Response Analysis

**Authors:** Feifei Zhang, Chao Yang, Fulin Wang, Yuhao Liu, Pengfei Li, Luxia Zhang

**Affiliations:** ^1^ National Institute of Health Data Science at Peking University, Health Science Center of Peking University, Beijing 100191, China.; ^2^ Institute of Medical Technology, Health Science Center of Peking University, Beijing 100191, China.; ^3^ Renal Division, Department of Medicine, Peking University First Hospital, Peking University Institute of Nephrology, Beijing 100034, China.; ^4^ Research Units of Diagnosis and Treatment of Immune-Mediated Kidney Diseases, Chinese Academy of Medical Sciences, Beijing 100034, China.; ^5^ Advanced Institute of Information Technology, Peking University, Hangzhou 311215, China.; ^6^ Health Science Center of Peking University, Beijing 100191, China.; * Address correspondence to: zhanglx@bjmu.edu.cn

**Background:** Impact of air pollution on the onset of chronic kidney disease (CKD) in diabetic patients [diabetic kidney disease (DKD)] is insufficiently studied.

**Objectives:** We aimed to examine exposure–response associations of PM_2.5_, PM_10_, PM_2.5–10_, NO_2_, and NO*_X_* with DKD at low air pollution levels in the UK. We also widened exposure level of PM_2.5_ and examined PM_2.5_–DKD association over a full range of global PM_2.5_ concentrations.

**Methods:** We applied Cox proportional hazards models and the shape constrained health impact function (SCHIF) to investigate the associations between air pollutants and incident DKD in the UK. Global exposure mortality model (GEMM) was applied to combine the PM_2.5_–DKD association in the UK with all other published associations.

**Results:** Multiple air pollutants were positively associated with DKD in the UK, with hazard ratios (HRs) of 1.632 [95% confidence interval (CI): 1.224 to 2.175], 1.074 (95% CI: 1.031 to 1.119), and 1.023 (95% CI: 1.004 to 1.042) for every 10 μg/m^3^ increase in PM_2.5_, NO_2_, and NO*_X_*, respectively. Exposure–response function in the UK suggested supralinear or sublinear associations over lower concentrations and then near-linear associations over higher concentrations of PM_2.5_, NO_2_, and NO*_X_* (Figure 3). When combining with all other published PM_2.5_–DKD associations in the globe, the exposure–response plot exhibited an exponential increasing trend in the risk of DKD as PM_2.5_ concentration increased.

**Fig. 3.** Exposure–response associations between air pollutants and DKD in the UK. (A) PM_2.5_. (B) PM_10_. (C) PM_2.5–10_. (D) NO_2_. (E) NO*_X_*.

**Conclusion:** Exposure–response plots of DKD exhibited a near-linear trend for PM_2.5_, NO_2_, and NO*_X_* within UK and a potential exponential trend for PM_2.5_ over the global range. Findings from this study would help guide the prevention of DKD.

# Abstract: 5. Artificial Intelligence Diagnosis of Respiratory Diseases Based on Electronic Health Record AI Diagnosis of Respiratory Disease

**Authors:** Tao Yang, Hengrui Liang, Luming Chen, Yifan Qi, Lizong Deng, Jianxing He, Taijiao Jiang

**Affiliations:** ^1^ Guangzhou Medical University, Guangzhou 510182, China.; ^2^ Department of Thoracic Oncology and Surgery, China State Key Laboratory of Respiratory Disease and National Clinical Research Center for Respiratory Disease, First Affiliated Hospital of Guangzhou Medical University, Guangzhou 510120, China.; ^3^ Guangzhou Laboratory, Guangzhou 510005, China.; ^4^ Institute of Systems Medicine, Chinese Academy of Medical Sciences and Peking Union Medical College, Beijing100005, China.; ^5^ Suzhou Institute of Systems Medicine, Suzhou 215123, China.; * Address correspondence to: taijiao@ibms.pumc.edu.cn (T.J.); drjianxing.he@gmail.com (J.H.); denglizong@hotmail.com (L.D.); † These authors contributed equally to this work.

**Background:** Respiratory diseases exhibit a complex disease spectrum compared to diseases affecting other systems and contribute to the highest global disease burden. Despite sharing similar symptoms, various respiratory diseases possess distinct characteristics and necessitate different treatment approaches. Enhancing the prevention and early diagnosis of respiratory diseases has been identified as a crucial recommendation by the Forum of International Respiratory Societies (FIRS). Determining a patient’s underlying disease based on textual descriptions of early symptoms in electronic health records (EHRs) may improve disease prognosis. Consequently, developing an intelligent diagnostic system for specific respiratory diseases based on EHRs is essential.

**Objectives:** To improve the prevention and early diagnosis of respiratory diseases, we developed an intelligent diagnosis model based on deep phenotyping of respiratory department EHRs.

**Methods:** We conducted a retrospective study and obtained EHRs from 61,199 respiratory patients from the First Affiliated Hospital of Guangzhou Medical University, including multiple respiratory system diseases. We use the algorithm based on deep learning to identify the clinical features in the EHRs and then use the PIAT algorithm to recognize the attributes of these clinical features to build a fine-grained clinical phenotype representation. In this study, we developed and evaluated a machine learning-based intelligent diagnostic model, named “LungDiag,” that first attempts to utilize deep phenotyping of EHRs for real-world disease prediction.

**Results:** The deep learning-based approach we used for named entity recognition in EHRs of respiratory diseases was an important first step in developing a diagnostic system. The deep learning performed well in entity recognition, achieving an average precision of 0.883, recall of 0.819, and F1 score of 0.899 for all six types of entities. Based on these entity types, we constructed a fine-grained semantic information model that was applied to the intelligent diagnosis model LungDiag for respiratory diseases. LungDiag demonstrated high diagnostic accuracy with an average precision, recall, and F1 score of 0.767, 0.679, and 0.713, respectively, for top 1 diagnosis, and an average precision of 0.965, recall of 0.895, and F1 score of 0.926 for top 3 diagnoses (Table 1). The ROC-AUC analysis showed that LungDiag had an average AUROC of 0.952 (95% CI: 0.951 to 0.953) among all diseases in the test set.

**Table 1.** LungDiag evaluates the diagnostic performance for the top 1 and top 3 in the differential diagnosis of various respiratory diseases

Disease	AUC(95% CI)	Top 1			Top 3		
		Precision	Recall	F1 score	Precision	Recall	F1 score
COPD	0.941(0.940–0.942)	0.692	0.934	0.794	0.933	0.996	0.964
Bronchial asthma	0.957(0.955–0.959)	0.823	0.617	0.706	0.967	0.875	0.918
Bronchiectasis	0.941(0.941–0.943)	0.753	0.622	0.682	0.954	0.916	0.935
Airway stenosis	0.981(0.978–0.984)	0.769	0.649	0.704	0.983	0.753	0.853
Pulmonary hypertension	0.961(0.960–0.962)	0.712	0.534	0.610	0.940	0.716	0.813
Lung space-occupying lesions	0.952(0.951–0.953)	0.805	0.748	0.775	0.971	0.949	0.960
Pulmonary infectious diseases	0.844(0.842–0.845)	0.702	0.566	0.627	0.965	0.985	0.975
Pleural disease	0.969(0.968–0.971)	0.793	0.544	0.645	0.987	0.969	0.975
Interstitial lung disease	0.985(0.985–0.986)	0.851	0.895	0.873	0.982	0.895	0.926

**Conclusion:** We used a machine learning model to develop an intelligent diagnosis system for respiratory diseases and achieved satisfied performance. This study may help physicians deal with a large number of inpatient records and could potentially provide clinical decision support in diagnostic uncertain cases.

# Abstract: 6. Artificial Intelligence-Enhanced ECGs Identify Patients with Coronary Artery Disease Requiring Revascularization

**Authors:** Peng Wang, Shijia Geng, Shenda Hong, Kangyin Chen

**Affiliations:** ^1^ Tianjin Key Laboratory of Ionic-Molecular Function of Cardiovascular Disease, Department of Cardiology, Tianjin, China.; ^2^ Institute of Cardiology, Second Hospital of Tianjin Medical University, Tianjin, China.; ^3^ HeartVoice Medical Technology, Hefei, China.; ^4^ National Institute of Health Data Science, Peking University, Beijing, China.; ^5^ Institute of Medical Technology, Health Science Center of Peking University, Beijing, China.; * Address correspondence to: hongshenda@pku.edu.cn (S.H.); chenkangyin@vip.126.com (K.C.)

**Background:** With over 10 million Chinese patients suffering from coronary artery disease, accurately determining which individuals require revascularization is a difficult task for medical professionals. This study was aimed to develop an artificial intelligence (AI) model with features extracted from electrocardiograms (ECGs) and clinical information to identify patients who require revascularization.

**Methods:** We collected data retrospectively from 1082 patients with coronary artery disease at the Second Hospital of Tianjin Medical University. The patients were divided into two groups: coronary revascularization required (CRR) group (*n* = 132) and no coronary revascularization required (NCRR) group (*n* = 950). Patients in the CRR group met one of the following: (a) Quantitative coronary angiography measurements showed stenotic lesions on one or more major vessels (left main stem, anterior descending branch, left circumflex, right coronary artery) or their major branches (≥2.5 mm in diameter) with ≥90% diameter stenosis; (b) two interventionists unanimously concluded that the stenotic lesions (diameter stenosis ≥50% and <90% in major vessels or branches) required revascularization based on the quantitative coronary angiography and clinical information. Patients in the NCRR group were diagnosed after excluding obstructive coronary lesions (stenosis < 50%). We collected ECGs, as well as a variety of clinical data including patient demographics, medical history, and laboratory test results, and extracted morphological and heart rate variability (HRV)-related features based on digital ECGs that had been processed with noise reduction. After imputing the missing data, we selected significant clinical and ECG characteristics using LASSO regression. Five machine learning models are tested, including logistic regression, random forest, plain Bayesian, support vector machine, and LightGBM. We split the data into training and test sets in an 8:2 ratio and conducted experiments using only ECG morphological and HRV-related features, as well as combining them with clinical features. After evaluating and comparing machine learning models using ROC curves, accuracy, sensitivity, specificity, and F1 scores, we also explained the models with the SHAP analysis.

**Results:** Patients who required revascularization were significantly older than those who did not (*P* < 0.001). The gender differences were not significant between the two groups. LDL-C, high-sensitivity C-reactive protein, glucose, and NT-proBNP indexes were higher in the revascularization group, while HDL-C and total protein levels were lower. ECG machine auto-calculated parameters and HRV parameters did not differ significantly between the groups not requiring revascularization and those requiring revascularization (*P* > 0.05), but 20 variables showed significant differences in morphological characteristics (*P* < 0.05). A total of 46 significant features were selected using LASSO regression. The features included 23 ECG morphological and HRV-related features and 23 clinical features. Based on AUC, the LightGBM model performed best. If only ECG features were used for modeling, the LightGBM model had an AUC of 0.786, accuracy of 0.776, sensitivity of 0.719, specificity of 0.733, and F1 score of 0.812. However, when the clinical features were added, the AUC increases to 0.949, with an accuracy of 0.834, sensitivity of 0.929, specificity of 0.912, and F1 score of 0.954 (Fig. 4).

**Fig. 4.** A graphical summary of the methods and results of the study.

**Conclusion:** In the present study, we constructed a high-efficiency AI-enhanced ECG model to diagnose patients requiring coronary revascularization via machine learning on the basis of ECG and other clinical information. In the future, we will validate the findings in larger-scale trials.

# Abstract: 7. Artificial Intelligence-Based Model for Relapse in Acute Myeloid Leukemia Patients following Haploidentical Hematopoietic Cell Transplantation

**Authors:** Shuang Fan, Shen-Da Hong, Qi Wen, Hao-Yang Hong, Xiao-Hui Zhang, Lan-Ping Xu, Yu Wang, Chen-Hua Yan, Huan Chen, Yu-Hong Chen, Wei Han, Feng-Rong Wang, Jing-Zhi Wang, Xiao-Jun Huang, Xiao-Dong Mo

**Affiliations:** ^1^ Peking University People’s Hospital, Peking University Institute of Hematology, National Clinical Research Center for Hematologic Disease, BeijingKey Laboratory of Hematopoietic Stem Cell Transplantation, Beijing, China.; ^2^ National Institute of Health Data Science, Peking University, Beijing 100191, China.; ^3^ Institute of Medical Technology, Peking University Health Science Center, Beijing, China.; ^4^ Peking-Tsinghua Center for Life Sciences, Academy for Advanced Interdisciplinary Studies, Peking University, Beijing, China.; ^5^ Research Unit of Key Technique for Diagnosis and Treatments of Hematologic Malignancies (2019RU029), Chinese Academy of Medical Sciences, Beijing, China.; * Address correspondence to: moxiaodong@pkuph.edu.cn; † These authors contributed equally to this work.

**Background:** Allogeneic hematopoietic stem cell transplantation (HSCT) is the most important curative therapy for acute myeloid leukemia (AML), and haploidentical related donor (HID) has become one of the most important alternative donors. However, elapse is one of the most critical causes of transplant failure in AML patients receiving HID HSCT.

**Objectives:** To improve decision-making and determination of candidacy for more intensive relapse prophylaxis, a preoperative prediction model for relapse is necessary. Thus, we aimed to develop an artificial intelligence (AI)-based predictive model for post-transplant in AML patients receiving HID HSCT.

**Methods:** This retrospective study included consecutive AML patients (≥12 years old) receiving HID HSCT in complete remission (CR). We randomly selected 70% of the entire population (*n* = 665) as the training cohort for developing machine learning model and nomogram, which were both evaluated in the remaining 30% patients (validation cohort, *n* = 286). The model was also validated in an independent cohort (*n* = 220) and the clinical practice of 5 experienced clinicians. In addition, the calibration curve and decision curve were plotted for identifying the usability of nomogram. We also validated the discrimination and clinical usefulness by applying the nomogram into clinical use. This work was supported by the National Key Research and Development Program of China (2022YFA1103300 and 2022YFC2502606), Major Program of the National Natural Science Foundation of China (82293630), the Key Program of the National Natural Science Foundation of China (81930004), the National Natural Science Foundation of China (82170208, 82200239, and 62102008), CAMS Innovation Fund for Medical Sciences (2019-I2M-5-034 and 2022-I2M-C&T-B-121), and the Fundamental Research Funds for the Central Universities.

**Results:** Five variables were included in the final model (AML risk category, number of courses of induction chemotherapy for first CR, disease status, measurable residual disease before HSCT, and blood group disparity). A nomogram was designed in training cohort based on the machine learning model, and the validation cohort showed that the concordance index was 0.707 (95% CI: 0.645 to 0.770). The Hosmer–Lemeshow test showed a good fit of this model (*P* = 0.205). The calibration curve was close to the ideal diagonal line, and the decision curve analysis showed significantly better net benefit in this model (Fig. 5). The reliability of our prediction nomogram was proved in validation cohort. The optimal cutoff value of the total nomogram scores was determined to be 95, and the patients could be separated into low- and high-risk groups.

**Fig. 5.** A summary of the results of the machine learning model and prediction nomogram.

**Conclusion:** In summary, we first established a machine learning model and prediction nomogram to predict the relapse of AML patients receiving HID HSCT in CR, which can be popularized easily, can help to provide risk stratification-directed prophylaxis, and may further decrease the risk of relapse. In the future, prospective studies with independent cohorts can further confirm the efficacy of our prediction nomogram.

# Abstract: 8. Association between Coffee Consumption and Risk of Incident Depression and Anxiety: Exploring the Benefits of Moderate Intake

**Authors:** Jiahao Min, Zuolin Lu, Yabing Hou, Hongxi Yang, Xiaohe Wang, Chenjie Xu

**Affiliations:** ^1^ School of Public Health, Hangzhou Normal University, Hangzhou, China.; ^2^ Department of Epidemiology, Erasmus University Medical Center, Rotterdam, Netherlands.; ^3^ Yanjing Medical College, Capital Medical University, Beijing, China.; ^4^ School of Basic Medical Sciences, Tianjin Medical University, Tianjin, China.; * Address correspondence to: xuchenjie@hznu.edu.cn

**Background:** Moderate coffee consumption has been shown to be associated with a lower risk of cardiovascular disease, cancer, and mortality. However, the association between coffee consumption and depression and anxiety remains unclear. In particular, it is largely unknown whether the association between coffee intake and mental disorders is modified by the type of coffee (instant, ground, and decaffeinated coffee) and coffee additives (milk, sugar-sweetened, and artificial-sweetened).

**Objectives:** We aimed to examine the associations of coffee consumption with incident depression and anxiety, and to assess whether the association differed by coffee subtypes or additives.

**Methods:** In this prospective cohort study, coffee consumption, coffee subtypes, and coffee additives were measured using touchscreen questionnaires during 2006–2010 in 146,656 participants (mean age: 55.9 years, female: 56.5%) with valid information on mental health in the UK Biobank. At follow-up, incident depression and anxiety were measured in 2016 using the Patient Health Questionnaire (PHQ)-9 and the Generalised Anxiety Disorder Assessment (GAD)-7, respectively. We used logistic regression models and restricted cubic splines to assess the associations of coffee consumption, coffee subtypes, and coffee additives with incident depression and anxiety, adjusting for age, sex, ethnicity, education, Townsend deprivation index (TDI), body mass index (BMI), smoking status, alcohol intake, tea intake, diet score, sleep duration, history of hypertension, history of diabetes, and time span.

**Results:** Approximately 80.7% of participants reported consuming coffee, and most drank 2 to 3 cups per day (41.2%). We found J-shaped associations between coffee consumption and both incident depression and anxiety, with the lowest risk of mental disorders occurring at around 2 to 3 cups per day (*P* for nonlinear < 0.001). Relative to non-coffee consumers, the multivariable-adjusted odds ratios (95% confidence intervals) of incident depression for participants drinking 1 or fewer, 2 to 3, 4 to 5, 6 to 7, 8 to 9, and 10 or more cups per day were 0.91 (0.84 to 0.99), 0.87 (0.81 to 0.94), 0.86 (0.78 to 0.95), 1.04 (0.90 to 1.20), 1.30 (1.03 to 1.64), and 1.33 (1.02 to 1.73), and the corresponding estimates of incident anxiety were 0.89 (0.82 to 0.96), 0.90 (0.83 to 0.97), 0.89 (0.81 to 0.99), 1.03 (0.89 to 1.19), 1.19 (0.93 to 1.52), and 1.48 (1.15 to 1.92). Results of depression for participants who drank 2 to 3 cups of ground coffee, milk-coffee, or unsweetened coffee were 0.79 (0.70 to 0.89), 0.77 (0.70 to 0.84), and 0.78 (0.71 to 0.86), respectively. The association between 2 to 3 cups of ground coffee, milk-coffee, or unsweetened coffee and anxiety was largely consistent.

**Conclusion:** We found that moderate daily coffee consumption, especially at 2 to 3 cups of ground coffee, milk-coffee, or unsweetened coffee, was associated with a lower risk of incident depression and anxiety. Our findings highlight that moderate daily coffee consumption could be recommended as part of a healthy lifestyle to improve mental health (Fig. 6).

**Fig. 6.** A graphical summary of the study’s methods, results, and conclusion.

**Acknowledgments:** This study was conducted using the UK Biobank resource (application 79095). We want to express our sincere thanks to the participants of the UK Biobank and the members of the survey, development, and management teams of this project.

**Funding:** This work was supported by the National Natural Science Foundation of China (grant number 72204071), Zhejiang Provincial Natural Science Foundation of China (grant number LY23G030005), and Scientific Research Foundation for Scholars of HZNU (grant number 4265C50221204119). None of the funder had any role in the design and conduct of the study; the collection, management, analysis, and interpretation of the data; and the preparation, review, or approval of the manuscript.

**Author Contributions:** J.M. and C.X. contributed to the conception, study design, and ideas. J.M. and C.X. collected and assembled the data. J.M. performed the statistical analysis and results interpretation. J.M. wrote the first and successive drafts of the manuscript. Z.L., Y.H., H.Y., X.W., and C.X. revised the manuscript for important intellectual content. C.X. obtained funding. X.W. and C.X. provided administrative, technical, and logistic support. All authors reviewed the manuscript and approved the final version.

# Abstract: 9. Association between Urinary Sodium Excretion and Dementia Risk

**Authors:** Ying Li, Nana Peng, Shiyu Wang, Bingli Li, Yiwen Jiang, Di Liu, Fuxiao Li, QingPing Yun, Tengfei Lin, Peng Wu, Jiaxin Cai, Qi Feng, Zhirong Yang, Feng Sha, Jinling Tang

**Affiliations:** ^1^ Shenzhen Institute of Advanced Technology, Chinese Academy of Sciences, Shenzhen, Guangdong, China.; ^2^ Nottingham Ningbo China Beacons of Excellence Research and Innovation Institute, University of Nottingham Ningbo China, Ningbo, China.; ^3^ Institute of Child Health, University College London, London, UK.; ^4^ Primary Care Unit, School of Clinical Medicine, University of Cambridge, Cambridge, UK.; ^5^ Clinical Data Center, Guangzhou Women and Children’s Medical Center, Guangzhou Medical University, Guangzhou, Guangdong, China.; ^6^ Division of Epidemiology, The JC School of Public Health & Primary Care, The Chinese University of Hong Kong, Hong Kong, China.; * Address correspondence to: fengsha@siat.ac.cn (F.S.); zr.yang@siat.ac.cn (Z.Y.)

**Background:** As a surrogate for sodium intake, sodium excretion has been associated with increased risk of cardiovascular disease but the impact of sodium excretion on the risk of dementia is largely unknown.

**Objectives:** To examine the association between sodium excretion and the incidence of all-cause dementia, Alzheimer’s disease, and vascular dementia.

**Methods:** We included and followed up UK Biobank participants with no dementia at baseline. We estimated 24-h urinary sodium excretion (accounting for nearly 93% of sodium intake) based on age, BMI, sodium, potassium, and creatinine in spot urine. We used Cox regression to quantify the association and the dose–response relationship between estimated 24-h urinary sodium excretion and the risk of all-cause dementia, Alzheimer’s disease, and vascular dementia. We further examined the interaction between estimated 24-h urinary sodium excretion and age, apolipoprotein E (APOE) ε4 status, and estimated glomerular filtration rate (eGFR).

**Results:** Among 468,277 eligible participants (213,960 men) with a mean age of 56.5 years, the mean estimated 24-h urinary sodium excretion was 3.0 g (men: 3.6 g; women: 2.50 g). Over a mean follow-up of 13.6 years, 8,071 (1.7%) participants developed all-cause dementia, 3,860 (0.8%) Alzheimer’s disease, and 1,896 (0.4%) vascular dementia. The sodium excretion was inversely associated with all-cause dementia in a nonlinear dose–response manner in the overall population and men but in a linear manner in women. Compared to quartile 1 of the sodium excretion (lowest), the hazard ratio (HR) of all-cause dementia was 0.76 [95% confidence interval (CI), 0.68 to 0.85], 0.85 (95% CI, 0.76 to 0.95), and 0.87 (95% CI, 0.76 to 0.99) for quartile 4 (highest) in the overall population, men, and women, respectively. For vascular dementia, a nonlinear dose–response relationship was found with sodium excretion in the overall population and men but no association was observed in women. We did not find any association of sodium excretion with Alzheimer’s disease in the overall population or by sex. These results remained robust in various subgroup and sensitivity analyses. Age, APOE ε4 status, and eGFR significantly interacted with estimated 24-h urinary sodium excretion in the association with all-cause dementia (Fig. 7).

**Fig. 7.** A graphical summary of the methods and results of the study.

**Conclusion:** Sodium excretion was associated with a lower risk of all-cause dementia and vascular dementia. These findings may denote caution when advocating sodium reduction in the middle-aged and elderly population.

# Abstract: 10. Association of Household Size and Intergenerational Structure with the Onset of Depression among Middle-Aged and Older Adults in China

**Authors:** Jun Ma, Wenwen Liu, Yangfan Chai, Jiayu Wang, Guilan Kong

**Affiliations:** ^1^ National Institute of Health Data Science, Peking University, Beijing, China.; ^2^ Advanced Institute of Information Technology, Peking University, Hangzhou, Zhejiang, China.; * Address correspondence to: Guilan.kong@hsc.pku.edu.cn

**Background:** According to the data of Chinese national census from 1982 to 2015, Chinese family structure has undergone tremendous changes over the past few decades, resulting in decreasing household size and incomplete or skipped-generation families [1]. However, the association of household size and intergenerational structure with depression remains controversial [2,3]. Especially in multi-generation households, a cohort study [2] found that they would had 6% higher odds of experiencing depressive symptoms, but the cross-sectional study of elderly people in rural areas of Anhui province in China showed that they had better psychological well-being [3]. Moreover, there is a lack of research focusing on the relationship of household size, intergeneration structure, and the onset of depression among Chinese middle-aged and older adults.

**Objectives:** This study aimed to investigate the association between household size, intergenerational structure, and the onset of depression among Chinese middle-aged and older adults.

**Methods:** The survey data of four waves (2011, 2013, 2015, and 2018) from the China Health And Retirement Longitudinal Study (CHARLS) were used as data source. Participants who were enrolled in 2011 and were followed up at least once later were included, and those without depression or household structure data were excluded. Two cohorts were designed in this study. Cohort 1 comprised all included participants and was used to investigate the trend of household size, intergenerational structure, and depression. Cohort 2 comprised participants who had no depressive symptoms in 2011 and was used to explore the association of household size and intergeneration structure with the onset of depression. The depressive symptoms were evaluated using the Center for Epidemiological Studies Depression (CES-D) scale. Household size and intergenerational structure were defined using family member relationships, residential arrangements, and the respondents’ marital status. Taking into account time-varying confounding effect, a time-dependent Cox proportional hazards model was used to estimate the association in cohort 2.

**Results:** In total, 6,829 participants were included in cohort 1 and 4,257 participants were in cohort 2. The household size of Chinese middle-aged and older adults gradually decreased over time. The average household size was 3.48 in 2011 and then 2.25 by 2018. The proportion of empty-nest couple, solitary, and one-generation households was increasing, which was 26.29%, 8.20%, and 34.49%, respectively, in 2011 and then 53.45%, 20.48%, and 73.93%, respectively, in 2018. The proportion of participants with depressive symptoms showed a U-shape with 2013 (33.81%) as the turning point, and it was always lower in the males than in the females. By 2018, the proportion of participants with depression reached 40.59%, with 32.35% for males and 48.51% for females. As shown in Table 1, people with larger household size were more likely to suffer from depressive symptoms (HR = 1.05, *P* = 0.002). Compared with one-generation households, three-generation households (HR = 1.30, *P* < 0.001) had higher risk of developing depressive symptoms. In one-generation households, compared with empty-nest couples, the solitary (HR = 1.28, *P* = 0.028) had higher risk of depression incidence. In the female population, people with larger household size (HR = 1.07, *P* < 0.001) or in three-generation households (HR = 1.31, *P* = 0.004) had the higher risk of the depression occurrence. However, there were no similar findings in the male population.

**Table 1.** Hazard ratios (HRs) and 95% confidence intervals (CIs) for new-onset depressive symptoms associated with household size and intergenerational structure

Predictor	Depressive symptoms					
	Overall		Male		Female	
	Crude HR (95% CI)	*P* value	Crude HR (95% CI)	*P* value	Crude HR (95% CI)	*P* value
Household size	1.05 (1.02, 1.08)	0.002	1.02 (0.97, 1.06)	0.449	1.07 (1.03, 1.11)	<0.001
Intergenerational structure						
One-generation households	1 [Ref.]		1 [Ref.]		1 [Ref.]	
Two-generation households	1.11 (0.98, 1.26)	0.110	1.10 (0.92, 1.31)	0.313	1.08 (0.90, 1.29)	0.430
Three-generation households	1.30 (1.14, 1.48)	<0.001	1.18 (0.98, 1.44)	0.088	1.31 (1.09, 1.57)	0.004
Four-generation households	1.17 (0.74, 1.85)	0.511	0.66 (0.27, 1.61)	0.365	1.51 (0.88, 2.59)	0.136
Empty nest						
Empty-nest couple	1 [Ref.]		1 [Ref.]		1 [Ref.]	
Solitary	1.28 (1.03, 1.58)	0.028	1.79 (1.32, 2.43)	<0.001	0.84 (0.62, 1.14)	0.260

**Conclusion:** The intergenerational structure of middle-aged and older population in China has gradually shifted from multi-generation toward one-generation households. Overall, this shift has a protective effect on the onset of depression. However, in one-generation households, the risk of depression in the solitary is higher than that in empty-nest couples with statistical significance. Therefore, special attention should be paid to the mental health of Chinese middle-aged and older people, especially the solitary and those who live in multi-generation households.

**AcknowledgmentsFunding:** This study was supported by grants from Beijing Municipal Science & Technology Commission (grant no. 7212201), Humanities and Social Science Project of Chinese Ministry of Education (grant no. 22YJA630036), and Zhejiang Provincial Natural Science Foundation of China (grant no. LZ22F020014).

## References

[1] Li T, Fan W, Song J. The household structure transition in China: 1982-2015. Demography. 2020;57(4):1369–1391.32524533 10.1007/s13524-020-00891-7

[2] Dong X, Ng N, Santosa A. Family structure and depressive symptoms among older adults in China: A marginal structural model analysis. J Affect Disord. 2023;1(324):364–369.10.1016/j.jad.2022.12.06736566935

[3] Silverstein M, Cong Z, Li S. Intergenerational transfers and living arrangements of older people in rural China: Consequences for psychological well-being. J Gerontol B Psychol Sci Soc Sci. 2006;61(5):S256–S266.16960239 10.1093/geronb/61.5.s256

# Abstract: 11. Associations between Hemoglobin Levels among Children Aged under Five and Ambient Fine Particles Are Modified by the Source Profile

**Authors:** Pengfei Li, Jingyi Wu, Tao Xue

**Affiliations:** ^1^ Institute of Reproductive and Child Health/National Health Commission Key Laboratory of Reproductive Health and Department of Epidemiology and Biostatistics, School of Public Health, Peking University Health Science Centre, Beijing 100191, China.; ^2^ Advanced Institute of Information Technology, Peking University, Hangzhou 311215, China.; ^3^ National Institute of Health Data Science, Peking University, Beijing 100191, China.; ^4^ Center for Environment and Health, Academy for Advanced Interdisciplinary Studies, Peking University, Beijing, China.; * Address correspondence to: txue@hsc.pku.edu.cn; † These authors contributed equally to this work.

**Background*:*** A low hemoglobin level is a key indicator of malnutrition and a major health issue among children in low- and middle-income countries (LMICs). Although fine particulate matter (PM_2.5_) has been associated with childhood anemia or a low hemoglobin level in recent studies, large-population studies from multiple LMICs are rare. Furthermore, PM_2.5_ produced by different sources has distinct chemical compositions and the effects of PM_2.5_ from various sources on hemoglobin remain unknown.

**Objectives*:*** To investigate the associations of child hemoglobin levels with long-term exposure to source-specific ambient PM_2.5_.

**Methods*:*** The hemoglobin levels of 36,675 children aged <5 years were determined using Demographic and Health Surveys data collected in 11 LMICs [including 9 Asian and African LMICs (AA-LMICs), a Latin America low-income country (i.e., Haiti), and a Europe lower-middle-income country (i.e., Albania)] during 2017. Long-term exposure to PM_2.5_ was assessed in terms of annual concentrations using a state-of-the-art method, which combined chemical transport model (CTM) simulations with satellite remote sensing measurements and in situ monitoring data. Fractions for 20 source-specific PM_2.5_ components were estimated by CTM simulation in 2017. We explored associations of hemoglobin levels with total PM_2.5_ exposure or source-specific PM_2.5_ exposure using generalized linear regressions, and we derived a joint exposure–response function using a ridge regression model of all 20 source-specific components for multiple-source analysis. Using real-world exposure data of PM_2.5_ mixtures from 88 AA-LMICs as the modeled scenarios, we quantified the relative change (toxicity) or absolute change (overall effect) in hemoglobin with respect to a specific combination of the 20 source terms to determine how the source profile influenced the PM_2.5_–hemoglobin association.

**Results*:*** A 10 μg/m^3^ increase in PM_2.5_ exposure was associated with a −0.25 g/l [95% confidence interval (CI): −0.84 to 0.35] change in hemoglobin among children, after fully adjusting for multiple confounders. In single-source analysis, PM_2.5_ produced by solvents (SLV), non-road transportation, industrial coal combustion (INDcoal), road transportation (ROAD), commercial combustion, waste handling and disposal (WST), or agriculture emissions was significantly associated with a decrease in hemoglobin level, and the changes in hemoglobin per 1 μg/m^3^ increase in PM_2.5_ were −22.71 g/l (95% CI: −37.59 to −7.84), −19.87 g/l (95% CI: −36.02 to −3.71), −3.59 g/l (95% CI: −6.18 to −1.00), −1.27 g/l (95% CI: −2.27 to −0.27), −5.02 g/l (95% CI: −9.37 to −0.68), −1.56 g/l (95% CI: −2.13 to −0.99), and −1.15 g/l (95% CI: −2.29 to −0.01), respectively. In the multiple-source model, the associations for SLV (1 μg/m^3^ change in hemoglobin: −10.34 g/l, 95% CI: −14.88 to −5.91), INDcoal (−0.51 g/l, 95% CI: −9.25 to −0.08), ROAD (−0.50 g/l, 95% CI: −6.96 to −0.29), and WST (−0.34 g/l, 95% CI: −4.38 to −0.23) remained significant. The scenario analysis showed that the decreases in hemoglobin attributable to the PM_2.5_ mixtures were co-determined by the concentrations and their source profiles (Figure 8). The largest PM_2.5_-related change in hemoglobin was −10.25 g/l (95% CI: −15.54 to −5.27) for a mean exposure of 61.01 μg/m^3^ in India, followed by a change of −7.58 g/l (95% CI: −12.48 to −2.94; 44.85 μg/m^3^) in China. In both countries, INDcoal was the main contributor to the PM_2.5_-related decrease in hemoglobin.

**Fig. 8.** Country-specific variations in (A) toxicity or (B) effect (scatters with error bars) of a PM_2.5_ mixture on hemoglobin across 88 AA-LMICs, as determined by ridge regression of multiple pollutants. In (A), column heights indicate proportions of source-specific PM_2.5_. In (B), column heights indicate concentrations of source-specific PM_2.5_ and widths indicate population proportions in different countries. The following 20 PM_2.5_ sources were considered for analysis: road transportation (ROAD); non-road or off-road transportation (NRTR); industrial coal combustion (INDcoal); industrial non-coal combustion (INDother); waste handling and disposal (WST); anthropogenic fugitive, combustion, and industrial dust (AFCID); energy production from coal combustion only (ENEcoal); energy production by all non-coal combustion (ENEother); international shipping (SHP); commercial combustion (RCOC); residential combustion of solid biofuel only (RCORbiofuel); residential combustion of coal only (RCORcoal); residential combustion of all other energy types (RCORother); all other combustion (RCOO); agricultural waste burning from the GFED fires inventory (GFEDagburn); other open fires from the GFED fires inventory (GFEDoburn); agriculture (AGR); solvents (SLV); windblown dust (WDUST); and all remaining sources, including volcanic SO2, lightning NOx, biogenic soil NO, ocean emissions, biogenic emissions, very-short-lived iodine and bromine species, and decaying plants (OTHER).

**Conclusion*:*** The association between PM_2.5_ and a decrease in hemoglobin was affected by variations in PM_2.5_ source profiles. PM_2.5_ from some anthropogenic sources significantly contributed to childhood malnutrition. Source-oriented interventions are warranted to protect children in LMICs from air pollution.

**Funding:** This work was supported by the National Natural Science Foundation of China (42175182, to T.X.), Energy Foundation (G-2208-34045, G-2107-33169, and R-2109-33379, to T.X.), and PKU-Baidu Fund (2020BD031, to T.X.).

# Abstract: 12. Clustering Irregularly Sampled Medical Time Series Data with Time-Aware Dynamic Time Wrapping

**Authors:** Ji Deng, Shenda Hong

**Affiliations:** ^1^ Department of Computer Science, Beijing University of Posts and Telecommunications, Beijing, China.; ^2^ National Institute of Health Data Science, Peking University, Beijing, China.; * Address correspondence to: hongshenda@pku.edu.cn

**Background:** Temporal medical data consist of electronic medical records (EMRs) and physiological signals. A significant proportion of temporal medical data is irregularly sampled, primarily due to factors such as device malfunctions, patient health status change, and medical staff factors. Data-driven clustering on irregularly sampled medical time series (ISMTS) to form patient subgroups is helpful for outcome prediction, patient subtyping, drug recommendation, and so on.

**Objectives:** In this work, we aim to propose a novel clustering method specifically designed for analyzing ISMTS data. Currently, most existing clustering methods only consider regular sampled data and have difficulties when directly applied to ISMTS data. First, imputation methods are required to handle missing values within the ISMTS data before being applied to these methods. Second, sampling intervals are often associated with clinical meanings, which cannot be ignored. For example, the medical staff may measure a patient’s vital signs with a low frequency when the patient’s status is stable and increase the frequency when closer monitoring is necessary. The work for regular sampled data does not leverage such valuable information encoded in sampling intervals.

**Methods:** We propose time-aware dynamic time wrapping (T-DTW) method to address the above challenges (Fig. 9). T-DTW offers several advantages, including the ability to handle ISMTS data without the need for imputing missing values, while taking into account the underlying sampling intervals. T-DTW builds upon the foundation of dynamic time wrapping (DTW), a method commonly used to measure the distance of two time series by the total cost of the warping path. However, T-DTW goes beyond DTW by incorporating differences in sampling intervals around each sampling value. This additional factor is weighted by a hyperparameter when calculating the distance between time series. Thus, T-DTW can appropriately reflect the time series’ similarity when they are sampled irregularly. Two points with similar sampling intervals may be matched when measured by T-DTW. The distance between each pair of points *(p*, *q)* in two time series computed by T-DTW is *d = |vp − vq| + a|ip − iq|*, where *vp* and *vq* represent the variable values of points *p* and *q*, respectively, and *i_p_* and *i_q_* represent the corresponding sampling intervals around points *p* and *q*. The parameter *a* is a hyperparameter that weights the contribution of the sampling interval difference to the overall distance calculation.

**Fig 9.** Our framework.

**Experiments:** We perform experiments on the PhysioNet Challenge 2019 dataset, which consists of thousands of monitoring data of real-world ICU patients, along with their corresponding sepsis outcome labels. For the variable selection, we calculate the missing rate and opted to include only those below 0.8. This leads to heart rate (HR), systolic BP (SBP), diastolic BP (DBP), mean arterial pressure (MAP), respiration rate (Resp), temperature (Temp), and pulse oximetry (O2Sat). We employ both DTW and T-DTW with *a* ranging from 0.1 to 0.5 for distance matrix calculation, transform distance matrix into a similarity matrix using Gaussian kernel, and perform spectral clustering. We assess the agreement between the obtained clustering label and the patient’s sepsis label, a binary label indicating whether the patient suffers sepsis, utilizing 6 metrics, namely, chi-square, adjusted rand score, mutual info score, adjusted mutual info score, normalized mutual info score, and completeness score.

**Results:** The results show that T-DTW outperforms DTW across all 6 evaluation metrics for 4 of 7 variables (SBP, DBP, Resp, and Temp). For the remaining 3 variables, we further select time series with missing rate between 0.2 and 0.8, and repeat the clustering process mentioned above. T-DTW demonstrated superior performance compared to DTW on 2 of 3 variables (HR and MAP). The results underscore the potential of T-DTW for enhancing clustering performance on medical time series characterized by irregular sampling.

Our code is publicly available at https://github.com/dengji1/Time-aware-DTW-code/tree/master.

# Abstract: 13. Deep Learning-Based Detection of Obstructive Sleep Apnea through Combined ECG and ECG-Derived Respiration Signals

**Authors:** Yaomin Wang, Chenzhe Jin, Yuxi Zhou, Shenda Hong

**Affiliations:** ^1^ School of Computer Science and Engineering, Tianjin University of Technology, Tianjin, China.; ^2^ DCST, BNRist, RIIT, Institute of Internet Industry, Tsinghua University, Beijing, China.; ^3^ National Institute of Health Data Science, Peking University, Beijing, China.; ^4^ Institute of Medical Technology, Health Science Center of Peking University, Beijing, China.; * Address correspondence to: joy_yuxi@pku.edu.cn (Y.Z.); hongshenda@pku.edu.cn (S.H.); † These authors contributed equally to this work.

**Background:** Obstructive sleep apnea (OSA) is a prevalent sleep disorder characterized by recurrent episodes of partial or complete upper airway obstruction during sleep. Recent studies have estimated that approximately one billion people worldwide suffer from OSA. Untreated or undiagnosed individuals face an elevated risk of cardiovascular diseases, such as hypertension, heart failure, and stroke. The current gold standard for diagnosing OSA remains overnight polysomnography (PSG), which involves extensive monitoring of subjects wearing multiple sensors throughout the night. However, it is hard to collect PSG data in a home environment. Existing non-invasive and portable home monitoring methods for OSA diagnosis mainly rely on modeling monitoring data from wearable devices such as electrocardiogram (ECG). However, these methods often overlook the crucial respiratory information, which is the most direct parameter for detecting OSA, leading to inaccurate analysis of patients’ OSA risk. Additionally, these approaches neglect the influence of demographic characteristics and other relevant features on the model.

**Objectives:** This paper aims to address above challenges by proposing a deep learning-based OSA diagnostic method that incorporates the combination of ECG and ECG-derived respiration (EDR) signals. It also aims to develop an interpretable nomogram model that considers patient demographics.

**Methods:** First, we extract respiration signals from ECG. This involves extracting R–R intervals from the ECG and employing cubic spline interpolation to derive the EDR. Second, we propose a deep learning model, which takes ECG and EDR as input, and output the probabilities of OSA from ECG and EDR separately. Next, rather than directly concatenating the features extracted from the ECG and EDR signals, we build a logistic regression (LR) to combine the OSA risk predictions derived from the ECG signal, the EDR signal, and the demographic characteristics. Finally, we employ a nomogram to represent the LR model.

**Experiments:** The experiments were performed using the PhysioNet Apnea-ECG dataset, comprising 70 single-lead ECG recordings sampled at a frequency of 100 Hz. In order to solve the problem of insufficient training sample for training a robust deep leaning model, we randomly divide the dataset into the training and testing set in a 8:2 ratio, and then we employ a slide-and-cut strategy to generate more short-term segments of length 12,000 from raw training ECG data and testing ECG data, respectively. To minimize the impact of noisy data, including ECG baseline wander, a low-pass filter was applied during the data preprocessing stage. To assess the agreement between the predicted OSA labels and the actual disease labels of the patients, we employed four evaluation metrics. These metrics primarily consisted of precision, recall, F1 score, and AUC (area under the ROC curve). These metrics were used to measure the accuracy, completeness, and overall performance of the OSA prediction model in classifying the patients, true OSA status.

**Results and Discussion:** The results demonstrated that our model achieved ROC of 0.883 and F1 score of 0.991 after majority voting of all segments, surpassing existing methods in all four evaluation metrics. From the nomogram, we can see that the severity of OSA showed a clear association with age and body weight. Additionally, both the ECG signal and EDR signal played crucial roles in accurately determining the presence of OSA (Fig. 10). These findings suggest that these signals can provide vital OSA-related information, aiding doctors in making precise diagnoses and developing appropriate treatment plans. Furthermore, we employed the UMAP algorithm to visualize the automatically extracted features of our model. The visualization demonstrated the model,s discriminative ability in distinguishing between different classes. Additionally, we leveraged SHAP values to assess the impact of the ECG signal on OSA decision-making. Notably, ECG segments with prominent respiratory disorder features were highlighted in dark red, indicating the model,s automatic focus on data segments significantly associated with OSA. This capability enables the identification of abnormal segments from extensive and lengthy monitoring data, providing doctors with more interpretable information and valuable assistance in OSA diagnosis and decision-making processes.

**Fig. 10.** A graphical summary of the objectives, methods, and results of the study.

# Abstract: 14. Deep Learning-Based Real-Time Predictive Model for the Development of Acute Stroke

**Authors:** Lan Lan, Jiawei Luo, Ling Guan, Rui Li, Yilong Wang

**Affiliations:** ^1^ IT Center, Beijing Tiantan Hospital, Capital Medical University, Beijing 100070, China.; ^2^ West China Biomedical Big Data Center, West China Hospital, Sichuan University, Chengdu 610044, China.; ^3^ Department of Neurology, Beijing Tiantan Hospital, Capital Medical University, Beijing 100070, China.; * Address correspondence to: yilong528@aliyun.com; † These authors contributed equally to this work.

**Abstract Competition Winner – Third Prize**

**Background:** Stroke is the leading cause of death and disability among adults in China. The existing pre-hospital tools are not suitable for stroke recognition during sleep. Prediction models mostly classify and diagnose stroke or predict the probability of stroke occurrence within a specific time range using baseline or characteristics at admission. Although the use of ECG, EEG, and EMG is helpful for the diagnosis of stroke, they cannot be acquired for a long time due to the discomfort caused by electrode sticking to the body.

**Objectives:** There is an urgent need to develop tools that can provide real-time prediction and warning of stroke. This study will use real-time monitoring of respiratory and heart rate, as well as routine lab and nursing indices, to predict stroke in real time.

**Methods:** We adopted a device embedded in a mattress to collect the respiratory and heart rate without feeling the device, which were estimated based on ballistocardiography (BCG) in real time. The patients who were hospitalized and used the mattress at Beijing Tiantan Hospital, Capital Medical University from October 2018 to March 2022 were included. We retained 43 lab indices and 24 nursing indices covering 80% of patients. Respiratory and heart rate, lab, and nursing indices were represented at 1, 48, and 24 h, respectively, where missing data were processed using linear interpolation. First, we input the three temporal features into three transformers, then summarized the three types of features through the full connection layer, and finally output the stroke prediction results with an activation function. All data were divided into training and testing sets with a ratio of 7:3, where 85% of the training set was used for developing the model and 15% was used for tuning parameters. AUROC, F1, precision, and recall were applied to evaluate model performance.

**Results:** This study included a total of 13,112 patients, of which 1,108 had stroke. We established 7 prediction models using different temporal features. For a feature, the prediction performance using lab indices was the best. For two features, lab plus nursing indices performed best. Overall, the prediction model that combines three temporal features performed best (Table 1). The diagnosis of stroke is mainly based on imaging exams. However, due to its high cost and relatively long time from ordering to reporting, images of medical imaging are not suitable for real-time prediction of stroke. In hospital, it can predict stoke in real time based on the latest and historical lab or nursing or respiratory and heart rate information, as this information is collected in real time and the time from ordering to reporting is relatively short in hospital. If it is at home, real-time respiratory and heart rate can be collected through the use of mattresses, which can be used to predict stroke. But its predictive performance is not very good in this study. It may be related to the frequency of data collection. Therefore, we will collect higher frequency respiratory and heart rate. However, this study effectively predicted stroke (recall: 93.1%) by combining three temporal features.

**Table 1.** Prediction performance

Feature	AUROC	F1	Precision	Recall
BCG	0.601	0.156	0.080	0.808
Lab	0.832	0.377	0.221	0.889
Nursing	0.723	0.285	0.125	0.864
BCG + Lab	0.845	0.391	0.227	0.901
BCG + Nursing	0.751	0.302	0.189	0.876
Lab + Nursing	0.862	0.406	0.249	0.915
BCG + Lab + Nursing	0.869	0.410	0.254	0.931

**Conclusion:** The deep learning model using a combination of three temporal features can quickly and effectively predict stroke in hospital. Non-contact respiratory and heart rate monitoring provide the possibility for daily monitoring of stroke among people at home, especially empty nesters.

# Abstract: 15. Design and Implementation of Health Data Management and Governance Platform

**Authors:** Hongan Pan, Qing Li, Pengfei Li

**Affiliations:** ^1^ Advanced Institute of Information Technology, Peking University, Hangzhou 311215, China.; ^2^ National Institute of Health Data Science, Peking University, Beijing 100191, China.; * Address correspondence to: pfli@aiit.org.cn

The management and governance of health data have become increasingly complex due to the proliferation of health data and the emergence of diverse data sources. Although there exist mature specialized disease research platforms and regional health big data platforms in China, these platforms often lack necessary online data processing and analysis modules. Despite various data processing and analysis software tools currently available, there remains a lack of specialized health data management and governance mechanisms and corresponding tools. Medical researchers frequently struggle to conduct health data-driven projects due to high technical barriers to data processing and analysis. Hence, there is an urgent need to establish a comprehensive and intelligent health data management and governance platform for medical researchers.

This study presents Visdata (https://visdata.bjmu.edu.cn/), a platform designed to achieve health data management and governance by facilitating data acquisition and integration while improving data quality and enhancing the efficiency of data analysis.

The basic framework of Visdata platform comprises Data Collector, Data Converter, Data Evaluator, and Visual Analysis System. The workflow of health data management via Visdata platform is as follows: First, the Data Collector, embedded with self-developed data mapping tools, collects and extracts multi-modal health data from various sources like electronic medical records, open health databases, medical literature data, and desensitized data uploaded by platform users. Second, the Data Converter transforms the collected data into standard formats in patient or record dimension. Although this step often involves complex data preprocessing, data cleaning, and natural language processing, the Data Converter provides a range of prepackaged algorithms. With its user-friendly design and interface, medical researchers can make both structured and unstructured data machine-ready without understanding complicated data processing theory or programming techniques. Then, the rule engine-based Data Evaluator plays a crucial role in data quality control. According to the common range of variables, logical relationships and restrictions between variables, the Data Evaluator generates tree expression evaluation rules, which help medical researchers evaluate data quality in terms of standardization, accuracy, completeness, timeliness, and accessibility. Meanwhile, the quality evaluation results will be fed back to the Data Converter, which may start a new round of data processing until the data meet the preset criteria in the Data Evaluator. Finally, the Visual Analysis System helps medical researchers to conduct visual health data analysis reports with packaged machine learning (ML) models. Based on the pre-trained clinical research data of specific diseases and knowledge base of expert advice, these ML models are highly effective in conducting basic data visualization, data description, statistical tests, correlation analysis, survival analysis, and other data mining algorithms commonly used in health field.

As a consequence, the Visdata platform provides researchers with high-quality health data resources and handy data processing and analysis tools, making it easier to conduct clinical research in an efficient and convenient manner.

As the first specialized health data management and governance platform in China, Visdata focuses on clinical practice and provides medical researchers with an integrated and user-friendly platform ecosystem. The Data Collector has the capacity to collect multi-modal and multi-source health data, while the Data Converter and the Data Evaluator work jointly to ensure accuracy and consistency of the collected data. With 30 types of built-in statistical approaches, the Visual Analysis System can inspire scientific research to a large extent. The Visdata platform has the potential to revolutionize health data management and governance in China, primarily by integrating high-quality health data and delivering user-accessible health data governance solutions. As such, the Visdata platform could play a significant role in future health advancement, improving the overall landscape of the health field in China.

# Abstract: 16. Development of a Generalizable Agent-Based Model for Optimizing Emergency Responses of Primary Care Institutions in China

**Authors:** Yipeng Lv, Yan Li

**Affiliations:** College of Public Health, School of medicine, Shanghai Jiaotong University, Shanghai, China.; * Address correspondence to: yanliacademic@gmail.com

**Background:** During the early stage of a pandemic, there is a high risk of covert transmission due to the lack of attention given to cases with mild or moderate symptoms and asymptomatic carriers. This can result in a wide spread of the disease and worsened strain on healthcare resources. Therefore, it is important to optimize the use of primary care networks and improve the disease prevention and control system.

**Objectives:** We aim to develop a generalizable agent-based model for optimizing emergency responses of primary care institutions during the early stage of a pandemic in China. The model can assess the impact of emergency responses on routine primary care services such as chronic disease management. We demonstrate the potential use of the model using a case study based on data from Shanghai, China.

**Methods:** We developed a generalizable agent-based model to simulate emergency responses based on the behavioral characteristics of different populations, the features of pandemic evolution, and the response of primary care institutions, as well as the demand for routine healthcare services (Fig. 11). Our model includes natural person subjects, which are divided into three types based on the characteristics of their activity patterns and have spatial and health status changes. We also designed healthcare institution subjects, including different types with varying health resource and functional positioning attributes, and medical personnel subjects, which include personnel for routine medical services and emergency response. We also designed their interaction methods and state transition rules. As a case study, using data from Shanghai, we simulated hypothetical pandemic prevention and control policies, disease screening, and isolation management. These policies formed five simulation scenarios in our simulation experiments. We collected two major outcomes from the simulation studies: the evolution of epidemic transmission and the impact on routine healthcare services.

**Fig. 11.** The input and output interface of the agent-based model for emergency response of primary care institutions.

**Results:** The agent-based model had acceptable simulation effectiveness and stability in terms of epidemic transmission, health resource utilization, and routine health services. Different prevention and control strategies of primary care institutions would have different hysteresis effects on the evolution of epidemic transmission and the impact on primary healthcare services to varying degrees. The simulation results indicate that implementing screening and mask-wearing policies can reduce the infection rate of the population during the first week of an outbreak, with relatively minor impact on routine medical services for the primary healthcare institutions. Implementing screening and isolation policies can effectively reduce the infection rate of the population, with centralized isolation being more effective than home isolation during the early stages of an outbreak. Centralized isolation policies consume more medical resources of the primary care institutions, and as the number of isolation cases increases, their impact on routine medical services also intensifies. On the other hand, home isolation policies can reduce the demand for routine medical services to some extent while utilizing primary healthcare resources.

**Conclusion:** The agent-based model we developed is generalizable and can be utilized to forecast the impact of different emergency responses of primary care institutions on the disease spread and routine primary care services in the future, which can provide valuable insights for decision-makers. The results imply that it is necessary to balance resource utilization between emergency response and routine health service to improve population health. This study provides decision-making support for the development of optimal emergency responses for primary care institutions, as well as improved multidimensional prevention and control system.

**Funding:** The research was sponsored by the National Natural Science Foundation of China (72204156), Shanghai Pujiang Program (2020PJC081), Shanghai Jiao Tong University “Start-up Plan for New Young Teachers” (21X010501094), and Soft Science Project of Shanghai Science and Technology Innovation Action Plan (22692192000).

# Abstract: 17. Development of a Novel Ensemble Learning Algorithm-Based Model for Predicting Heart Failure Risk

**Authors:** Han Chen, Jiahao Min, Chenjie Xu

**Affiliations:** ^1^ School of Public Health, Hangzhou Normal University, Hangzhou, China.; ^2^ Hangzhou International Urbanology Research Center and Center for Urban Governance Studies, Hangzhou, China.

**Background:** The precise and punctual evaluation of the risk of heart failure is imperative for its early detection and prevention. However, the existing heart failure risk prediction models are limited to known risk factors and conventional statistical approaches.

**Objectives:** Our study aimed to utilize an ensemble learning algorithm to develop a novel heart failure prediction model in general population by combining nuclear magnetic resonance (NMR) metabolomic signatures with traditional risk factors of heart failure.

**Methods:** This study used a large UK population-based prospective cohort study conducted between 2006 and 2010, and a total of 117,272 participants who measured circulating metabolomic data by NMR technique were included. We created stratified random samples of the study population, dividing the dataset into training set (90% of data) and testing set (10% of data). A data-driven strategy was employed to identify predictors from 249 NMR metabolomic signatures and 329 traditional risk factors. Then, light gradient boosting machine (LightGBM) as an ensemble learning model was established to predict incident heart failure within 5 years, 10 years, and extended-years. The decision threshold was calibrated to determine the cutoff point that yielded the largest Youden index. Model performance was measured with the area under the receiver operating characteristic curve (AUROC) and average precision, and model calibration was assessed via calibration curves and Brier scores (Fig. 12).

**Fig. 12.** A graphical summary of the methods used in the study.

**Results:** During a median follow-up of 12.6 (interquartile range, 11.1 to 13.9) years, 3,547 participants developed heart failure. In the testing set, the ensemble learning models demonstrated superior performance in 5-year HF prediction [AUROC (95% CI) = 0.900 (0.872 to 0.924), average precision (95% CI) = 0.944 (0.904 to 0.964)] and exhibited high performance in both 10-year and all-incident heart failure prediction models [10-year heart failure: AUROC (95% CI) = 0.855 (0.830 to 0.876), average precision (95% CI) = 0.881 (0.843 to 0.903); all-incident heart failure: AUROC (95% CI) = 0.845 (0.825 to 0.864), average precision (95% CI) = 0.821 (0.778 to 0.850), respectively]. The most significant predictors across different time groups for the models were age, cardiovascular history, number of medications taken, cystatin C, waist circumference, vascular/heart problems diagnosed by doctor, forced expiratory volume in 1 s, red blood cell distribution width, HbA1c, income, father,s age at death, lymphocyte percentage, phospholipids to total lipids in very small VLDL percent, acetoacetate, and histidine. Final models for different incidence time groups exhibited excellent calibration (Brier score = 0.006, 0.018, and 0.027 for 5-year, 10-year, and all-incident heart failure, respectively). The model was validated internally in white ethnicity general population; however, external validation with independent datasets is warranted to corroborate these findings.

**Conclusion:** Our findings underscore the effectiveness of these machine learning-based models for predicting 5-year, 10-year, and much long years of incident heart failure. Furthermore, these models surpass conventional risk prediction methods by utilizing deep phenotyping of numerous metabolomic signatures, indicating their potential for practical application in early detection, prevention, and targeted intervention strategies for heart failure management.

**Funding:** This work was supported by the National Natural Science Foundation of China (grant number 72204071), Zhejiang Provincial Natural Science Foundation of China (grant number LY23G030005), and Scientific Research Foundation for Scholars of HZNU (grant number 4265C50221204119).

**Author contributions:** H.C., J.M., and C.X. conceived and designed the study. H.C. performed statistical analyses. H.C. drafted the manuscript. J.M. and C.X. supervised the study. All authors aided in the acquisition and interpretation of data, and critical revision of the manuscript. C.X. had access to and verified all of the data in the study.

# Abstract: 18. DxGPT: A Framework for Improved Medical Diagnosis Using Large Language Models and Machine Learning

**Authors:** Fei Wang, Qing Li, Pengfei Li

**Affiliations:** ^1^ Advanced Institute of Information Technology, Peking University, Hangzhou 311215, China.; ^2^ National Institute of Health Data Science, Peking University, Beijing 100191, China.; ^3^ Institute of Reproductive and Child Health/National Health Commission Key Laboratory of Reproductive Health and Department of Epidemiology and Biostatistics, School of Public Health, Peking University Health Science Centre, Beijing 100191, China.; ^*^ Address correspondence to: blacknepia@dingtalk.com

**Background:** Currently, numerous machine learning models have been used in the medical field because medical diagnosis requires a comprehensive understanding of patient,s symptoms, medical history, and test results. With the growing complexity of medical tasks that involve multiple modalities and domains, developing a universal interface between humans and machines has become increasingly crucial.

Large language models have demonstrated remarkable performance in natural language understanding and generation, and have been regarded as a potentially valuable tool for medical diagnosis. However, integrating these language models with medical machine learning models still poses challenges.

**Objectives:** In this paper, we propose DxGPT (Fig. 13), a framework that leverages large language models to manage and connect various medical machine learning models, thereby improving the accuracy and efficiency of medical diagnosis using natural language as a generic interface. The objective of the framework is to handle complex medical tasks involving multiple modalities and domains and achieve impressive results in medical diagnosis.

**Fig. 13.** Overview of DxGPT.

**Methods:** DxGPT is a framework that connects large language models and medical machine learning models to improve medical diagnosis accuracy and efficiency. The framework consists of four main steps: task planning, model selection, model execution, and result retrieval.

*Task Planning:* The first step in DxGPT is task planning. The large language model breaks down the user,s request into structured tasks requiring multiple machine learning models. The structured tasks define the information required from the user to proceed with the diagnosis process. This step involves understanding the user,s request, extracting relevant information.

*Model Selection:* The second step in DxGPT is model selection. The framework selects the most appropriate machine learning model based on the structured tasks defined in the previous step. The model selection is based on various factors such as the modality of the input and the domain of the problem. The selection of the model is critical to achieving accurate and efficient results.

*Model Execution:* The third step in DxGPT is model execution. The medical diagnosis machine learning model is called and run with the corresponding parameters based on the selected model in the previous step. The input parameters are generated by the large language model, and the output is generated by the machine learning model. This step involves interfacing with the medical machine learning models, which can be complex and require specific input parameters.

*Result Retrieval:* The fourth and final step in DxGPT is result retrieval. The results generated by the medical machine learning models are returned to the large language model, which decides whether to call further machine learning models or return the results to the user. The large language model processes the results and determines if additional information is required or if the diagnosis is complete. The result retrieval is a critical step as it determines the accuracy of the diagnosis and the effectiveness of the framework.

**Results:** Preliminary results show that DxGPT has the potential to improve diagnostic accuracy and clinical outcomes in real-world settings by assisting clinicians in making more accurate diagnoses. DxGPT can also reduce the time and cost associated with traditional diagnostic methods, making healthcare more efficient and cost-effective.

**Conclusion**: DxGPT is an innovative framework that connects large language models and medical machine learning models to improve medical diagnosis accuracy and efficiency. The framework can handle complex medical tasks involving multiple modalities and domains and achieve impressive results in medical diagnosis. With the increasing importance of developing a universal interface between humans and machines, DxGPT paves the way toward advanced artificial intelligence in the medical field.

**Funding:** This work was supported by the National Key Research and Development Program of China (2022YFF1203000)

# Abstract: 19. Efficiently Controlling for Sample Relatedness in a Large-Scale Genome-Wide Longitudinal Data Analysis and Its Application to UK Biobank

**Authors:** He Xu, Wenjian Bi

**Affiliations:** Department of Medical Genetics, School of Basic Medical Sciences, Peking University, Beijing 100191, China.

**Background:** As biobank data containing in-depth genetic data and extensively collected health information emerge across the world, complex phenotypes are gaining more attention in large-scale genome-wide association studies (GWAS). Longitudinal data can characterize the evolution of an individual’s health status or disease progression, and thus convey more information compared to regular cross-sectional phenotypes. Recently, TrajGWAS approach was proposed to identify genetic variants associated with longitudinal trajectories. Besides the mean-level trajectory patterns, TrajGWAS can also identify within-subject (WS) variability, which is also an important risk factor for some diseases. Simulation studies and real data analyses demonstrated that TrajGWAS can control type I error rates while remaining powerful for both common and low-frequency variants. Although well-developed, TrajGWAS is only applicable to analyze unrelated subjects, which could greatly reduce the sample size and thus lose statistical powers in a large-scale biobank data analysis.

Sample relatedness is a major confounder in GWAS and can result in inflated type I error rates if not appropriately controlled. Numeric mixed model approaches have been proposed to control for sample relatedness in a wide range of phenotypes including quantitative traits, binary traits, time-to-event data, and ordinal categorical data. Although previous methods have provided some insights, the utilization of this strategy remains technically challenging when analyzing more complex phenotypes. This includes difficulties in accurately estimating variance component, managing computational resources, conducting statistical tests, and addressing potential issues related to unbalanced phenotypic distribution, which greatly limit the application of GWAS to complex phenotypes such as longitudinal data.

**Objectives:** To overcome the aforementioned challenges, we propose SPA_GRM_, a scalable and accurate general-purpose approach designed for large-scale GWAS of complex phenotypes. We demonstrated SPA_GRM_ by conducting longitudinal data analysis in terms of simulation studies and real data analyses of UK Biobank.

**Methods:** SPA_GRM_ approach contains two main steps: In step 1, we fit a null model without considering the sample relatedness. Hence, many existing methods and well-developed tools can be used for this purpose. Here, in longitudinal data analysis, we follow TrajGWAS to fit a linear mixed model (LMM) framework and then calculate two sets of model residuals corresponding to the testing of mean and WS variability. In step 2, we consider genotype as random variable, use a pre-calculated GRM to adjust for the between-subject genetic correlation, and then utilize a hybrid strategy that combines normal distribution approximation and saddlepoint approximation to approximate the distribution of score statistics for testing. Our method is computationally efficient while remaining highly accurate for a wide range of genotypic distribution (common variants and rare variants) and phenotypic distribution (balanced and unbalanced distribution) in large-scale GWAS.

**Results:** We conducted extensive simulation studies to evaluate the performance of SPA_GRM_ in terms of type I error rates and powers for longitudinal trait analysis. Type I error rates are presented in Fig. 14. The results show that only SPA_GRM_ well controls for type I error rates in all scenarios, even for ultra-rare variants and familial aggregations. The power simulation and real data analysis indicated that SPA_GRM_ outperforms TrajGWAS in achieving higher power while well controlling type I errors for testing *β_g_* = 0 and *τ_g_* = 0 across all simulation scenarios (data not shown).

**Fig. 14.** Empirical type I error rates of SPA_GRM_, SPA_GRM(adj)_, Norm_GRM_, Norm_GRM(adj)_, and TrajGWAS methods. Top and bottom plots are for empirical type I error rates for testing *β_g_* = 0 and *τ_g_* = 0, respectively. From left to right, the plots considered three population structures of (A) small family-based population, (B) large family-based population, and (C) unrelated population. We compare the empirical type I error rates at significance level of 5 × 10^−7^ and 5 × 10^−5^.

**Conclusion:** We proposed a unified analysis framework SPA_GRM_ and demonstrated that SPA_GRM_ can efficiently analyze large datasets with hundreds of thousands of genetic related samples, can control type I error rates at a stringent significance level, and is statistically powerful. The framework was applied to analyze longitudinal data in this paper and is of high potential to expand to other complex phenotypes.

# Abstract: 20. Enhancing Health Data Exchange with Blockchain: Aligning with the Trusted Exchange Framework and Common Agreements

**Authors:** Yan Zhuang, Luxia Zhang

**Affiliations:** ^1^ National Institute of Health Data Science, Peking University, Beijing, China.; ^2^ Institute of Medical Technology, Health Science Center of Peking University, Beijing, China.; * Address correspondence to: zhanglx@bjmu.edu.cn

**Background:** The Trusted Exchange Framework and Common Agreement (TEFCA) is a regulatory framework designed to promote nationwide health information exchange (HIE) and improve interoperability among healthcare providers [1]. Blockchain technology, with its decentralized nature, immutability, and transparency, has the potential to enhance HIE and align with TEFCA,s principles. In this paper, we propose a blockchain-based system for HIE that adheres to the seven principles of TEFCA and addresses the limitations and challenges of current HIE solutions [2].

**Objectives:** The primary objective of this study is to demonstrate the feasibility of a blockchain-based system for HIE that meets the requirements of TEFCA,s seven principles, aiming to improve healthcare interoperability, promote patient-centered care, and ensure data privacy and security.

**Methods:** We designed a blockchain architecture, utilizing the Quorum blockchain, which is a variant of Ethereum, to implement smart contracts that aligns TEFCA’s principles, as listed in Table 1. The system involves multiple blockchain nodes representing hospitals, healthcare facilities, patients, and authorities. The architecture incorporates patient tokens with self-sovereign identity (SSI) mechanisms to enable patient-centered HIE. Additionally, we integrated dispute management smart contracts to ensure transparency and fairness in dispute resolution processes.

**Table 1.** Seven principles of TEFCA and blockchain solutions

TEFCA principle	Blockchain-based HIE implementation
Standardization	Utilizes Quorum blockchain and Ethereum-based smart contracts to standardize HIE processes.
Openness and transparency	Ensures transparency through the immutable and public nature of the blockchain, as well as through dispute management smart contracts.
Cooperation and non-discrimination	Promotes cooperation among healthcare providers by providing an efficient, transparent, and secure HIE platform.
Privacy, security, and safety	Incorporates SSI mechanisms and patient tokens to protect patient privacy and ensure data security.
Access	Empowers patients to control their health data, granting or revoking access permissions to healthcare providers as needed.
Equity	The decentralized nature of the blockchain ensures data-driven accountability by allowing all stakeholders to access the transaction history.
Public health	Facilitates public health surveillance and supports biomedical research by providing a transparent, secure, and standardized platform for data exchange.

**Results:** The proposed blockchain-based system for HIE effectively addresses the TEFCA principles, providing a transparent, secure, and decentralized platform for data sharing. The patient tokens with SSI mechanisms empower patients to have full control over their health data, allowing them to grant or revoke access permissions to healthcare providers. Our implementation demonstrates that blockchain can facilitate efficient, transparent, and secure HIE while reducing the risk of disputes and promoting cooperation and non-discrimination. Furthermore, blockchain technology has the potential to enhance public health surveillance, support biomedical research, and promote health equity.

**Conclusion:** Blockchain technology presents a promising solution to achieve the goals of TEFCA and improve healthcare interoperability. By leveraging the unique features of blockchain, the proposed system enables secure, transparent, and patient-centered HIE that adheres to TEFCA,s principles. Although limitations and challenges, such as scalability and adoption costs, need further exploration, the potential benefits of blockchain in healthcare are significant. Future research directions include integrating blockchain with emerging technologies, such as machine learning and artificial intelligence, to create a learning health system, and collaborating with health information networks to fully evaluate the feasibility and efficiency of blockchain-based HIE mechanisms.

**Acknowledgments:** This research was supported in part by grant 2022YFF1203000 from the National Key R&D Program of China, grant 72125009 from the National Natural Science Foundation of China, and 2020BD004 from PKU-Baidu Fund.

## References

[1] The Trusted Exchange Framework (TEF): Principles for Trusted Exchange. 2022. https://www.healthit.gov/sites/default/files/page/2022-01/Trusted_Exchange_Framework_0122.pdf.

[2] Zhuang Y, Sheets LR, Chen YW, Shae ZY, Tsai JJP, Shyu CR. A patient-centric health information exchange framework using blockchain technology. IEEE J Biomed Health Inform. 2020;24(8):2169–2176.32396110 10.1109/JBHI.2020.2993072

# Abstract: 21. Enhancing Patient Privacy in Health Information Exchange through Self-Sovereign Identity and Non-Fungible Tokens

**Authors:** Yan Zhuang, Luxia Zhang

**Affiliations:** ^1^ National Institute of Health Data Science, Peking University, Beijing, China.; ^2^ Institute of Medical Technology, Health Science Center of Peking University, Beijing, China.; ^*^ Address correspondence to: zhanglx@bjmu.edu.cn

**Background:** Patient privacy and confidentiality are crucial for trust-building between patients and healthcare providers. Regulations like HIPAA aim to protect patients, protected health information (PHI) from unauthorized access or breaches. However, data breaches highlight the urgent need for secure medical history sharing methods. Current patient tokenization methods, managed by third parties, inadequately address security concerns. Blockchain technology, with its decentralized nature, provides a potential solution for enhancing privacy and control in health information exchanges (HIEs) [1].

**Objectives:** This study aims to develop a self-sovereign identity (SSI)-enabled patient tokenization system using non-fungible tokens (NFTs) and blockchain technology [2]. The system intends to enhance patient privacy and control in HIEs while maintaining transparency and trust, addressing the limitations of existing tokenization methods.

**Methods:** We designed an architecture consisting of four modules, as shown in Fig. 15: (a) Creation Module, generating NFTs through blockchain-based smart contracts, and storing biometric data locally with hashes inside NFTs for consistency and proof of ownership; (b) Linkage Module, connecting patient accounts to NFTs for tokenization; (c) Authentication Module, validating patient identities and ensuring the validity of identities; and (d) Exchange Module, verifying NFT and facilitating HIE processes.

**Fig. 15.** Four modules contained in the overall architecture for the use of NFT: NFT creation, ID linkage, user authentication, and data exchange.

We implemented the system using the Quorum blockchain. To test the feasibility of the proposed system in a practical scenario, we conducted a case study involving a patient with chronic kidney disease who needed to relocate to a new city. The patient did not want their new healthcare providers to know about their history of alcohol addiction prior to the diagnosis of chronic kidney disease. We created and ran a script containing the following steps every second continuously for a week: (a) the patient granting permission to a doctor, (b) the doctor sending requests to the remote healthcare facility, and (c) the remote healthcare facilities sending the IPFS hashes to the doctor. This case study aimed to assess the system,s ability to securely handle sensitive patient information and provide a seamless HIE experience while maintaining patient privacy and control.

**Results:** The case study and stability tests provided valuable insights into the performance and robustness of the proposed SSI-enabled patient tokenization system. The results demonstrated the system,s feasibility, efficiency, and robustness in handling HIE processes while ensuring patient privacy and control. The case study reported a 100% success rate in processing over three million transactions. The average blockchain validation and writing time was 1.17 s, and the stability test revealed an average processing time of 1.42 s when handling 200 transactions per second for an hour.

**Conclusion:** The proposed system successfully utilizes blockchain technology and NFTs to provide patients with increased control and privacy in HIEs, addressing the limitations of existing tokenization methods. Future work will address scalability, explore NFT potential in data exchanges, and develop comprehensive HIE functionalities based on NFT authentication. Large-scale simulations using real-world data will be conducted to evaluate the system,s feasibility, stability, scalability, and security, ensuring a reliable solution for secure HIE.

**Acknowledgments:** This research was supported in part by grant 2022YFF1203000 from the National Key R&D Program of China, grant 72125009 from the National Natural Science Foundation of China, and 2020BD004 from PKU-Baidu Fund.

## References

[1] Mackey TK, Calac AJ, Chenna Keshava BS, Yracheta J, Tsosie KS, Fox K. Establishing a blockchain-enabled Indigenous data sovereignty framework for genomic data. Cell. 2022;185(15):2626–2631.35868267 10.1016/j.cell.2022.06.030

[2] Zhuang Y, Shyu C-R, Hong S, Li P, Zhang L. Self-sovereign identity empowered non-fungible patient tokenization for health information exchange using blockchain technology. Comput Biol Med. 2023;157: Article 106778.36934533 10.1016/j.compbiomed.2023.106778

# Abstract: 22. External Validation of the Kidney Failure Risk Equations among Urban Community-Based Chinese Patients with Chronic Kidney Disease

**Authors:** Jinwei Wang, Ling Pan, Yang Deng, Luxia Zhang

**Affiliations:** ^1^ Renal Division, Department of Medicine, Peking University First Hospital, Beijing, China.; ^2^ Renal Division, First Affiliated Hospital of Guangxi Medical University, Nanning, China.; ^3^ National Institute of Health Data Science at Peking University, Beijing, China.

**Background:** The kidney failure risk equations have been proven to perform well in multinational databases, while they lack validation studies in the Chinese population.

**Objectives:** This study sought to externally validate the equations in a community-based chronic kidney disease cohort.

**Methods:** Patients with estimated glomerular filtration rate of <60 ml/min/1.73 m^2^ dwelling in an industrialized coastal city of China were enrolled in this retrospective cohort study. Predictors of the equations included age, sex, estimated glomerular filtration rate, and albuminuria in the 4-variable model while adding serum calcium, phosphate, bicarbonate, and albumin in the 8-variable model. The outcome was initiation of chronic dialysis treatment. Model discrimination, calibration, and clinical utility were evaluated by Harrell’s C-statistic, calibration plots, and decision curve analysis, respectively.

**Results:** A total of 4,587 participants were enrolled for the validation of the 4-variable model, while 1,414 participants were enrolled for the 8-variable one. The median time of follow-up was 5.60 ± 3.00 years for the 4-variable model and 4.15 ± 0.95 years for the 8-variable one. For the 4-variable model, the C-statistics were 0.750 (95% confidence interval: 0.615 to 0.885) for the 2-year model and 0.766 (0.625 to 0.907) for the 5-year one. A slight improvement was observed for the 8-variable models [the C-statistics for the 2-year and 5-year models were 0.756 (0.629 to 0.883) and 0.774 (0.641 to 0.907), respectively]. (Table 1) By assessing the calibration plots visually, calibration was acceptable for both the 8-variable model and the 4-variable one. Decision curve analysis showed that both the 4-variable and the 8-variable 5-year models performed better throughout different net benefit thresholds than the estimated glomerular filtration rate-based strategy of estimated glomerular filtration rate <30 ml/min/1.73 m^2^.

**Table 1.** Harrell,s C-statistics, category-free net reclassification index, and integrated discrimination index of applying kidney failure risk equations in the current cohorts

Models	Harrell,s C-statistics (95% confidence interval)	Net reclassification index (95% confidence interval)	Integrated discrimination index (95% confidence interval)
2-year 4-variable model	0.750 (0.615, 0.885)	NA	NA
2-year 8-variable model	0.766 (0.625, 0.907)	0.002 (−0.137, 0.116) ^a^	0.279 (−0.156, 0.622) ^a^
5-year 4-variable model	0.756 (0.629, 0.883)	NA	NA
5-year 8-variable model	0.774 (0.641, 0.907)	−0.001 (−0.129, 0.116) ^a^	0.136 (−0.251, 0.546) ^a^

^a^ The net reclassification index or integrated discrimination index comparing 2-year/5-year 8-variable model to 2-year/5-year 4-variable model was provided based on the 1,414 patients with complete data of the 8-variable model.

**Conclusion:** The kidney failure risk equations showed good discrimination, acceptable calibration, and better clinical utility than the estimated glomerular filtration rate-based strategy for incidence of kidney failure among community-based urban Chinese patients with chronic kidney disease.

# Abstract: 23. Extraction and Analysis of Positive and Negative Results Reported at Clinicaltrials.gov

**Authors:** Xuanyu Shi, Zitao Liang, Jian Du

**Affiliations:** ^1^ National Institute of Health Data Science, Peking University, Beijing, China.; ^2^ Institute of Medical Technology, Peking University, Beijing, China.; * Address correspondence to: dujian@bjmu.edu.cn

**Background:** The realm of scientific failures has been an underexplored area, especially common in clinical trials. At the top of the pyramid of evidence-based medicine, randomized controlled trials (RCTs) output the most direct and reliable evidence for medical decision making. The Clinicaltrials.gov platform stores an extensive collection of global registered studies including reported results with statistical analyses, as well as valuable metadata, including funding sources, intervention types, enrollment numbers, and more.

**Objectives:** The aim of this study is to develop a methodological pipeline for the classification of positive and negative outcomes derived from reported statistical analyses. Additionally, we intend to conduct data-driven investigations based on the attributes of these studies in order to discover the factors that contribute to the emergence of positive and negative results.

**Methods:** We downloaded all the studies with statistical analysis from clinicaltrials.gov. Intervention–outcome pairs and metadata were extracted from the XML file and curated as study results. Each result was defined as positive or negative by applying a rule-based model utilizing corresponding *P* values and confidence intervals. Annual trends in proportions were examined to show the general landscape and potential associations between the significance of results with funding type, intervention type, number of enrollments, and outcome type.

**Results:** We extracted 43.61% positive and 56.39% negative results from 18,720 registered studies with 222,361 statistical analysis records (intervention–outcome pairs) on clinicaltrials.gov. Corresponding characteristics of the studies were analyzed and visualized. In general, the ratio of negative results in non-industry-funded studies exceeded that in industry-funded studies (66.2% versus 53.8%, and 63.4% in partially-industry). Among most of the intervention types, behavioral interventions exhibited the highest percentage of negative outcomes (68.1%). Results with enrollment numbers fewer than 50 tended to yield more negative results (60.4%) in comparison to those with more than 50 participants (56.1%). By employing medical subject headings (MeSH), we examined a small subset of biomarker-oriented outcomes, identifying a 67.4% ratio of negative results (Fig. 16).

**Fig. 16.** An overview of the study workflow.

**Conclusion:** Our study developed a methodological pipeline to classify positive and negative outcomes from clinical trials registered on Clinicaltrials.gov. We identified factors contributing to these outcomes, discovering a higher ratio of negative results with non-industry fundings, behavioral interventions, and biomarker-oriented outcomes. Besides, studies with under 50 participants also tended to have more negative results. These findings provide valuable insights for researchers and stakeholders in evidence-based medicine.

# Abstract: 24. Factors that Influence Translational Medical Research Process Performance of Doctors: Based on Structural Equation Modeling (SEM)

**Background:** Translational medical research (TMR) attached great importance worldwide. The relationship between the characteristic of TMR, the behavior of TMR, and physicians’ TMR process performance has yet to be sufficiently validated.

**Objectives:** This study aimed to identify the factors that influence TMR process performance. In addition, we explored its path and mechanisms that reveal how these factors affect it.

**Methods:** We qualitatively analyzed TMR’s collaborative research process and divided it into three levels: characteristic, behavior, and performance. Next, a conceptual model of collaborative research performance was constructed by analyzing the levels and relationships of collaborative TMR research. Finally, we proposed research hypotheses based on this model.

The factors influencing physicians’ TMR process performance were investigated with a questionnaire using a 5-point Likert scale. It was administered via an online survey from 2020 July 29 to 2020 October 12. We applied structural equation models to fit survey data. In addition, we established corresponding measurement and structural models.

We employed exploratory factor analysis (EFA) to test the questionnaire’s reliability and validity. Cronbach’s alpha was used to measure the variables’ internal consistency level. In addition, orthogonal rotation was performed via the maximum variance method using factors with eigenvalues ≥1. Finally, an item factor loading of >0.5 was chosen to ensure that the questionnaire had good structural validity.

Based on survey reliability and validity tests, AMOS 17.0 was used to perform structural equation modeling (SEM). This method enabled the definition of measured versus latent variables and their relationship.

**Results:** We received 446 valid questionnaires. The reliability and validity of the questionnaire were confirmed. Cronbach’s alpha value is 0.956. The discriminant validity results showed that the square root of the average variance extraction (AVE) is larger than the non-diagonal elements of the corresponding rows and columns; this indicates that the scale has good discriminant validity. The EFA results showed that the first factor’s variance accounted for over 40% of all factor variances. In addition, the cumulative variance contribution rate of the first seven factors (collaborative behavior and ability, resource sharing, collaborative risk, research process performance, TMR uncertainty, relationships and motivation, and TMR complexity) reached 70.388% (>70%). The seven common factors are strongly persuasive based on the data’s primary information.

The result of the chi-square test of goodness of fit (χ^2^/df) was 1.032, and its index (GFI) was 0.998. The adjusted goodness of fit index (AGFI) was 0.98. The normed fit index (NFI) was 0.99, the comparative fit index (CFI) was 1.000, and the root mean square error of approximation (RMSEA) value was 0.008. The results showed that the constructed model fits the data well.

The results show that seven hypotheses out of 12 were supported (*P* < 0.05) (Table 1). TMR research uncertainty does not significantly impact resource sharing, collaborative behavior and ability, collaborative relationships, and motivation. In contrast, research resource sharing, collaborative relationships, and TMR motivation do not significantly impact research performance. TMR complexity positively impacts resource sharing, collaborative relationships and motivation, and collaborative behavior and ability. TMR’s collaborative risk positively impacts collaborative behavior and ability, collaborative relationships and motivation, and resource sharing. Finally, TMR collaboration behavior and ability positively impact research process performance.

**Table 1.** Results of hypotheses testing. ***P < 0.001.

Hypothesis	Path relationship	Estimate value	S.E.	C.R.	*P*	Hypothesisdecision
H1-1:X2←X5	Resource sharing←TMR uncertainty	−0.019	0.064	−0.294	0.769	Not supported
H1-2:X2←X7	Resource sharing←TMR complexity	0.432	0.068	6.319	***	Supported
H1-3:X6←X7	Collaborative relationships and motivation←TMR complexity	0.172	0.037	4.628	***	Supported
H1-4:X1←X3	Collaborative behavior and ability←Collaborative risk	0.561	0.140	4.015	***	Supported
H1-5:X6←X3	Relationships and motivation←Collaborative risk	0.126	0.030	4.244	***	Supported
H1-6:X1←X7	Collaborative behavior and ability←TMR complexity	1.244	0.175	7.119	***	Supported
H1-7:X6←X5	Relationships and motivation←TMR uncertainty	0.016	0.035	0.458	0.647	Not Supported
H1-8:X1←X5	Collaborative behavior and ability←TMR uncertainty	−0.294	0.163	−1.804	0.071	Not supported
H1-9:X2←X3	Resource sharing←Collaborative risk	0.167	0.055	3.058	0.002	Supported
H2-1:X4←X1	TMR process performance←Collaborative behavior and ability	0.144	0.014	10.546	***	Supported
H2-2:X4←X2	TMR process performance←Resource sharing	0.026	0.036	0.718	0.473	Not Supported
H2-3:X4←X6	TMR process performance←Relationships and motivation	0.008	0.060	0.136	0.892	Not supported

**Conclusion:** Our findings enhance the current understanding of how characteristics and behavior affect TMR process performance. Furthermore, findings suggest that facilitating collaborative behavior and improving collaborative ability are essential for successful translational research process performance.

# Abstract: 25. From Knowledge Graph to Directed Acyclic Graph (KG2DAG): A New Opportunity for Literature-Based Discovery

**Authors:** Yongmei Bai, Jian Du

**Affiliations:** ^1^ Institute of Medical Technology, Peking University Health Science Center, Beijing, China.; ^2^ National Institute of Health Data Science, Peking University, Beijing, China.; * Address correspondence to: dujian@bjmu.edu.cn

**Background:** In order for observational research to establish causality instead of just correlation, causal modeling is necessary. A useful tool for guiding causal modeling is the directed acyclic graph (DAG). However, there are limitations to using DAGs. First, it requires manual-readable literature and expert experience, which is not always feasible given the increasing growth of scientific literature. Second, in DAGs, there are three types of relations linking the investigated exposure-outcome variables, i.e., common cause (cofounder), common effect (collider), as well as intermediates. Constructing such a full knowledge network is challenging for humans. To address these limitations, we propose a method called knowledge graph to directed acyclic graph (KG2DAG). The third-party variables linking COVID-19 exposure and acute kidney failure (AKI) outcome were constructed and validated.

**Objectives:** We proposed a systematic approach for constructing DAGs from KG. The research questions are as follows: (a) Constructing a KG for cause-and-effect clinical knowledge for COVID-19, (b) how can we systematically find the third-party variables from the KG? (c) How can the path between exposure and outcome be systematically queried and validated?

**Methods:** Various database platforms were used to search for knowledge related to COVID-19. We extracted semantic triples using SemMedDB and SemRep. The standardization and vocabulary mapping of entities were conducted using the unified medical language system (UMLS) and OHDSI Usagi. The knowledge from UMLS API was imported into the GraphDB database for Web Ontology Language to Rule Language (OWL 2 RL) reasoning. Neo4j was used for KG construction and DAG query. The research also evaluated the evidence grade of each triple. The criteria of 80 clinical trials that have condition about “COVID-19” and the outcome of “acute kidney failure (AKI)” were used for our validation (Fig. 17).

**Fig. 17.** Flow chart of triple screening for COVID-19 clinical knowledge.

**Results:** A KG includes 6,772 nodes, and 23,964 relations were constructed using structured semantic triples extracted from biomedical literature and descriptive text in registered clinical trials and granted research projects. A query case study was conducted using “COVID-19” and “AKI.” We performed a path query between the two variables. We got the third-party variables between the exposure (COVID-19) and the outcome (AKI). It was illustrated that length of hospital stay (Duration), chemical agents (Lopinavir + ritonavir, Antiviral Agents, tetrandrine, Rintatolimod, nafamostat), pathological process (Single organ dysfunction, Respiratory distress, Respiratory Distress Syndrome, Adult), medical procedures (Coagulation procedure, Plasma Exchange, Renal Replacement Therapy, Prolonged Intermittent Renal Replacement Therapy, Continuous Renal Replacement Therapy), comorbidities (Hypertensive disease, Diabetes Mellitus), and gene, protein, and metabolite (CD69 protein, human|CD69, GJA1 gene|GJA1, Angiopoietin-2, PLAUR protein, human|PLAUR, PTX3 protein, Fibrin fragment D, C-reactive protein) are identified as confounders. Acute respiratory distress syndrome (ARDS) is the main lung disease in COVID-19 patients, and it becomes a confounder/mediator/collider for COVID-19 exposure and AKI outcome. In addition to ARDS, poor prognosis (Prognosis bad and Death in hospital) was identified as a collider. These covariates will inform our data analysis and interpretation.

**Conclusion:** Through our knowledge discovery approach, we can evaluate the evidence-level and identify mechanisms through which interventions and exposures may influence health outcomes. A DAG related to a certain disease can be generated by extracting entities and triple information from free texts on multiple platforms, which can be used by clinical experts for path retrieval and variable queries. This result indicated that during the design of clinical studies, biological differences may exist. These factors are not easy to observe but may affect the test results. The DAG we built gives rich attributes of entities and relationships, including the time of knowledge generated. But there is still some limitation in this study. In the future, we will try to use covariate adjustment and sequencing studies with real-world case or cohort data.

# Abstract: 26. How to Report or Assess the Study with the Application of Artificial Intelligence or Big Data to Healthcare

**Authors:** Renfeng Su, Yaolong Chen

**Affiliations:** ^1^ School of Public Health, Lanzhou University, Lanzhou, China.; ^2^ Research Unit of Evidence-based Evaluation and Guidelines, Chinese Academy of Medical Sciences, School of Basic Medical Sciences, Lanzhou University, Lanzhou, China.; ^3^ Lanzhou University Institute of Health Data Science, Lanzhou, China.; * Address correspondence to: chevidence@lzu.edu.cn

**Background:** These applications of artificial intelligence (AI) or big data are increasing in the medical field. It is necessary to use quality assessment tools such as reporting and methodological quality tools to evaluate the quality of research, and ensure transparency and quality.

**Objectives:** This study aims to identify and report on tools related to methodology quality and reporting quality on AI and big data.

**Methods:** We searched for potentially relevant articles in PubMed, Embase, Web of Science, EQUATOR, CNKI, Wanfang Data, and CBM, covering the period from inception to March 2023.

**Results:** We identified and included a total of 21 research articles. Over 90% of the studies, first author institutions were universities, followed by associations/academies (one, 4.7%) and research institutes (one, 4.7%). In terms of journals where the studies were published, 10 studies were published in journals with impact factor ≤10 (47.5%), and 8 studies were published in journals with impact factor >10 (38.1%). We revealed 22 types of tools, and the most common types of studies were checklists (1, 47.6%), guidelines (1, 28.5%), standards (2, 9.5%), tools (1, 4.7%), frameworks (1, 4.7%), and recommendations (1, 4.7%). Thirteen tools (59.1%) focused on reporting guidelines, 5 tools (22.7%) focused on methodology assessment tools, and the remaining tools (4, 18.2%) focused on both. The Delphi method and literature review were the most commonly used techniques for tool development across the studies.

**Conclusion:** Our findings may have important implications for the development of future evaluation tools in this scientific domain.

# Abstract: 27. Identifying Subgroups of ICU Patients Using End-to-End Multivariate Time-Series Clustering Algorithm Based on Real-World Vital Signs Data

**Authors:** Tongyue Shi, Zhilong Zhang, Wentie Liu, Junhua Fang, Jianguo Hao, Shuai Jin, Huiying Zhao, Guilan Kong

**Affiliations:** ^1^ National Institute of Health Data Science, Peking University, Beijing, China.; ^2^ School of Computer Science and Technology, Soochow University, Suzhou, Jiangsu, China.; ^3^ Peking University People’s Hospital, Beijing, China.; ^4^ Advanced Institute of Information Technology, Peking University, Hangzhou, Zhejiang, China.; * Address correspondence to: guilan.kong@hsc.pku.edu.cn

This study employed the Medical Information Mart for Intensive Care IV (MIMIC-IV) database as a data source to investigate the use of dynamic, high-frequency, multivariate time-series vital signs data, including temperature, heart rate, mean blood pressure, respiratory rate, and SpO_2_, monitored during the first 8 h in the intensive care unit (ICU). Various clustering algorithms were compared, and an end-to-end multivariate time series clustering system called Time2Feat, combined with *k*-means, was chosen as the most effective method to cluster patients in the ICU. In clustering analysis, data of 8,080 patients admitted between 2008 and 2016 were used for model development and data of 2,038 patients admitted between 2017 and 2019 were used for model validation. By analyzing the differences in clinical mortality prognosis among different categories, varying risks of ICU mortality and hospital mortality were found between different subgroups. Furthermore, the study visualized the trajectory of vital sign changes. The findings of this study provide valuable insights into the potential use of multivariate time-series clustering systems in patient management and monitoring in the ICU setting.

**Introduction:** The ICU is a specialized medical facility that provides intensive monitoring and treatment for critically ill patients. ICU patients are characterized by severe illness and life-threatening conditions, requiring close monitoring and treatment. The changes in vital signs have multifaceted implications for patients. Existing research on patient subgroup analysis often focuses on single diseases and depends on cross-sectional analysis [1], and the value of dynamic multivariate time-series data of vital signs has not been utilized [2]. Therefore, there is a research gap in the literature about making full use of time-series vital sign data to explore subgroups in ICU patients for precision ICU care.

**Objectives:** This study aimed to use end-to-end multivariate time-series clustering algorithm to identify subgroups of ICU patients based on dynamic vital sign data recorded during the first 8 h after ICU admission, and then to further explore the differences of prognoses in different patient subgroups. Overall, this study would make contributions to precision ICU care by classifying patients into different subgroups for more personalized clinical interventions.

**Methods:** In this study, the MIMIC-IV database [3] was used as the data source. The dynamic, high-frequency vital sign data monitored in ICU during the first 8 h were used for analysis. We used multivariate time-series clustering algorithms to cluster and group critically ill ICU patients first and then analyzed the patient prognosis in different subgroups to help clinicians identify those patients with high mortality risk. All adult ICU patients in MIMIC-IV were included. In clustering analysis, data of patients admitted between 2008 and 2016 were used for model development and data of patients admitted between 2017 and 2019 were used for model validation. Variables including gender, age, race, height, weight, and date of death (DoD), together with hourly monitored vital signs (temperature, heart rate, mean blood pressure, respiratory rate, and SpO_2_), were extracted. For patients having multiple ICU stays in one hospital admission, only the first ICU admission record was extracted. Patients were excluded if they had missing values for the extracted variables. Patient prognoses including ICU mortality and hospital mortality were analyzed for each patient subgroup. Finally, the elbow method and metrics including Davies–Bouldin index (DBI) and Calinski–Harabaz index (CHI) were used to determine the optimal number of clusters (*k*) and optimal clustering algorithm [4].

**Results:** In this study, a total of 10,118 patients including 8,080 patients admitted between 2008 and 2016, and 2,038 patients admitted between 2017 and 2019 were included. Several clustering models, including Time2Feat [5] combined with *k*-means, *k*-shape, *k*-medoids, and density-based spatial clustering of applications with noise (DBSCAN), were employed for analysis, and finally, the Time2Feat combined with *k*-means model was selected as it had the best performance with CHI of 341.59 and DBI of 5.92. According to the elbow method, the optimal number of clusters is determined to be 3 (*k* = 3). In model development process, 8,080 patients admitted from 2008 to 2016 were divided into three subgroups. In model validation process, 2,038 patients admitted from 2017 to 2019 were divided into three subgroups as well. As depicted in Fig. 18, vital sign trajectories in the three identified subgroups are similar in both the model development and validation datasets. There are noticeable differences in the trajectories of heart rate, SpO_2_, temperature, and respiratory rate among the three subgroups, while the average blood pressure trajectories show less apparent distinctions in the three subgroups. As to hospital mortality, on the dataset for model development, the risks ranked from highest to lowest were subgroup2 (0.1092 ± 0.005), subgroup1 (0.0875 ± 0.0104), and subgroup3 (0.0867 ± 0.0048); on the validation dataset, the risks showed consistent order: subgroup2 (0.1245 ± 0.0117), subgroup1 (0.1218 ± 0.0145), and subgroup3 (0.1033 ± 0.0113). Regarding the ICU mortality, the risks ranked from highest to lowest were subgroup1 (0.0485 ± 0.0079), subgroup2 (0.0468 ± 0.0034), and subgroup3 (0.0242 ± 0.0026) on the model development dataset, and the order was subgroup2 (0.0436 ± 0.007), subgroup1 (0.0393 ± 0.0086), and subgroup3 (0.0234 ± 0.0056) on the validation dataset. There was a slight ICU mortality difference between subgroup1 and subgroup2. Considering the smaller sample size in the validation dataset, there is a certain margin of error. However, both subgroup1 and subgroup2 had higher ICU mortality rates than the overall rate (0.0353 ± 0.0041).

**Fig. 18.** Trajectories of vital signs during the first 8 h in the three identified ICU patient subgroups.

**Conclusion:** The multivariate time-series data of vital signs monitored during the first 8 h after ICU admission can reflect real conditions of patients and help to predict prognoses to some extent. By employing proper multivariate time-series clustering algorithm to make second use of real-world vital sign data recorded in ICU could help clinicians to identify distinct patient subgroups with different mortality risks. The Time2Feat combined with *k*-means method used in this study has shown satisfactory clustering performance. In the next step, we will generalize the time-series clustering approach to other diseases and refine the model in practical applications.

**Funding:** This study was supported by grants from the Zhejiang Provincial Natural Science Foundation of China (grant no. LZ22F020014), National Key Research and Development Program of China (grant no. 2018AAA0102100), Beijing Municipal Science & Technology Commission (grant no. 7212201), and Humanities and Social Science Project of Chinese Ministry of Education (grant no. 22YJA630036).

## References

[1] Liu K, Zhang X, Chen W, Yu ASL, Kellum JA, Matheny ME, Simpson SQ, Hu Y, Liu M. Development and validation of a personalized model with transfer learning for acute kidney injury risk estimation using electronic health records. JAMA Netw Open. 2022;5(7):e2219776.10.1001/jamanetworkopen.2022.19776PMC925005235796212

[2] Tharakan S, Nomoto K, Miyashita S, Ishikawa K. Body temperature correlates with mortality in COVID-19 patients. Crit Care. 2020;24:1–3.32503659 10.1186/s13054-020-03045-8PMC7274509

[3] Johnson AEW, Bulgarelli L, Shen L, Gayles A, Shammout A, Horng S, Pollard TJ, Hao S, Moody B, et al. MIMIC-IV, a freely accessible electronic health record dataset. Sci Data. 2023;10(1):1.36596836 10.1038/s41597-022-01899-xPMC9810617

[4] Bonifati A, Buono FD, Guerra F, Tiano D. Time2Feat: Learning interpretable representations for multivariate time series clustering. Proc VLDB Endowment. 2022;16(2):193–201.

[5] Kodinariya TM, Makwana PR. Review on determining number of cluster in k-means clustering. Int J. 2013;1(6):90–95.

# Abstract: 28. Innovative Collaboration between Hospitals and Biomedical Industry in China

**Authors:** Jiahe Tian, Xiang Liu, Hong Chen, Meina Li, Wenya Yu

**Affiliations:** ^1^ School of Public Health, Shanghai Jiao Tong University School of Medicine, Shanghai 200025, China.; ^2^ Department of Respiratory Disease, The 903rd Hospital of PLA, Hangzhou 310000, China.; ^3^ Department of Military Health Management, Second Military Medical University, Shanghai 200433, China.; * Address correspondence to: jsjyyuwenya@sina.cn; † These authors contributed equally to this work.

**Background:** With the healthcare development in China, hospitals have the need for innovative development in clinical research, diagnosis, treatment, and technology transformation, and the biomedical industry is entering a crucial period of equal emphasis on research and development, transformation, and manufacturing. Considering that the essence of innovative collaboration is innovation and transformation, innovative collaboration is recognized as the most important cornerstone to promote the development of both hospitals and the biomedical industry. However, it is not clear about the current situation, willingness, and cognition of innovative collaboration between Chinese hospitals and the biomedical industry, which fails to provide evidence for effective promotion of their innovative collaboration.

**Objectives:** In the perspective of hospitals and the key role of medical staff in the implementation of innovative collaboration, this study aimed to investigate the current situation, willingness, and cognition of innovative collaboration in China. It would help clarify the current characteristics and the potentials of innovative collaboration between Chinese hospitals and the biomedical industry, which would provide policy suggestions and directions for effective promotion of innovative collaboration in China.

**Methods:** A cross-sectional survey was conducted from October 2022 to April 2023. Participants were medical staff working in Chinese hospitals, including doctors, nurses, medical technicians, managers, and researchers. This investigation referred to 20 provinces in China, including 489 medical staff. The survey information included hospital characteristics, medical staff’s demographic characteristics, and innovative collaboration characteristics. Descriptive analysis, chi-square test, and logistic regression analysis were used for statistical analysis.

**Results:** Only 7.0% of participants had experience in innovative collaboration with the biomedical industry (Table 1). Among them, medical staff from tertiary hospitals (versus secondary hospitals), with a doctoral degree (versus master,s or bachelor,s degree), and with senior professional titles (versus intermediate or junior title) had significantly richer experience in innovative collaboration. The main innovative collaboration types included joint application for and implementation of research projects (58.8%), joint implementation of clinical trials (47.1%), and joint application for and implementation of research projects supported by biomedical companies (38.2%). The average number of innovative collaboration was 1.97 ± 1.57, and the main partner was a department or team of a biomedical company (61.8%). Medical staff acting as principal investigators in innovative collaboration relationships accounted for the least (23.5%). The main output derived from innovative collaboration included research publications (50.0%) and projects (35.3%). However, promisingly, regardless of whether having or not having experience in innovative collaboration with the biomedical industry previously, most medical staff were willing to engage in innovative collaboration with the biomedical industry in the future (86.3%). Univariate analysis showed that medical staff ,s willingness of innovative collaboration with the biomedical industry was affected by hospital level, educational level, and innovative collaboration experience and cognition (including the partner, personal role, and output of innovative collaboration). The logistic regression model further suggested that the key factor influencing medical staff’s willingness of innovative collaboration with the biomedical industry was their experience in or cognition of research publications derived from innovative collaboration. The willingness of innovative collaboration of medical staff who had experience in or expectations of research publications was 414 times stronger than those not having such experience or expectations.

**Table 1.** Medical staff,s experience and willingness of innovative collaboration with the biomedical industry

Characteristic	Estimate	*P* value	OR	95% Wald CI	
				Lower limit	Upper limit
Experience in innovative collaboration with the biomedical industry					
Hospital level					
Tertiary hospital	Ref	Ref	Ref	Ref	Ref
Secondary hospital	−2.476	0.018	0.084	0.011	0.653
Primary hospital	−12.817	0.983	<0.001	<0.001	>999.999
Sex					
Male	Ref	Ref	Ref	Ref	Ref
Female	−0.696	0.071	0.498	0.234	1.062
Educational level					
Doctoral degree	Ref	Ref	Ref	Ref	Ref
Master,s degree	−1.045	0.032	0.352	0.136	0.912
Bachelor,s degree	−1.204	0.009	0.3	0.121	0.744
Junior college,s degree or lower	−12.962	0.983	<0.001	<0.001	>999.999
Professional title					
Senior	Ref	Ref	Ref	Ref	Ref
Associate senior	−0.801	0.151	0.449	0.15	1.339
Intermediate	−1.144	0.022	0.319	0.12	0.847
Junior	−1.413	0.037	0.244	0.065	0.918
Willingness of innovative collaboration with the biomedical industry					
Whether having experience or expectations of research publications from innovative collaboration					
No	Ref	Ref	Ref	Ref	Ref
Yes	6.026	<0.0001	414.206	56.388	>999.999

**Conclusion:** The current level of innovative collaboration between hospitals and the biomedical industry in China remains low. The key to promote innovative collaboration is encouraging and guiding medical staff with lower levels of education and professional titles to engage in innovative collaboration. Fortunately, medical staff in China are optimistic about their willingness of innovative collaborations with the biomedical industry, and research publications may be a key incentive to further increase their willingness.

# Abstract: 29. Interactive Clinical Diagnosis Recommendation for Chest X-ray Radiographs Through Deep Learning and Hierarchical Clustering

**Authors:** Jianguo Hao, Qing Li, Guilan Kong

**Affiliations:** ^1^ Institute of Medical Technology, Health Science Center of Peking University, Beijing, China.; ^2^ National Institute of Health Data Science, Peking University, Beijing, China.; ^3^ Advanced Institute of Information Technology, Peking University, Hangzhou, Zhejiang, China.; * Address correspondence to: guilan.kong@hsc.pku.edu.cn

**Background:** In the field of radiology, precise and efficient interpretation of radiographs is crucial for radiologists. However, radiographs usually contain multiple manifestations, making multi-label images difficult to be interpreted. This is because the presence of patterns from various manifestations in a radiograph results in exponential possible manifestation sets for consideration [1]. Compared with manual interpretation, combining the multi-label classification of radiographs with clinical diagnosis recommendation should have the capability to aid radiologists to improve diagnostic efficiency and accuracy. However, implementing such a diagnosis recommendation model or system in radiography faces two main challenges. First, it is needed to accurately identify the features existing in the target radiograph. Second, to provide accurate clinical diagnosis recommendation, it is necessary to retrieve high relevant radiographs with similar features as the target case.

**Objectives:** This study proposes an interactive recommendation model that can first help to classify multi-label chest x-ray image having multiple manifestations and then interact with radiologists to produce relevant diagnosis recommendations based on diagnoses of radiographs with similar manifestations. Furthermore, by incorporating interactive keyword and prompts filtering into diagnosis recommendation, this model could help provide more comprehensive diagnostic recommendations so as to improve diagnostic accuracy and patient outcomes.

**Methods:** The experimental data come from the Medical Information Mart for Intensive Care Chest X-ray (MIMIC-CXR) dataset [2], which is one of the largest de-identified publicly available datasets of chest radiographs. It contains multi-modal data of 65,379 patients with 227,826 imaging studies, having 14 different chest manifestations. Each patient may undergo more than one chest x-ray examination, and each preliminary diagnosis may contain multiple radiographic manifestations with certain or ambiguous judgment.

In the classification stage, multi-label chest x-ray images are classified by an initial convolutional neural network model. This model was implemented using EfficientNet [3] as the backbone network and then was adapted to the MIMIC-CXR data through transfer learning. In the recommendation stage, the formal concept analysis (FCA) [4] was used for similar radiograph clustering. It clusters chest x-ray images on the basis of different sets of manifestations (i.e., different centroids of clusters obtained by the concept-forming operators of FCA [5]) and creates a hierarchical clustering graph by subordination relationships among different clusters. Once the target image is classified, the proposed model matches the target case with the appropriate cluster having the same manifestations in the graph. After that, all the radiographs and recorded diagnoses in both matched and the adjacent clusters were recommended. Additionally, in the subsequent stage of interactive recommendation, radiologists can iteratively optimize and select more appropriate keywords or prompts based on the detected radiographic manifestations to retrieve more relevant clinical diagnosis.

During the model development, we took advantages of the recommended data splits provided by the MIMIC-CXR dataset [2] for training, validation, and test. The training dataset was used to train the multi-label classification model, and the validation dataset was used to fine-tune the hyperparameters of the model. The test dataset was used to evaluate the performance of the final model. The performance of the multi-label classification model was evaluated by the classification accuracy. For each manifestation, the classification accuracy was defined as the proportion of cases correctly classified among the total number of cases in the test dataset. The performance of recommendation was evaluated by the average recall of each case, and the recall of each recommendation was defined as the number of matched images recommended by the model divided by the total number of relevant images. Here, relevant images of an x-ray image to analyze were defined as the images having the same or similar features, including images in the cluster with the same manifestations as the centroid, and the images in its adjacent clusters with the subsets or supersets of the manifestations as the centroids.

**Results:** The average accuracy of multi-label classification for chest x-ray images in the test dataset was 86.27%. Specifically, for the cases with atelectasis, cardiomegaly, edema, no finding, pneumothorax, and support devices, the accuracies are 79.59%, 75.58%, 82.01%, 78.43%, 97.15%, and 79.55%, respectively. Due to the low prevalence (varying between 0.88% and 7.27%) in MIMIC-CXR dataset, cases with certain manifestations, such as consolidation, fracture, lung lesion, and pleural other, were mostly misclassified. For the cases where all the manifestations in a radiograph were correctly detected, the classification accuracy and the recall of recommendation were both 100%. Even in the cases where only a subset of manifestations was correctly detected, the diagnosis recommendations still remained highly relevant, because the recommended results having partial features or manifestations are still similar to the real recommendations. The interactive nature of the proposed model allows radiologists to optimize the retrieval keywords or prompts in a continuous way in order to make the manifestations as consistent as possible with the real ones shown in the input radiographs, and thus can obtain more comprehensive and relevant recommendations.

**Conclusion:** This study proposes an interactive clinical diagnosis recommendation model for chest x-ray radiographs, which first helps to classify multi-label chest x-ray images having multiple manifestations, and then to recommend diagnoses of radiographs with similar features according to a hierarchical clustering graph. The relevance of diagnosis recommendation depends on both the accuracy of multi-label classification and the subsequent interactions with radiologists. Further interactions with radiologists can help to optimize the keywords or obtain new prompts for a new round of recommendation. In next step study, we plan to generalize this interactive clinical diagnosis recommendation model to other disease areas and develop a computerized system to implement it in clinical practice.

**Acknowledgments:** This study was supported by grants from the National Key Research and Development Program of China (grant no. 2018AAA0102100), Zhejiang Provincial Natural Science Foundation of China (grant no. LZ22F020014), Beijing Municipal Science & Technology Commission (grant no. 7212201), and Humanities and Social Science Project of Chinese Ministry of Education (grant no. 22YJA630036).

## References

[1] Ge Z, Mahapatra D, Chang X, Chen Z, Chi L, Lu H. Improving multi-label chest X-ray disease diagnosis by exploiting disease and health labels dependencies. Multimed Tools Appl. 2020;79(21):14902.

[2] Khusro S, Ali Z, Ullah I. *Recommender systems: Issues, challenges, and research opportunities*Singapore: Springer Singapore; 2016.

[3] Johnson AEW, Pollard TJ, Berkowitz SJ, Greenbaum NR, Lungren MP, Deng CY, Mark RG, Horng S. MIMIC-CXR, a de-identified publicly available database of chest radiographs with free-text reports. Sci Data. 2019;6(1):317.31831740 10.1038/s41597-019-0322-0PMC6908718

[4] Tan M, Le Q. EfficientNet: Rethinking model scaling for convolutional neural networks. arXiv. 2019. 10.48550/arXiv.1905.11946.

[5] Ganter B, Wille R. *Formal concept analysis—Mathematical foundations*Berlin, Heidelberg: Springer; 1999.

# Abstract: 30. Interpretable Machine Learning for the Early Diagnosis of Kawasaki Disease: A Cross-Sectional Study in Chongqing, China

**Authors:** Yifan Duan, Ruiqi Wang, Qing Qian, Haolin Wang

**Affiliations:** ^1^ Institute of Medical Information, Chinese Academy of Medical Sciences and Peking Union Medical College, Beijing 100020, China.; ^2^ College of Medical Informatics, Chongqing Medical University, Chongqing 400016, China.; * Address correspondence to: qian.qing@imicams.ac.cn (Q.Q.); haolinwang@cqmu.edu.cn (H.W.)

Abstract Competition Winner – First Prize

**Background:** Kawasaki disease (KD) is a serious threat to newborns and children. The earlier and less expensive detection of this disease with accuracy and reliability will save many lives each year. Several smart solutions based on artificial intelligence (AI) for individual early detection of KD based on electronic medical records (EMR) employing machine learning approaches have been developed. However, integration into clinical practice is problematic due to a lack of model interpretability. In this work, real-world data were used to create KD diagnosis models utilizing classical and explainable machine learning approaches.

**Objectives:** Using a real-world dataset specifically available for this investigation, we aim to develop explainable clinical models for early diagnosis of the probability of KD in patients.

**Methods:** The patients are admitted from the Children’s Hospital of Chongqing Medical University in a de-identified format. The data are randomly divided into train and test datasets according to 70% and 30%. All data pre-processing and feature selection are performed in the train dataset to prevent the risk of “data leakage.” In the data pre-processing stage, covariates with >20% missing data were excluded; missing values were imputed using MissForest algorithm, a non-parametric method for filling in missing values, applicable to any type of data (continuous, discrete). Key clinical variables were selected by the recursive feature elimination with cross-validation (RFE-CV). According to the “No Free Lunch Theorem,” an extensive set of 14 learning algorithms was developed, including classical machine learning algorithms with strong explanations [logistic regression (LR), decision trees (DTs), etc.], classical ensemble algorithms (light gradient boosting machine, AdaBoost classifier, etc.), and explainable machine learning algorithms [explainable boosting machine (EBM)]. Early diagnosis models were selected from 14 machine learning algorithms with excellent performance, which were evaluated by area under the operating characteristic curve (AUC) and accuracy (ACC). We also incorporate LR and DTs into the early diagnosis model, although their training performance is rather mediocre; they have better interpretability. We propose three key measures to evaluate model performances (3-D evaluation): (a) discrimination: the area below the curve–the receiver operating characteristic (AUC-ROC), confusion matrix-based metrics; (b) calibration: the calibration curve; (c) explainability: the process of measuring the contribution of variables, interactions, and individual early diagnosis using the global and local interpreters of the EBM algorithm (Fig. 19).

**Fig 19.** Research flowchart and key results.

**Results:** This study included 4,087 patients (median age 2.2, 62.1% male) and 31 key clinical variables (URO, HCT, PDW, U-BIL, HGB, RDW, HBcAb, etc.). Light gradient boosting machine (LGBM), historical gradient boosting classifier (HGBC), EBM, gradient boosting classifier (GBDT), AdaBoost classifier(AdaBoost), LR, and DT were selected as the predictive models. In terms of discrimination, the LGBM model marginally outperformed all other methods achieving AUC (0.98) and ACC (0.94). EBM demonstrated high AUC (0.96) and ACC (0.93) on par with the black box models while remaining fully interpretable. In terms of calibration, LR, LGBM, and EBM have better performance. In terms of explainability, PDW, ESR, FecalWhitebloodCell, TP, and AST showed the greatest impact, according to the EBM global interpreter. These impact indicators are also largely consistent with LR and DT. The EBM model demonstrates the interaction between features; the interaction resulted to be the most predictive features. They were ranked according to the strength of the interaction as MPV&PWD and PDW&TP. EBM was also evaluated using local interpreter methods, which are beneficial for understanding individual early diagnosis and may be more accurate than global interpreter.

**Conclusion:** EBM presented good performance in 3-D evaluation and can be explained based on global interpreter, local interpreter, and interactions. It will help doctors to reveal hidden interactions of patients in clinical practice.

# Abstract: 31. Longitudinal Associations of Long-Term PM_2.5_ and Its Major Components Exposure with the Body Composition in Chinese Elderly Population

**Authors:** Yu Min, Xingchen Peng, Xiaoyuan Wei, Wenchong He, Chenyu Yang, Beibei Cui, Ke Ju

**Affiliations:** ^1^ Department of Biotherapy and National Clinical Research Center for Geriatrics, Cancer Center, West China Hospital, Sichuan University, Chengdu, China.; ^2^ Department of Head and Neck Oncology, Department of Radiation Oncology, Cancer Center, and State Key Laboratory of Biotherapy, West China Hospital, Sichuan University, Chengdu, China.; ^3^ Research Management Office, West China Second University Hospital, Sichuan University, Chengdu, China.; ^4^ Department of Big Data in Health Science, School of Public Health, Zhejiang University School of Medicine, Hangzhou, China.; ^5^ Department of Rheumatology and Immunology, West China Hospital, Sichuan University, Chengdu, China.; ^6^ School of Public Health and Preventive Medicine, Monash University, Melbourne, Australia.; * Address correspondence to: ke.ju@monash.edu (K.J.); cuibeibei_123@163.com (B.C.); † These authors contributed equally to this work.

**Background:** Changes in body composition (lean and fat mass) and body size are predictors of the early onset of diseases in elderly population. Little is known about the associations between PM_2.5_ components and changes in body compositions.

**Objectives:** We examined longitudinal associations of fine particulate matter (PM_2.5_) and its major compositions with the body mass index (BMI), predicted lean body mass index (LBMI), predicted fat mass index (FMI), predicted body roundness index (BRI), as well as a body shape index (ABSI) in elderly Chinese population.

**Methods:** This nationwide, population-based, longitudinal prospective study enrolled Chinese elderly participants from the Wave2011 and Wave2015 investigations. The concentrations of long-term PM_2.5_ and its major components were calculated by using near real-time data at a resolution of 1 km during the study years. Meanwhile, the BMI, predicted LBMI, predicted FMI, predicted BRI, and ABSI were evaluated by using the physical examinations data in two waves of interviews. A multivariable fixed-effects model was applied to investigate the association between PM_2.5_ and its components with the changes in body components in the Chinese elderly population. Robust checks were further conducted to validate the main results we determined.

**Results:** A total of 7,130 middle-aged and elderly adults were included in this study, with a mean age of 58.38 years and mean BMI of 23.64 kg/m^2^. After adjusting multiple covariates, we observe a significant decrease in LBMI [coef = −0.08 kg/m^2^, 95% confidence interval (CI): −0.14 to −0.01 kg/m^2^] but increase in BMI (coef = 0.29 kg/m^2^, 95% CI: 0.04 to 0.54 kg/m^2^), FMI (coef = −0.08 kg/m^2^, 95% CI: 0.01 to 0.15 kg/m^2^), and BRI (coef = 0.12 kg/m^2^, 95% CI: 0.03 to 0.20 kg/m^2^) with an interquartile range increase in PM_2.5_ concentration (34.45 μg/m^3^). Regarding the major components of PM_2.5_, similar correlations are also observed in OM, NH_4_^+^, NO_3_^−^, and SO_4_^2−^ with the changes in body compositions. Additionally, the old and male subpopulations are determined to be more vulnerable to the effect of PM_2.5_ and its major components on body composition changes (Fig. 20).

**Fig. 20.** The forest plot for showing the association between chronic PM_2.5_ exposure and its major components with the changes in body mass and body compositions among Chinese elderly population. (A) PM_2.5_. (B) BC. (C) OM. (D) NH_4_^+^. (E) NO_3_^−^. (F) SO_4_^2−^. BMI, body mass index; LBMI, lean body mass index; FMI, fat mass index; BRI, body roundness index; BC, black carbon; OM, organic matter.

**Conclusion:** Our analyses indicate that chronic PM_2.5_ and its components exposure are adversely associated with the changes in body compositions and body size among the middle-aged and elderly Chinese population. Our study provides evidence for supporting mitigating the risks of PM_2.5_ and ways to prevent the consequent diseases in later life of population.

**Author contributions:** Conception and design: K.J. and Y.M. Administrative support: K.J. and B.C. Provision of study materials or patients: X.W., Y.M., X.P., and W.H. Collection and assembly of data: K.J., X.W., and C.Y. Data analysis and interpretation: Y.M. and K.J. Manuscript writing: All authors. Final approval of manuscript: All authors.

**Competing interests:** The authors declare that they have no competing interests.

# Abstract: 32. Long-Term Air Pollution Exposure Associated with Incidence and Dynamic Progression of Chronic Kidney Disease: A Multi-State Analysis of UK Biobank

**Authors:** Fulin Wang, Chao Yang, Feifei Zhang, Pengfei Li, Luxia Zhang

**Affiliations:** ^1^ Institute of Medical Technology, Peking University Health Science Center, Beijing, China.; ^2^ National Institute of Health Data Science at Peking University, Beijing, China.; ^3^ Renal Division, Department of Medicine, Peking University First Hospital, Peking University Institute of Nephrology, Beijing, China.; ^4^ Research Units of Diagnosis and Treatment of Immune-mediated Kidney Diseases, Chinese Academy of Medical Sciences, Beijing, China.; ^5^ Advanced Institute of Information Technology, Peking University, Hangzhou, China.; * Address correspondence to: yangchao@bjmu.edu.cn

**Background:** Previous studies have proved that long-term ambient air pollution exposure is linked with the incidence of chronic kidney disease (CKD) and decline of estimated glomerular filtration rate (eGFR). However, evidence on the association of air pollution with the dynamic progression of CKD to different states such as end-stage renal disease (ESRD), cardiovascular disease (CVD), or death is still unclear.

**Objectives:** This study aimed to systematically investigate whether the long-term exposure to air pollution was associated with the onset, progression, and death of CKD.

**Methods:** The UK Biobank, a large-scale database containing biomedical information from more than half a million UK volunteers, was used as the data resource of this study. Participants without CKD and CVD at recruitment were included in our analysis. Instead of the common single-state model, a multi-state model was used to evaluate the association of two air pollutants, including particulate matter with a diameter of ≤2.5 μm (PM_2.5_) and nitrogen dioxide (NO_2_), with risk of various progressions of CKD. The multi-state model is an extension of classical survival analysis, which can be established to evaluate the influence of specific exposures on different stages of disease progression simultaneously. The outcomes of interest included incident CKD, ESRD, CVD diagnosed after CKD, and death. Several covariates including age, sex, ethnic, smoking, drinking, body mass index, history of diabetes, history of hypertension, and UK Biobank Centres were adjusted in the multi-state model.

**Results:** Among the 380,416 participants included, 8,379 developed CKD and 19,333 died during a median follow-up of 13.8 years. There were 86 CKD patients further progressing to ESRD, while 1,089 developed CVD after the diagnosis of CKD. Both PM_2.5_ and NO_2_ were observed to be associated with CKD incidence. Specifically, with per interquartile range (IQR) increase in PM_2.5_ (1.28 μg/m^3^) and NO_2_ (9.88 μg/m^3^), the hazard ratio (HR) for transitions from baseline to CKD was 1.117 [95% confidence interval (CI): 1.089 to 1.146] and 1.108 (95% CI: 1.077 to 1.140), respectively. Exposure to NO_2_ was also found to be linked with the increased risk from CKD to ESRD (HR: 1.393, 95% CI: 1.065 to 1.823), from CKD to CVD (HR: 1.088, 95% CI: 1.003 to 1.181), from CKD to death (HR: 1.110, 95% CI: 1.019 to 1.209), and from CVD to death (HR: 1.156, 95% CI: 1.008 to 1.326); however, the relationships between PM_2.5_ exposure and above transitions were not statistically significant (Fig. 21).

**Fig. 21.** Transitions from baseline to CKD, ESRD, CVD, all-cause death, as well as the associations between two air pollutants and risk of the eight progressions of CKD.

**Conclusion:** Our finding indicates that air pollution, especially gaseous pollutant such as NO_2_, might be a risk factor in almost all stages in the incidence and dynamic progression of CKD. Control of air pollution may be beneficial for multi-level prevention of CKD and should be paid enough attention to.

# Abstract: 33. M^3^Fair: Mitigating Bias in Healthcare Data through Multi-Level and Multi-Sensitive-Attribute Reweighting Method

**Authors:** Yinghao Zhu, Jingkun An, Enshen Zhou, Lu An, Junyi Gao, Hao Li, Haoran Feng, Bo Hou, Wen Tang, Chengwei Pan, Liantao Ma

**Affiliations:** ^1^ Institute of Artificial Intelligence, Beihang University, Beijing, China.; ^2^ National Engineering Research Center for Software Engineering, Peking University, Beijing, China.; ^3^ School of Software, Beihang University, Beijing, China.; ^4^ Department of Electrical and Computer Engineering, North Carolina State University, Raleigh, NC, USA.; ^5^ Institute of Genetics and Cancer, University of Edinburgh, Edinburgh, UK.; ^6^ Health Data Research UK, London, UK.; ^7^ Department of Nephrology, Peking University Third Hospital, Beijing, China.; * Address correspondence to: pancw@buaa.edu.cn (C.P.); malt@pku.edu.cn (L.M.); † These authors contributed equally to this work.

**Background:** In the data-driven artificial intelligence paradigm, models heavily rely on large amounts of training data. However, factors like sampling distribution imbalance can lead to issues of bias and unfairness in healthcare data. Sensitive attributes (SAs), such as race, gender, age, and medical condition, are characteristics of individuals that are commonly associated with discrimination or bias. In healthcare AI, these attributes can play a significant role in determining the quality of care that individuals receive. For example, minority groups often receive fewer procedures and poorer-quality medical care than white individuals in the US [1]. Therefore, detecting and mitigating bias in data is crucial to enhancing health equity.

Bias mitigation methods include pre-processing, in-processing, and post-processing [2]. Among them, reweighting (RW) is a widely used pre-processing method that performs well in balancing machine learning performance and fairness performance [2]. RW adjusts the weights for samples within each (group, label) combination, where these weights are utilized in loss functions [3]. However, RW is limited to considering only a single SA when mitigating bias and assumes that each SA is equally important. This may result in potential inaccuracies when addressing intersectional bias [4].

To address these limitations, we propose M^3^Fair, a multi-level and multi-sensitive-attribute RW method, by extending the RW method to multiple SAs at multiple levels. Our experiments on real-world datasets show that the approach is effective, straightforward, and generalizable in addressing the healthcare fairness issues.

**Objectives:** (a) Detect and identify source(s) of biases. There may be multiple features that could potentially lead to bias. These features are defined as SAs. (b) Mitigate bias impact of SAs while balancing machine learning and fairness performance, ensuring model effectiveness, accuracy, and equity in healthcare AI.

**Methods:** *Bias Detection:* To identify SAs, we consider features that consistently exhibit biased tendencies across various bias evaluation metrics as SAs. Concretely, we first binarize each feature by comparing its mean value. We then iterate through each feature, calculating its performance on four evaluation metrics: disparate impact (DI), statistical parity difference (SPD), average odds difference (AOD), and equal opportunity difference (EOD) [2]. These metrics reflect different aspects of bias as they capture different ways in which privileged groups can be treated unfairly [5], and their combined use allows us to identify features that consistently exhibit bias across multiple dimensions. Thus, we take the intersection of the top *N* most unfair features based on these metrics (default *N* = 20). Our experiment results show that features such as race and age consistently exhibit bias across these metrics, highlighting the importance of identifying and mitigating bias based on multiple SAs simultaneously.

*Bias Mitigation:* We first define sensitivity levels (SL) as the sum of level weights for multiple SAs associated with a sample, allowing us to assign level weights to each SA. Next, M^3^Fair calculates the sum of level weights for samples with or without favorable labels. The weight coefficients for samples are then computed based on Eq. 1. by iterating each SL.

(1) $$W_{i,y_{i}=d}^{\prime }=W_{i}\frac{\sum_{y_{j}=d}W_{j}\cdot \sum_{\mathrm{SL}}W_{j}}{\sum W_{j}\cdot \sum_{y_{j}=d,\mathrm{SL}}W_{j}}$$

where *d* ∈ {0, 1}, *d* = 1 means the sample has a favorable label (typically class 1) and *d* = 0 means it has an unfavorable label (typically class 0). *W* is the sample weight. The calculated sample weights *W*^’^ can be simply applied in the loss function of models.

**Results:** Table 1 shows our experiment results. EA denotes the evaluated attribute. A desirable score for DI is the score close to 1, while SPD, AOD, and EOD metrics are the lower, the better. The notation A->B requires RW twice, first by attribute A and then by B, while the notation [A, B] means that A and B attributes are reweighted simultaneously. The model used is a logistic regression model. We choose the SL setting of Sex = 1, Race = 2, Age = 2 in the M^3^Fair method through grid search in the search space of {1, 2}.

**Table 1.** Experiment results on Adult, TJH, and CDSL datasets

Data	Method	SA	EA	ACC	AUROC	AUPRC	DI	SPD	AOD	EOD
Adult	/	/	Sex	0.8007	0.8216	0.7300	0.3635	−0.1989	−0.2089	−0.1672
			Race				0.6038	−0.1040	−0.0886	−0.0604
	RW	Sex->Race	Sex	0.7856	0.8138	0.7216	0.9786	0.0534	−0.0583	0.0111
			Race				1.0000	0.0000	−0.0112	0.0223
		Race->Sex	Sex	0.7857	0.8141	0.7219	1.0000	0.0000	−0.0631	0.0081
			Race				0.913	0.0232	0.0069	0.0356
	M^3^Fair	[Sex, Race]	Sex	0.7858	0.8143	0.7221	1.0000	0.0000	−0.0627	0.0078
			Race				1.0000	0.0000	−0.0106	0.0214
TJH	/	/	Sex	0.8349	0.8357	0.8696	0.5454	−0.2573	−0.0699	−0.0018
			Age				0.2782	−0.4824	−0.2585	−0.2922
	RW	Sex->Age	Sex	0.8073	0.8095	0.8500	0.9789	0.0098	−0.0392	−0.0281
			Age				1.0000	0.0000	−0.2191	−0.3160
		Age->Sex	Sex	0.8073	0.8095	0.8500	1.0000	0.0000	−0.0392	−0.0281
			Age				0.9891	0.0050	−0.2191	−0.3160
	M^3^Fair	[Sex, Age]	Sex	0.8073	0.8095	0.8500	1.0000	0.0000	−0.0392	−0.0281
			Age				1.0000	0.0000	−0.2191	−0.3160
CDSL	/	/	Sex	0.6766	0.7254	0.5397	0.1374	−0.1858	−0.5453	−0.3878
			Age				0.8368	−0.0222	−0.1077	−0.2002
	RW	Sex->Age	Sex	0.7776	0.6497	0.4175	0.8986	0.0134	−0.0677	−0.0685
			Age				1.0000	0.0000	−0.0483	0.0270
		Age->Sex	Sex	0.8081	0.6547	0.4248	1.0000	0.0000	−0.0084	−0.0170
			Age				0.9806	−0.0025	−0.0354	0.0608
	M^3^Fair	[Sex, Age]	Sex	0.8121	0.6569	0.4285	1.0000	0.0000	−0.0027	−0.0170
			Age				1.0000	0.0000	−0.0268	0.0608

We conducted experiments on the UCI Adult dataset and two real-world COVID-19 EHR datasets, TJH and CDSL [6]. As shown in Table 1, compared to not using any mitigation methods, our M^3^Fair method successfully mitigates biases introduced by SAs such as gender, age, and race in 91.67% (22/24) metrics while slightly affecting model performance with an average performance drop of 2.51%. Furthermore, M^3^Fair shows 100% better (12 metrics) or equal (12 metrics) fairness performance than the baseline RW method on all four metrics.

**Conclusion:** The proposed method M^3^Fair focuses on the bias within the dataset. We detect SAs by analyzing consistently biased features. By extending the existing RW method, we support multiple SLs and SAs. Experiments demonstrate that M^3^Fair with considering multiple SAs successfully mitigates bias in 91.67% metrics and performs better or equivalent than a single attribute in all metrics. Our method is generalizable and scalable, allowing for customization of sensitive levels in clinical decision-making processes. The code is open-sourced at https://github.com/yhzhu99/M3Fair.

Our solution won the NIH Bias Detection Tools for Clinical Decision Making Challenge and was evaluated by NIH as “performed well in the test harness and had a relatively strong performance across social and predictive fairness metrics. The approach is straightforward, and very generalizable to any number of use-cases.”

## References

[1] Hall WJ, Chapman MV, Lee KM, Merino YM, Thomas TW, Payne BK, Eng E, Day SH, Coyne-Beasley T. Implicit racial/ethnic bias among health care professionals and its influence on health care outcomes: A systematic review. Am J Public Health. 2015;105(12):e60–e76.10.2105/AJPH.2015.302903PMC463827526469668

[2] Chen Z, Zhang JM, Sarro F, Harman M. A comprehensive empirical study of bias mitigation methods for machine learning classifiers. ACM Trans Softw Eng Methodol. 2023;32(4):1–30.

[3] Kamiran F, Calders T. Data preprocessing techniques for classification without discrimination. Know Inf Syst. 2012;33(1):1–33.

[4] Ogungbe O, Mitra AK, Roberts JK. A systematic review of implicit bias in health care: A call for intersectionality. *IMC J*. Med Sci. 2019;13(1):005.

[5] Feldman M, Friedler SA, Moeller J, Scheidegger C, Venkatasubramanian S. Certifying and removing disparate impact. Paper presented at: Proceedings of the 21th ACM SIGKDD International Conference on Knowledge Discovery and Data Mining, Sydney NSW, Australia; 2015. pp. 259–268.

[6] Gao J, Zhu Y, Wang W, Wang Y, Tang W, Ma L. A comprehensive benchmark for COVID-19 predictive modeling using electronic health records in intensive care: Choosing the best model for COVID-19 prognosis. arXiv. 2022. https://arxiv.org/abs/2209.07805.10.1016/j.patter.2024.100951PMC1102696438645764

# Abstract: 34. Modeling-Based Prediction Tools for Mitochondrial DNA Diseases

**Authors:** Ning Zhang, Yuzhou Gao, Yinan Du, Yunxia Cao, Dongmei Ji

**Affiliations:** ^1^ Department of Obstetrics and Gynecology, First Affiliated Hospital of Anhui Medical University, No 218 Jixi Road, Hefei 230022, Anhui, China.; ^2^ School of Basic Medical Sciences, Anhui Medical University, Hefei 230032, Anhui, China.; ^3^ First School of Clinical Medicine, Anhui Medical University, Hefei 230032, Anhui, China.; ^4^ NHC Key Laboratory of Study on Abnormal Gametes and Reproductive Tract (Anhui Medical University), No 81 Meishan Road, Hefei 230032, Anhui, China.; ^5^ Key Laboratory of Population Health Across Life Cycle (Anhui Medical University), Ministry of Education of the People’s Republic of China, No 81 Meishan Road, Hefei 230032, Anhui, China.; * Address correspondence to: jidongmei@ahmu.edu.cn

**Background:** Mitochondrial diseases are among the most common inherited metabolic genetic disorders. Pathogenic mutations in mitochondrial DNA (mtDNA) cause at least 15% of these disorders. However, the pattern of inheritance of these mutations is not fully comprehended. Pre-implantation genetic testing (PGT) is a dependable tool for preventing the germline transmission of mtDNA mutations. Nonetheless, procedures are not standardized across different mtDNA mutations, and the best approach for patients with uncommon mtDNA variants is often unclear.

**Objectives:** This study aimed to establish standardized tools that predict genetic threshold, risk of disease transmission, and the number of oocytes needed for PGT.

**Methods:** We conducted a systematic analysis of heteroplasmy data from 455 individuals in 187 familial pedigrees with the m.3243A>G, m.8344A>G, or m.8993T>G pathogenic mutations. To estimate symptomatic thresholds of heteroplasmy, predict the risk of disease transmission, and determine the minimum number of oocytes required for preimplantation genetic testing, we applied binary logistic regression, simplified Sewell–Wright formula and Kimura equations, and binomial distribution, respectively.

**Results:** We used our models to estimate the symptomatic thresholds of m.8993T>G and m.8344A>G as 29.86% and 16.15%, respectively, but could not determine a threshold for m.3243A>G. Additionally, we developed models to predict the risk of disease transmission and the minimum number of oocytes required to produce an embryo with a low mutation load for mothers with both common and rare mtDNA point mutations. We modeled the data of three patients with rare pathogenic mtDNA mutations from our clinic: P1 (m.9185T>G), P2 (m.3697G>A), and P3 (m.10191A>G) (Fig. 22A to D). We predicted the probability of offspring inheriting different mutation levels, the risk of disease transmission without intervention, and the minimum number of oocytes needed for preimplantation genetic testing (PGT). Our analysis revealed that the probability of disease transmission for P1 was 61.08%, and the minimum number of oocytes required for PGT was 13 (Fig. 22B); for P2, the probability was 84.64%, and the minimum number of oocytes required for PGT was 37 (Fig. 22C); and for P3, the probability was 28.55%, and the minimum number of oocytes required for PGT was 5 (Fig. 22D). In addition, we provided a table allowing the prediction of transmission risk and the minimum required oocytes for PGT patients with different mutation levels (Fig. 22E).

**Fig. 22.** Prediction of risk of disease transmission and minimum number of oocytes required for PGT using our universal prediction model. (A) Rare pathogenic mtDNA mutations carried by patients (mean mtDNA mutation load value from blood and muscle). (B) Probability of patient 1 having offspring with different mutation loads. (C) Probability of patient 2 having offspring with different mutation loads. (D) Probability of patient 3 having offspring with different mutation loads. Dashed lines indicate the universal value of pathogenicity threshold (18%). (E) Assuming that every mtDNA mutation follows the same role of transmitting pattern through the bottleneck, for patients with certain mutation loads (1% to 60%) of any mtDNA mutation, risk of disease transmission and minimum number of oocytes required for PGT were modeled.

**Conclusion:** We have developed models to determine symptomatic thresholds of common mtDNA point mutations. Additionally, we have created universal models, which can predict risk and minimum numbers for PGT patients for nearly all mtDNA point mutations. These models have significantly improved our understanding of mtDNA disease pathogenesis and can facilitate more effective prevention of disease transmission through PGT.

# Abstract: 35. Multi-Modal Medical Vision-and-Language Learning for Retinal Vein Occlusion Classification

**Authors:** Weibin Liao, Yanfeng Liao, Zhimin Fan, Jiexuan Zhang, Shuochen Li, Jiarui Yang, Liantao Ma

**Affiliations:** ^1^ National Engineering Research Center for Software Engineering, Peking University, Beijing, China.; ^2^ Computer Science and Technology, Beijing Institute of Technology, Beijing, China.; ^3^ Department of Ophthalmology, Peking University Third Hospital, Beijing Key Laboratory of Restoration of Damaged Ocular Nerve, Peking University Third Hospital, Beijing, China.; * Address correspondence to: jiaruiyang@bjmu.edu.cn (J.Y.); malt@pku.edu.cn (L.M.); † These authors contributed equally to this work.

**Background:** Retinal vein occlusion (RVO) is one of the most common retinal vascular diseases leading to vision loss if not diagnosed and treated in time. Automated diagnosis of central and branch RVO (CRVO and BRVO) can alleviate the workload of ophthalmologists while facilitating early detection and treatment of RVO and laying the foundation for subsequent symptom grading, treatment planning, and follow-up. Therefore, the development of a fast, high-performance, and robust diagnostic model is a crucial step toward achieving quantitative and accurate assessment of RVO disease. While there is an extensive research using fundus visual images for clinical assisted diagnosis with deep learning models, there is a lack of focus on incorporating doctors, text reports to further enhance the models.

**Objectives:** In this study, we propose a multi-modal medical visual-and-language learning model that utilizes fundus fluorescein angiography (FFA) images and physician analysis reports to classify BRVO and CRVO cases.

**Methods:** As shown in Fig. 23, the proposed model utilizes an advanced convolutional neural network to extract visual features from FFA images for patient visual representation learning. For language representation learning, the model first extracts basic patient features such as gender, age, vision, and blood pressure, and then uses regular expression matching to obtain typical patient symptoms from expert text reports. Specifically, we created a sign vocabulary for RVO patients, including exudate, macular edema, and intraretinal hemorrhage, among others. Based on this vocabulary, the proposed model analyzes patient symptom manifestations and learns symptom presentation (such as exudate stage and location). Finally, based on the patient,s visual and text representation, the proposed model uses a fully connected (FC) layer for classification tasks.

**Fig. 23.** The framework of the multi-modal medical vision-and-language learning model.

**Results:** We evaluated the proposed model on a private dataset consisting of 101 patients and 1,265 FFA images, composed of 58 BRVO patients and 43 CRVO patients. To better demonstrate the performance of the model, we conducted stratified sampling based on patients and reported the results of the fivefold cross-validation. The experimental results are shown in Table 1. The experimental results show that the multi-modal vision-and-language learning model achieved the best performance on all evaluation metrics except recall in RVO classification task. Furthermore, we observed that FFA images play a major role in this task, and the model using only visual representation achieved an accuracy of 80.72%. However, the text report only contributed marginally to the prediction accuracy (55.76%), but it still provides potential information that is difficult to discover from visual images, which can further improve the accuracy [84.27% (3.55%↑)] and robustness [1.69% (0.76%↓)] of the model.

**Table 1.** Performance of the proposed model for RVO classification

Metrics	Acc.	Pre.	Rec.	F1	AUPRC	AUROC
Text	55.76 ± 6.13	58.52 ± 5.60	81.11 ± 17.78	67.04 ± 7.91	67.17 ± 8.11	58.39 ± 7.69
Image	80.724 ± 2.45	81.74 ± 6.27	85.46 ± 6.74	83.10 ± 2.43	93.93 ± 1.37	91.12 ± 2.21
Text + Image	84.27 ± 1.69	87.95 ± 3.57	83.71 ± 4.92	85.58 ± 1.48	94.25 ± 0.69	91.60 ± 0.91

**Conclusion:** In this study, we propose a multi-modal medical vision-and-language learning model for RVO classification tasks. The proposed model uses advanced deep neural networks to extract visual features from FFA images and integrates patient reports provided by doctors to analyze clinical symptoms, further enhancing the model,s performance. The experimental results demonstrated the accuracy and robustness of the proposed model, which contributes to RVO screening and management in clinical practice. Furthermore, the model provides an automated diagnostic tool for conducting further research such as automated perfusion assessment and treatment planning.

# Abstract: 36. Sleep Apnea Further Increases Cardiovascular Disease Risk in Individuals with Atrial Fibrillation

**Authors:** Jingya Wang, Christina Antza, Xian Shen, Tiffany Gooden, Abd A Tahrani, Deirdre A Lane, Anuradhaa Subramanian, Krishna Gokhale, Nicola J Adderley, Rajendra Surenthirakumaran, Paulo A Lotufo, Yutao Guo, Hao Wang, G. Neil Thomas, Gregory Y. H. Lip, Krishnarajah Nirantharakumar

**Affiliations:** ^1^ Institute of Applied Health Research, University of Birmingham, Birmingham, UK.; ^2^ Institute of Metabolism and Systems Research, University of Birmingham, Birmingham, UK.; ^3^ University Hospitals Birmingham, Birmingham, UK.; ^4^ Department of Diabetes and Endocrinology, University Hospitals Birmingham NHS Foundation Trust, Birmingham, UK.; ^5^ Centre for Endocrinology, Diabetes and Metabolism (CEDAM), Birmingham Health Partners, Birmingham, UK.; ^6^ Liverpool Centre for Cardiovascular Science, University of Liverpool and Liverpool Heart & Chest Hospital, Liverpool, UK.; ^7^ Department of Community and Family Medicine, Faculty of Medicine, University of Jaffna, Jaffna, Sri Lanka.; ^8^ University Hospital, University of São Paulo, São Paulo, Brazil.; ^9^ Chinese PLA General Hospital, Beijing, China.; * Address correspondence to: g.n.thomas@bham.ac.uk, gneilthomas@gmail.com (G.N.T.); Gregory.Lip@liverpool.ac.uk (G.Y.H.L.); † Co-directors of the NIHR Global AF Reach Group and joint senior authors.

On behalf of The NIHR Global AF Reach Group

NIHR Global Health Group on Atrial Fibrillation Management Investigators (alphabetical): Ajini Arasalingam, Abi Beane, Isabela Bensenor, Peter Brocklehurst, Kar Keung Cheng, Itamar de Souza Santos, Mei Feng, Alessandra Goulart, Sheila Greenfield, Yutao Guo, Mahesan Guruparan, Gustavo Gusso, Tiffany Gooden, Rashan Haniffa, Lindsey Humphreys, Kate Jolly, Sue Jowett, Balachandran Kumarendran, Emma Lancashire, Deirdre A Lane, Xuewen Li, Gregory Y. H. Lip (co-PI), Yan-guang Li, Trudie Lobban, Paulo Lotufo, Semira Manseki-Holland, David Moore, Krishnarajah Nirantharakumar, Rodrigo Olmos, Carla Romagnolli Quintino, Alena Shantsila, Isabelle Szmigin, Kumaran Subaschandren, Rajendra Surenthirakumaran, Meihui Tai, G. Neil Thomas (co-PI), Hao Wang, Jingya Wang.

**Objectives:** Obstructive sleep apnea (OSA) and atrial fibrillation (AF) are well-recognized risk factors for cardiovascular diseases and often coexist. We investigated whether the presence of incident OSA in individuals with AF would further increase their risk of ischemic heart disease (IHD), heart failure (HF), and stroke or transient ischemic attack (TIA).

**Methods:** Three separate propensity-score matched cohorts were generated for IHD (*n* = 5,140), HF (*n* = 6,372), and stroke/TIA (*n* = 6,372) using an AF pool with 277,684 eligible participants (2000–2018) derived from the IQVIA Medical Research Data (IMRD), a large UK-based primary care dataset. Competing risk Cox proportional hazard regression models were used to compare the risk of developing IHD, HF, and stroke/TIA between individuals with and without OSA during the follow-up period.

**Results:** All baseline characteristics between individuals with and without OSA were well balanced after matching. The median follow-up period was 3 (interquartile range: 1 to 5) years. Compared to individuals without OSA, individuals with OSA have higher risks of IHD (adjusted hazard ratios, 1.94; 95% CI, 1.42 to 2.65) and HF (1.31; 95% CI, 1.07 to 1.62) and an unchanged risk of stroke/TIA (1.06; 95% CI, 0.80 to 1.41).

**Conclusion:** OSA in individuals with AF was associated with additional increased risk of IHD and HF, not with stroke/TIA. Further studies on the effect of active screening and management of OSA to prevent cardiovascular disease in individuals with AF are warranted. Individuals with AF may also benefit from including OSA as a part of clinical decision framework for the management of cardiovascular disease.

# Abstract: 37. Physicians’ Responsibilities in the Context of Large Language Models: Now and Future

**Authors:** Jiaming Li, Di Zhang, Li Hou

**Affiliations:** ^1^ Institute of Medical Information, Chinese Academy of Medical Sciences and Peking Union Medical College, Beijing 100020, China.; ^2^ School of Humanities and Social Sciences, Chinese Academy of Medical Sciences and Peking Union Medical College, Beijing 100730, China.; * Address correspondence to: zhangdi87@outlook.com (D.Z.); hou.li@imicams.ac.cn (L.H.)

**Background:** Large language models (LLMs) have shown strong natural language understanding and generation abilities. This ability gives LLMs a wide range of potential applications in the medical field, with the potential to enhance medical efficiency and patient safety and health and promote access to healthcare. While some physicians hope to improve the efficiency of medical practices through LLMs, attempts to apply LLMs as an auxiliary have also raised ethical considerations about what responsibilities physicians should have with using LLMs. This is particularly significant when LLMs are as clinical decision support (CDS).

**Objectives:** This study reviews the potential benefits and ethical challenges of LLMs in healthcare and then argues the physicians’ responsibilities when they use LLMs as CDS in the near term and long term.

**Methods:** This study uses a literature review to sort out the applications of LLMs in healthcare and the ethical issues it raises, and explores the near-term and long-term responsibilities of physicians when LLMs are used as CDS through reflective equilibrium and 3 fundamental principles and 10 professional responsibilities from “a physicians, charter.”

**Results:** LLMs have wide potential applications in healthcare, with the potential to improve efficiency, patient safety and health, and access to care. At the meantime, four types of ethical issues have also emerged, including outcome bias, autonomy, social justice, and responsibility. Whether an LLM is a moral agent or not, its accuracy in CDS affects the physician,s ethical responsibilities. Currently, LLMs do not have free will, nor are they moral agents, and they are not ethically responsible entities (although LLMs may become moral agents and take ethical responsibilities in the future). With the continuous improvements, the accuracy of LLMs in CDS will continue to improve and partially or even fully surpass the physicians, abilities of diagnosis and treatment.

**Conclusion:** The physician is responsible for the final diagnosis and treatment decisions when the LLM is not a moral agent, regardless of whether the physician,s competencies are partially or fully exceeded by the LLM. Physicians should understand the output of the LLM through interactions with it and make judgments in conjunction with their individual competencies. When an LLM exceeds physicians, competence, the physician has an ethical responsibility to accept the results of the LLM based on the principle of primacy of patients, welfare. Based on the principle of patients, autonomy, the physician should inform the patients of the output of the LLM and the physician,s judgment to help the patients to understand that information, and respect the patients’ right to informed consent.

# Abstract: 38. Potential Schizophrenia Disease-Associated Gene Prediction Using Metagraph Representations of Proteins Based on PPI-Keywork Network

**Authors:** Shirui Yu, Ziyang Wang, Jiale Nan, Xuemei Yang, Xiaoli Tang

**Affiliations:** Institute of Medical Information/Medical Library, Chinese Academy of Medical Science and Peking Union Medical College, Beijing 100005, China.; * Address correspondence to: tang.xiaoli@imicams.ac.cn

**Background:** Schizophrenia is a serious mental disease characterized by abnormalities in thinking and cognition [1], whose occurrence is widely believed to be closely related to genetics and gene expression [2]. Searching for the associations between diseases and genes allows an in-depth exploration of the mechanisms and molecular basis of diseases.

PPI networks are the most widely used ones for disease–gene association prediction. However, existing PPI networks are often incomplete and noisy; thus, it is necessary to build heterogeneous network for disease gene prediction [3].

Recently, a general framework that considers heterogeneity by defining type-specific graphs was proposed [4]. The advantage of metagraph-based approaches is that they can preserve the network structure and provide a flexible way to explore a diverse set of descriptors.

**Objectives:** In this work, we attempt to integrate the PPI network with the keywords in UniProt database and form a heterogeneous network called PPI-Keyword (PPIK) network for disease protein prediction. Based on PPIK network, we extend the metagraph methodology to predict the probability that an association between a gene and disease exists. Each protein is represented as a series of metagraphs.

**Methods:** We proposed a metagraph representations based on the PPIK network to improve schizophrenia-associated gene prediction. The general framework mainly contains four steps, as summarized in Fig. 24. First, we obtained protein–protein interactions (PPIs) from STRING database and exploited protein keywords from the UniProt database. These two sources of data were integrated to construct the PPIK network. Second, we mined the collection of metagraphs from the PPIK network. Seven unique typed metagraphs were extracted from the network, with each representing a feature of proteins’ biological interpretation. Third, we derived the metagraph representations for proteins based on the mined metagraphs. We employed the metric called degree-weighted path count (DWPC) to summarize the contribution of each type of metagraph. Fourth, a series of metagraphs corresponding to each protein were transformed into vector representation and form an ML-ready dataset. We used the dataset as input to train the model across various machine learning techniques including LightGBM, XGBoost, and random forest, which aims to predict potential schizophrenia-associated proteins. We analyzed Shapley additive explanations (SHAP) value and adopt the same model on PPI network and PPIK network to verify the feasibility of integrating keywords. We also evaluated SHAP value and compared our model to other baselines including RWR and PRINCE to prove the benefit of metagraph representations. Based on AUC performances, we chose the model with the best performance for disease protein predictions and make further analysis of these results. Finally, we transformed our predicted disease proteins to their producer gene IDs and identified the top 20 genes as the most probable schizophrenia risk genes.

**Fig. 24.** General framework of the proposed method.

**Results:** Result show that the metagraph features we extract are effective and the proposed strategy can improve prediction and outperform other baseline models. Among three machine learning models we adopt in this study, LightGBM has the best performance, with an AUC performance of 0.855. We use the model with the best performance to make prediction and further search in the literature for evidence. We evaluate these genes in terms of gene ontology function, gene-enriched signaling pathways, and gene function clustering and find them to have relevance to schizophrenia in different aspects.

**Conclusion:** In this study, we integrated PPI and biological keywords of proteins to construct a PPIK network. Based on the PPIK network, we further proposed metagraph representations for proteins. Our method can indeed better predict disease proteins. Finally, we examine the top 20 predicted genes through literature search and gene set enrichment analysis. We considered these genes as potential schizophrenia risk genes. These genes are EYA3, CNTN4, HSPA8, LRRK2, and AFP.

## References

[1] Zhou J, Ma C, Wang K. Rare and common variants analysis of the EMB gene in patients with schizophrenia. BMC Psychiat. 2020;20(1):135.10.1186/s12888-020-02513-3PMC709397632213169

[2] Fromer M, Roussos P, Sieberts SK. Gene expression elucidates functional impact of polygenic risk for schizophrenia. Nat Neurosci. 2016;19(11):1442–1453.27668389 10.1038/nn.4399PMC5083142

[3] Jha K, Saha S, Singh H. Prediction of protein–protein interaction using graph neural networks. Sci Rep. 2022;12(1):8360.35589837 10.1038/s41598-022-12201-9PMC9120162

[4] Himmelstein DS, Baranzini SE. Heterogeneous network edge prediction: A data integration approach to prioritize disease-associated genes. PLOS Comput Biol. 2015;11(7): Article e1004259.26158728 10.1371/journal.pcbi.1004259PMC4497619

# Abstract: 39. Radiomics-Based Prediction of Pathological Complete Response to Neoadjuvant Chemoradiotherapy in Locally Advanced Esophageal Squamous Cell Carcinoma

**Authors:** Baihua Luo, Kunwei Li, Yi Hu, Man Li, Hong Shan, Duanduan Chen, Shuaitong Zhang

**Affiliations:** ^1^ School of Medical Technology, Beijing Institute of Technology, Beijing, P. R. China.; ^2^ Guangdong Provincial Key Laboratory of Biomedical Imaging and Guangdong Provincial Engineering Research Center of Molecular Imaging, Fifth Affiliated Hospital of Sun Yat-sen University, Zhuhai, Guangdong, P. R. China.; ^3^ Department of Radiology, Fifth Affiliated Hospital of Sun Yat-sen University, Zhuhai, Guangdong, P. R. China.; ^4^ Department of Thoracic Surgery, Sun Yat-sen University Cancer Center, Guangzhou, Guangdong, P. R. China.; ^5^ State Key Laboratory of Oncology in South China, Guangdong Esophageal Cancer Institute, Collaborative Innovation Center for Cancer Medicine, Guangzhou, Guangdong, P. R. China.; ^6^ Department of Interventional Medicine, Fifth Affiliated Hospital of Sun Yat-sen University, Zhuhai, Guangdong, P.R. China.; * Address correspondence to: shanhong@mail.sysu.edu.cn (H.S.); duanduan@bit.edu.cn (D.C.); zhangshuaitong@bit.edu.cn (S.Z.); † These authors contributed equally to this work.

**Background:** Esophageal cancer is a highly prevalent malignant tumors and a serious threat to human health and lives. In 2020, there were 604,100 new cases of esophageal cancer and 544,076 deaths worldwide, leading to a huge disease burden. In China, more than 90% of esophageal cancer cases are esophageal squamous cell carcinoma (ESCC).

The standard treatment for locally advanced ESCC patients is neoadjuvant chemoradiotherapy followed by surgery. After neoadjuvant chemoradiotherapy, nearly 45% of patients achieve a pathological complete response (pCR), which refers to the absence of any residual tumor cells in all resected specimens. These patients are more likely to benefit from organ- and function-preserving strategies such as active surveillance and surgery on demand. Therefore, preoperative prediction of pCR after neoadjuvant chemoradiotherapy is critical for individualized treatment guidance in ESCC patients.

In recent years, radiomics has emerged as a promising approach to support clinical decision making. It can convert medical images into high-throughput and quantitative features and then associates these features with underlying pathophysiology. Contrast-enhanced computed tomography (CT) plays a vital role in the diagnosis and treatment response evaluation in ESCC patients. Thus, we hypothesize that radiomic features from contrast-enhanced CT images after neoadjuvant chemoradiotherapy are of predictive value for pCR in ESCC patients.

**Objectives:** The aim of this study is to develop a radiomics model for predicting pCR after neoadjuvant chemoradiotherapy in locally advanced ESCC patients. Such a model could help clinicians in identifying which patient requires further surgical intervention.

**Methods:** A total of 116 locally advanced ESCC patients who received neoadjuvant chemoradiotherapy between October 2011 and December 2018 were retrospectively enrolled from Sun Yat-sen University Cancer Center. These patients were randomly divided into a training cohort (*n* = 77) and a validation cohort (*n* = 39) at a ratio of 2:1. According to the absence of viable cancer cells in the resected tumor and LNs, treatment response was recorded as pCR or non-pCR. Arterial phase contrast-enhanced CT images were obtained for all patients, and a senior radiologist delineated the tumor region on these images. To quantify these regions, 702 radiomic features were extracted using the Pyradiomics library, among which features with variance lower than 1 were removed. *Z*-score normalization was applied to eliminate the impact of unit and scale differences among features. Afterward, a three-step feature selection procedure including Mann–Whitney *U* test, Pearson correlation coefficient, and the least absolute shrinkage and selection operator (LASSO) was used in the training cohort to select the most predictive feature subset. With the selected features, the predictive models were developed using four machine learning classifiers including support vector machine with radial basis function kernel, linear support vector machine (LSVM), decision tree, and random forest. The predictive performance of these models was evaluated using the area under the curve (AUC), accuracy, sensitivity, and specificity.

**Results:** Among these 116 ESCC patients included in this study, 59 patients (50.9%) achieved pCR after receiving neoadjuvant therapy, while 57 (49.1%) failed to achieve pCR. The median age was 58.8 (range, 41 to 70) years, and 100 (86.2%) were male. The three-step feature selection procedure identified four radiomic features: maximum value, minimum value, large area high gray level emphasis, and cluster shade. Based on these four features, four classification models were developed in the training cohort, achieving AUC values ranging from 0.745 to 0.878 in the training cohort and from 0.624 to 0.710 in the validation cohort (Table 1). Among these four models, the LSVM-based model had the best predictive performance in the validation cohort.

**Table 1.** The performance of different models for predicting pathological complete response

Classifier	Training cohort				Validation cohort			
	AUC	Accuracy	Sensitivity	Specificity	AUC	Accuracy	Sensitivity	Specificity
RBF-SVM	0.787	0.753	0.684	0.821	0.677	0.615	0.524	0.722
LSVM	0.771	0.740	0.684	0.795	0.710	0.641	0.524	0.778
DT	0.745	0.740	0.631	0.846	0.665	0.615	0.524	0.722
RF	0.878	0.805	0.895	0.718	0.624	0.641	0.714	0.556

RBF-SVM, support vector machine with radial basis function kernel; LSVM, linear support vector machine; DT, decision tree; RF, random forest.

**Conclusion:** The developed radiomics model could show potential in predicting pCR and might aid treatment decision-making in ESCC patients.

# Abstract: 40. Regression Models Can Predict the Response of Low-Grade Glioma Patients to Temozolomide from Tumor Multiomics

**Authors:** H. Du, C. Piyawajanusorn, G. Ghislat, P. J. Ballester

**Affiliations:** ^1^ Department of Bioengineering, Imperial College, London, UK.; ^2^ Department of Life Sciences, Imperial College, London, UK.; * Address correspondence to: ghita.ghislat@imperial.ac.uk (G.G.); p.ballester@imperial.ac.uk (B.P.J.)

Low-grade gliomas (LGGs) are a type of slow-growing primary intracranial tumors. The treatment of LGG depends on many factors, such as the location and extent of the tumor. The most common course of treatment is surgical resection, followed by radiation therapy or chemotherapy or both, followed by regular postoperative observation [1]. Among the first-line chemotherapeutic drugs, temozolomide has relatively lower toxicity compared with procarbazine, lomustine, and vincristine and is thus preferred by most clinicians [2,3].

The efficacy of temozolomide does, however, vary across LGG patients. High O-6 methylguanine DNA transferase (MGMT) promoter methylation has been proposed as a marker of temozolomide response [4]. Unfortunately, in practice, LGG patient response to this drug is not well predicted by this univariate biomarker [5]. Therefore, there is a need for more effective ways of identifying which LGG patients will not respond to temozolomide so that these can be administered more promising drugs without delay.

The application of artificial intelligence (AI) for optimal patient treatment selection is a promising approach to precision oncology. It is, however, a less developed AI application than predicting cancer diagnosis from tumor images due to issues such as data inconsistency, proxy bias, and data fragmentation. To predict drug treatment response, the AI algorithm builds a model capturing synergistic combinations of tumor features selected out of the typically many thousands that are considered. This approach has already identified models retrospectively able to predict breast cancer patient to paclitaxel [6] and doxorubicin [7]. Here, we show that it is possible to predict LGG patient response to temozolomide following a similar AI protocol.

With this purpose, we retrieved and integrated a broad datasets comprising 109 temozolomide-treated patients labeled with their annotated RECIST responses along with 6 molecular profiles per patient: miRNA, isomiR, mRNA expression level (FPKM and FPKM-UQ), DNA methylation, and copy number variation. For each of these 6 datasets, we assessed 6 widely used regression algorithms along with their optimal model complexity (OMC) for enhanced feature selection. This was carried out by treating the 4 RECIST categories as numerical labels. In total, 12 models were built and their 10 × 5 nested cross-validated predictions were evaluated as a classifier by applying a preset threshold. Given the presented class imbalance, we chose the Matthews correlation coefficient (MCC) as our primary metric of model performance. This process was repeated 10 times, each with a different random seed, to assess robustness.

This analysis showed that only two tumor profiles were predictive (DNA methylation and upper-quartiled mRNA expression) and only in combination with one of the algorithms (random forest). The most predictive model was generated by random forest using the DNA methylation (median MCC of 0.364), which is substantially predictive for this type of problems [8]. We also observed that OMC and hyperparameter tuning do not always provide better models. This was unexpected for OMC, as it has been improved in similar problems involving other drugs and cancer types [6,7]. We hope that new cohorts of temozolomide-treated LGG patients with tumors profiled for DNA methylation will soon become available to further evaluate this model and retrain it with longer datasets.

## References

[1] Sepúlveda-Sánchez JM, Langa JM, Arráez MÁ, Fuster J, Laín AH, Reynés G, González VR, Vicente E, Denis MV, Gallego Ó. SEOM clinical guideline of diagnosis and management of low-grade glioma (2017). Clin Trans Oncol. 2018;20(1):3–15.10.1007/s12094-017-1790-3PMC578560129124520

[2] Field KM, Rosenthal MA, Khasraw M, Sawkins K, Nowak AK. Evolving management of low grade glioma: No consensus amongst treating clinicians. J. Clin. Neurosci. 2016;23:81–87.26601811 10.1016/j.jocn.2015.05.038

[3] Stupp R, Mason WP, van den Bent MJ, Weller M, Fisher B, Taphoorn MJB, Belanger K, Brandes AA, Marosi C, Bogdahn U, et al. Radiotherapy plus concomitant and adjuvant temozolomide for glioblastoma. New Engl J Med. 2005;352(10):987–996.15758009 10.1056/NEJMoa043330

[4] Everhard S, Kaloshi G, Crinière E, Benouaich-Amiel A, Lejeune J, Marie Y, Sanson M, Kujas M, Mokhtari K, Hoang-Xuan K, et al. MGMT methylation: A marker of response to temozolomide in low-grade gliomas. Ann Neurol. 2006;60(6):740–743.17192931 10.1002/ana.21044

[5] Kontogeorgos G, Thodou E. Is MGMT the best marker to predict response of temozolomide in aggressive pituitary tumors? Alternative markers and prospective treatment modalities. Hormones (Athens). 2019;18(4):333–337.31721137 10.1007/s42000-019-00145-1

[6] Bomane A, Gonçalves A, Ballester PJ. Paclitaxel response can be predicted with interpretable multivariate classifiers exploiting DNA-methylation and miRNA data. Front Genet. 2019;10:1041.31708973 10.3389/fgene.2019.01041PMC6823251

[7] Ogunleye AZ, Piyawajanusorn C, Gonçalves A, Ghislat G, Ballester PJ. Interpretable machine learning models to predict the resistance of breast cancer patients to doxorubicin from their microRNA profiles. Adv Sci. 2022;9(24):2201501.10.1002/advs.202201501PMC940364435785523

[8] Dang CC, Peón A, Ballester PJ. Unearthing new genomic markers of drug response by improved measurement of discriminative power. BMC Med Genomics. 2018;11(1):1–14.29409485 10.1186/s12920-018-0336-zPMC5801688

# Abstract: 41. Research Progress on Automated and Intelligent Tools for Bias Risk Assessment in Medical Research

**Authors:** Junxian Zhao, Xiaohui Wang, Yaolong Chen

**Affiliations:** ^1^ School of Public Health, Lanzhou University, Lanzhou, China.; ^2^ Evidence-based Medicine Center, School of Basic Medical Sciences, Lanzhou University, Lanzhou, China.; * Address correspondence to: chevidence@lzu.edu.cn

**Background:** Assessing bias risk in medical research is crucial in the field of medicine. The use of automated and intelligent tools or platforms to accelerate the evaluation of bias risk in medical research has become one of the core issues in medical research. The automation of bias risk evaluation through artificial intelligence technology will alleviate the workload of researchers to a certain extent.

**Objectives:** The purpose of this article is to collect a comprehensive list of automated tools and platforms designed for evaluating bias risk in medical research, providing researchers with options to select from.

**Methods:** The systematic search was conducted through CBM (China Biology Medicine), CNKI (China National Knowledge Infrastructure), Wanfang Data, and Google Scholar to collect automated tools and platforms that currently exist for bias risk evaluation.

**Results:** Our findings indicate that leading automated tools and platforms for evaluating bias risk include RobotReviewer, the automated bias risk assessment model based on linear support vector machine (SVM) classifier, and the automated bias risk assessment model based on BERT (bidirectional encoder representations from transformers).

**Conclusion:** Despite the considerable progress made in developing automated tools and platforms for evaluating bias risk, significant obstacles persist with regard to their practical application. As the use of these automated and intelligent tools and platforms in medical research continues to grow, however, researchers will benefit from increased efficiency and convenience when evaluating bias risk, which is of great significance in promoting scientific clinical decision-making.

# Abstract: 42. Scoping Review of Quality Assessment for Knowledge Graph

**Authors:** Yue Yu, Yu Yang

**Affiliations:** ^1^ School of Economics and Management, University of Science and Technology Beijing, Beijing, China.; ^2^ National Institute of Health Data Science, Peking University, Beijing, China.; * Address correspondence to: yangyu@bjmu.edu.cn

**Background:** Since the concept of knowledge graph was introduced in 2012, it has attracted increasing attention from experts and scholars in various fields. As the establishment and application of knowledge graphs become prevalent, there is a growing interest in evaluating their quality. While scholars have proposed several suggestions for universal knowledge graphs, there is a lack of quality evaluation frameworks that cater to the unique characteristics of domain knowledge graphs, especially the knowledge graph used for medicine since it is particularly critical due to its relation to health and life safety issues, making quality control imperative. However, incomplete, inaccurate, inconsistent, and other quality problems in knowledge graphs can affect the reliability and safety of AI technology applications in medical practice and research (Fig. 25).

**Fig. 25.** The 16 dimensions of knowledge graph quality assessment.

**Objectives:** Our study aimed to conduct a scoping review of research literature on the quality evaluation of knowledge graphs and related technologies. The goal was to sort out and summarize the dimensions, indicators, and evaluation methods of the existing quality assessment and related studies.

**Methods:** We systematically searched for literature published until August 2022 for studies of quality evaluation framework or methodology in knowledge graph and relative technology (e.g., link of data, semantic web, and ontology). The literature sources included the Web of Science, PubMed, IEEE Xplore, and ACM digital library. The researchers screened the literature based on pre-established inclusion and exclusion criteria and extracted information about the related dimensions, metrics, and quality assessment methods. Finally, all the information was summarized, described, and integrated.

**Results:** The study included 17 literature sources, from which 16 dimensions, 82 indicators, and 9 evaluation methods related to the quality evaluation of knowledge graphs were extracted and sorted out. The 16 dimensions of quality evaluation mainly included accuracy, accessibility, completeness, complexity, consistency, interlinking, interoperability, relevancy, security, timeliness, trustworthiness, comprehensibility, adoption, structure, coherence, and uniqueness. Each dimension had different evaluation metrics, such as syntactic and semantic for accuracy evaluation. Regarding evaluation methods, different studies proposed various methods with different focuses. However, the three-stage and six-step method proposed by Anisa Rula was commonly adopted, including requirements analysis and use case analysis in the first stage, quality assessment in the second stage, quality problem identification, fundamental analysis, and advanced analysis in the second step, and quality improvement, root cause analysis, and problem-solving in the third stage.

**Conclusion:** More studies in recent years built frameworks and developed methods for quality evaluation of knowledge graphs following the idea of “Fitness for use.” Moreover, the existing research on quality evaluation mainly focuses on general knowledge graphs, with few studies on medical knowledge graphs. The “Fitness-foruse” is a nebulous concept with few hard and fast rules established thus far. This study systematically reviews the dimensions, indicators, and methods of quality evaluation of knowledge graphs and related technologies using a generalized evaluation method. It could provide valuable references for further exploring and establishing a suitable framework and methods for the quality evaluation of knowledge graphs in healthcare or medical scenarios.

# Abstract: 43. Semantic Extension for Cross-Modal Retrieval of Medical Image-Diagnosis Report

**Authors:** Guohui Ding, Qi Zhang, Lulu Sun, Yuqi Liu, Jiahao Zhang

**Affiliations:** School of Computer, Shenyang Aerospace University, Shenyang, China.; * Address correspondence to: 1716370283@qq.com

In recent years, cross-modal hash retrieval has provided a new perspective for computer-aided diagnostic systems owing to the unprecedented growth of multimodal data in the medical field, and these methods have achieved some academic results. However, existing cross-modal retrieval methods in the field of medicine adopt the simple similarity discrimination strategy based on shared labels and miss information on rich semantic associations from data of different modalities without considering the inherent hierarchy of disease labels in medicine, which will lead to semantic isolation between labels and the loss of query results. To solve this problem, we propose a cross-modal retrieval method called semantic extension for cross-modal retrieval of medical images and diagnostic reports (SECMR) designed to exploit hierarchal semantic associations between labels to constrain a hash code with more semantics. In this case, for some intractable diseases or clinical rare diseases, the method returns similar cases through hierarchical semantic expansion, which can provide some suggestions and diagnostic ideas for doctors, instead of returning no results. In the proposed approach, we first mine the underlying hierarchical associations between labels from large-scale medical corpora using an unsupervised word-embedding model. The underlying associations are then transformed into multilevel semantic matrices as supervised information to learn a common representation of images and texts. Thus, the semantic structure of the original space can be comprehensively preserved in hash code by assigning weights to semantic matrices with different levels. Furthermore, we introduce focal loss to address the common problem of class imbalance in the medical domain. The overall framework of the algorithm is shown in Fig. 26. The results of experiments on the MIMIC-CXR dataset show that our cross-modal retrieval method achieved the best performance compared to other state-of-the-art methods. Thus, by gradually extending query semantics to higher-level concepts, the semantic structure of the original space can be maintained more effectively to support the retrieval of more comprehensive relevant information and improve performance on cross-modal retrieval tasks.

**Fig 26.** The overall framework of the algorithm.

# Abstract: 44. Survival Disparities among Mobility Patterns of Patients with Cancer: A Population-Based Study

**Authors:** Fengyu Wen, Yike Zhang, Chao Yang, Pengfei Li, Qing Wang, Fuzhong Xue, Luxia Zhang

**Affiliations:** ^1^ Institute of Medical Technology, Peking University Health Science Center, Beijing, China.; ^2^ National Institute of Health Data Science at Peking University, Beijing, China.; ^3^ School of Public Health, Cheeloo College of Medicine, Shandong University, Jinan, Shandong, China.; ^4^ National Institute of Health Data Science of China, Shandong University, Jinan, Shandong, China.; ^5^ Renal Division, Department of Medicine, Peking University First Hospital, Peking University Institute of Nephrology, Beijing, China.; ^6^ Research Units of Diagnosis and Treatment of Immune-mediated Kidney Diseases, Chinese Academy of Medical Sciences, Beijing, China.; ^7^ Advanced Institute of Information Technology, Peking University, Hangzhou, China.; * Address correspondence to: zhanglx@bjmu.edu.cn (L.Z.); xuefzh@sdu.edu.cn (F.X.)

**Abstract Competition Winner – Second Prize**

**Background:** Cancer is a major health problem worldwide. With the rapid population aging and the accumulated effects of risk factor exposure, the demand for cancer care in China has been growing. A number of cancer patients choose to travel to hospitals outside their residential cities. However, evidence on the association between patient mobility and outcomes is still limited.

**Objectives:** We aimed to evaluate the association between pattern of patient mobility and survival among patients with cancer.

**Methods:** Data of patients hospitalized for cancer between January 2015 and December 2017 from a medical insurance sampling database of Shandong Province were analyzed. According to the cities of hospitalization and residency, intra-city and two mobility patterns including local center and national center pattern were defined. A sequential matching method was conducted to deal with imbalanced data. Patients belonging to intra-city pattern were matched to patients with two mobility patterns on demographics, marital status, cancer type, comorbidity, and hospitalization frequency, using propensity score matching. Kaplan–Meier method was adopted to estimate 5-year survival, and Cox proportional hazards model was used to estimate the associations between all-cause mortality and patient mobility. Subgroup analysis according to cancer type and healthcare resource was performed.

**Results:** Among 20,602 cancer patients, there were 17,035 (82.7%) patients belonging to intra-city pattern, 2,974 (14.4%) patients belonging to local center pattern, and 593 (2.9%) patients belonging to national center pattern. Significant survival disparities were observed for both comparisons between intra-city and local center patterns [5-year survival rate, 65.4% versus 69.3%; hazard ratio (HR), 0.85; 95% confidence interval (CI), 0.77 to 0.95] and between intra-city and national center patterns (5-year survival rate, 64.5% versus 69.3%; HR, 0.80; 95% CI, 0.67 to 0.97). The disparities in overall 5-year survival were primarily associated with cancer type and marital status. Significant survival disparities existed between intra-city and local center patterns in common cancer and below-average health resource subgroups, and between intra-city and national center patterns in uncommon cancer and above-average health resource subgroups (Fig. 27).

**Fig. 27.** HRs of hospitalization patterns for sequentially matched cancer patients. *P < 0.1, **P < 0.05, ***P < 0.01.

**Conclusion:** We found significant survival disparities among different mobility patterns of patients with cancer. Improving quality of cancer care is crucial, especially for cities with suboptimal healthcare resources.

# Abstract: 45. The Association of Vision Loss, Hearing Loss, and Dual Sensory Loss with the Depression Trajectories in Middle-Aged and Older People in China

**Authors:** Yuchen Liu, Wenwen Liu, Jun Ma, Yangfan Chai, Guilan Kong

**Affiliations:** ^1^ National Institute of Health Data Science, Peking University, Beijing, China.; ^2^ Peking University Third Hospital, Beijing, China.; ^3^ Advanced Institute of Information Technology, Peking University, Hangzhou, Zhejiang, China.; * Address correspondence to: Guilan.kong@hsc.pku.edu.cn; † These authors contributed equally to this work.

**Background:** Vision loss, hearing loss, and depression are health issues that cannot be ignored among the middle-aged and older population [1]. Most relevant studies were cross-sectional and showed that the incidence of depression was higher among people with vision or hearing loss [2,3]. However, few studies were about the dynamic trajectories of depression in the middle-aged and older people in China, and there is a lack of research to explore the association of vision or hearing loss with depression trajectories among this population.

**Objectives:** This study aimed to investigate the trajectories of depression in the middle-aged and older population in China, and to explore the association of visual loss, hearing loss, and dual sensory loss (DSL) with depression trajectories.

**Methods:** Four waves of survey data (2011, 2013, 2015, and 2018) from the China Health and Retirement Longitudinal Study (CHARLS) were used as data source [4]. Participants who were over 45 years old, had no depressive symptoms in 2011, had been surveyed in four waves, and had complete depression data were selected. Vision loss, hearing loss, and DSL were identified through self-reporting. Depressive symptoms were assessed by a 10-item Center for Epidemiologic Studies Depression (CES-D) scale. The latent growth mixed model (LGMM) was used to analyze the trajectories of depression symptoms in the four waves, and multinomial logistic regression was used to explore the association of vision loss, hearing loss, and DSL with depression trajectories.

**Results:** A total of 4,922 participants were included for analysis. Taking into account the simplicity, accuracy, and practical significance of the model, four different depression trajectories were identified by LGMM analysis (Fig. 28). Type 1 (74.9%) patients had low depression risk with a steady CES-D score; type 2 (3.2%) had high depression risk with a continuously increasing CES-D score but the increasing rate gradually slowed down; type 3 (11.3%) was gradually changed from low to high depression risk and the increase of CES-D score was gradually accelerated; type 4 (10.6%) had fluctuations in depression development. After adjusting for age, gender, living area, smoking status, drinking status, family income, body mass index (BMI), hypertension, diabetes, and cardiovascular disease, patients with hearing loss at baseline were more likely to present the second type of depression trajectory [RR = 2.43, 95% confidence interval (CI): 1.46 to 4.06, *P* = 0.001] compared to those without hearing loss. Compared to individuals who had no DSL at baseline, individuals with DSL at baseline were more likely to exhibit a third type of trajectory (RR = 1.58, 95% CI: 1.01 to 2.48, *P* = 0.046). The association of vision loss with the depression trajectories was not statistically significant, and this finding was consistent with the study conducted by Kiely et al. [5] in Australia.

**Fig. 28.** Development trajectories of depression.

**Conclusion:** This study indicates that hearing loss and DSL have associations with the trajectories of depression symptoms in middle-aged and older people in China. It reminds us that for middle-aged and older people with vision and hearing loss, we should pay attention to their psychological status for early and timely intervention. This study is of great significance for the development and improvement of physical and mental health management strategies for middle-aged and older people, but further exploration and analysis are needed about the mechanisms underlying the association between sensory loss and the development of depression.

**Acknowledgements:** This study was supported by grants from Beijing Municipal Science & Technology Commission (grant no. 7212201), Humanities and Social Science Project of Chinese Ministry of Education (grant no. 22YJA630036), and Zhejiang Provincial Natural Science Foundation of China (grant no. LZ22F020014).

## References

[1] Gong R, Hu X, Gong C, Long M, Han R, Zhou L, Wang F, Zheng X. Hearing loss prevalence and risk factors among older adults in China. Int J Audiol. 2018;57(5):354–359.29400111 10.1080/14992027.2017.1423404

[2] Simning A, Fox ML, Barnett SL, Sorensen S, Conwell Y. Depressive and anxiety symptoms in older adults with auditory, vision, and dual sensory impairment. J Aging Health. 2019;31(8):1353–1375.29896982 10.1177/0898264318781123PMC6274614

[3] Blay SL, Andreoli SB, Fillenbaum GG, Gastal FL. Depression morbidity in later life: prevalence and correlates in a developing country. Am J Geriatr Psychiatry. 2007;15(9):790–799.17698602 10.1097/JGP.0b013e3180654179

[4] Zhao Y, Hu Y, Smith JP, Strauss J, Yang G. Cohort profile: The China Health and Retirement Longitudinal Study (CHARLS). Int J Epidemiol. 2014;43(1):61–68.23243115 10.1093/ije/dys203PMC3937970

[5] Kiely KM, Anstey KJ, Luszcz MA. Dual sensory loss and depressive symptoms: The importance of hearing, daily functioning, and activity engagement. Front Hum Neurosci. 2013;7:837.24379769 10.3389/fnhum.2013.00837PMC3864127

# Abstract: 46. The Associations of Dietary Patterns with Risk of Depression and Anxiety: A Prospective Cohort Study

**Authors:** Han Chen, Zhi Cao, Yabing Hou, Hongxi Yang, Xiaohe Wang, Chenjie Xu

**Affiliations:** ^1^ School of Public Health, Hangzhou Normal University, Hangzhou, China.; ^2^ School of Public Health, Zhejiang University School of Medicine, Hangzhou, China.; ^3^ Yanjing Medical College, Capital Medical University, Beijing, China.; ^4^ School of Basic Medical Sciences, Tianjin Medical University, Tianjin, China.; ^5^ Hangzhou International Urbanology Research Center and Center for Urban Governance Studies, Hangzhou, China.; * Address correspondence to: xuchenjie@hznu.edu.cn; † These authors contributed equally to this work.

**Background:** Diet is increasingly recognized as an important risk factor for mental health. However, epidemiologic evidence on diet pattern with incident depression and anxiety is still very limited.

**Objective:** The aim of this study is to investigate the associations of dietary patterns (DPs) characterized by a set of nutrients of interest with risk of incident depression and anxiety.

**Methods:** A total of 126,819 participants in the UK Biobank who completed at least two dietary questionnaires were included in the analyses. Dietary data were obtained through 24-h online dietary assessment between 2011 and 2012. Reduced rank regression was applied to derive DPs explaining variability in energy density, free sugars, saturated fat, and fiber intakes. Incidence of depression and anxiety was identified by the Patient Health Questionnaire-9 and General Anxiety Disorder-7 between 2016 and 2017, respectively. Logistic regression models were performed to investigate the associations between DPs and risk of depression and anxiety (Fig. 29).

**Results:** During the follow-up, 2,968 and 2,303 participants developed depression and anxiety, respectively. We identified three main DPs, which explained a total of 74% variation in the above four nutrients. DP1 was characterized by high intakes of chocolate and confectionery, butter, and other animal-fat spreads, and low intakes of vegetables and fresh fruit. DP1 was associated with risk of depression [Q5 versus Q1: odds ratio (OR) = 1.18, 95% confidence interval (CI): 1.05 to 1.33] and anxiety (Q5 versus Q1: OR = 1.16, 95% CI: 1.02 to 1.33). DP2 was characterized by high intakes of sugar-sweetened beverages and other sugary drinks, table sugars, and preserves, and low intakes of butter and other animal fat spreads, and high-fat cheese, but suggested null associations with depression or anxiety. DP3 was characterized by high intakes of butter and other animal fat spreads, milk-based desserts, and low intakes of alcoholic drinks (wine, beer, spirits) and low-fiber bread. DP3 was significantly associated with higher risk of depression and anxiety (Q5 versus Q1: OR = 1.20, 95% CI: 1.06 to 1.35; OR = 1.22, 95% CI: 1.07 to 1.40, respectively).

**Conclusion:** A DP characterized by high intakes of chocolate and confectionery, butter, high-fat cheese, and added sugars, along with low intakes of fresh fruit and vegetables, is associated with a higher risk of incident depression and anxiety. These findings suggest the importance of promoting healthy DPs rich in fruits, vegetables, and whole grains, and low in saturated fats, added sugars, and processed foods for the prevention of depression and anxiety. Interventions aimed at improving dietary quality may represent a novel approach to reducing the risk of these common mental disorders.

**Author contributions:** H.C., Z.C., and C.X. conceived and designed the study. H.C. performed statistical analyses. H.C. and Z.C. drafted the manuscript. C.X. and X.W. supervised the study. All authors aided in the acquisition and interpretation of data and critical revision of the manuscript. C.X. had access to and verified all of the data in the study.

# Abstract: 47. The Influence of Family Cognitive Environment on Early Childhood Language Development: A Retrospective Case–Control Study in Shanghai, China

**Authors:** Zhichao Guo, Dan Cui, Jiajun Bao, Kang Wei, Wenya Yu

**Affiliations:** ^1^ Department of Prevention and Health Care, Yuepu Town Community Health Service Center of Baoshan District, Shanghai 200941, China.; ^2^ School of Public Health, Shanghai Jiao Tong University School of Medicine, Shanghai 200025, China.; * Address correspondence to: jsjyyuwenya@sina.cn

**Background:** Early childhood language development (ECLD) has a high incidence rate of delay and a low early screening rate, and negatively influences the development of other skills of early childhood development (ECD), which has become a serious challenge to ECD. Family cognitive environment is almost whole learning and living environment for children aged 0 to 3 years, whose most communications take place in their families. Therefore, family cognitive environment is a key factor of ECLD, and having a deep understanding of its influencing mechanisms can provide significant evidence for early screening of ECLD and early precise interventions. However, studies on ECLD were very limited currently, especially in developing countries, and there was little research on how family cognitive environment influences ECLD.

**Objectives:** The purpose of this study is to explore the influence of family cognitive environment on ECLD. It is expected to promote ECLD and ECD from perspectives of influencing factors and related influencing mechanisms of ECLD delay.

**Methods:** A retrospective case–control study was conducted. The case group included data about 172 children screened as abnormal ECLD by using Shanghai Child Development Screening Scale II in primary child healthcare department of Shanghai from January 2018 to December 2020. The control group included 516 children with normal ECLD based on a 1:3 pairing ratio and matched with cases by age. Information about birth characteristics, parents’ demographic characteristics, maternal pregnancy and delivery characteristics, and family cognitive environment characteristics were collected for both groups. Family cognitive environment was evaluated from four aspects, including warmth, social adaptation, language environment, and neglect environment, and the total score of the four dimensions was divided into good (scores greater than the 80th percentile), intermediate (scores between the 20th and 80th percentile), and poor level (scores at or below the 20th percentile). Descriptive analysis, *t* test or chi-square test, and logistic regression analysis were used for data analysis.

**Results:** The overall abnormal prevalence of ECLD among children in Shanghai during the 3-year follow-up was 3.99%. Children aged 2 years had the highest abnormal prevalence (2.14%) and accounted for 53.49% of all children with abnormal ECLD. The ratio of poor family cognitive environment in case group reached to 23.84%. All dimensions including warmth, social adaptation, language environment, and neglect environment, and the total score of family cognitive environment in the case group were poorer than those in the control group (*P* < 0.05). Premature birth, mother,s lower educational level, and poor family cognitive environment were risk factors of abnormal ECLD. The possibility of normal ECLD of children with premature birth was 0.692 of full-term children; this probability of children whose mothers were educated below high school was 0.616 of those with mothers being educated at least high school; and this possibility of children with poor family cognitive environment was 0.542 of those with good family cognitive environment (Table 1).

**Table 1.** Comparison on family cognitive environment characteristics between the case and control group

Dimension	Evaluation	Case group (*n* = 172)	Control group (*n* = 516)	*P* value
Total				<0.001
	Good	53 (30.81)	226 (43.80)	
	Intermediate	78 (45.35)	282 (54.65)	
	Poor	41 (23.84)	8 (1.55)	
Warmth				<0.001
	Good	55 (31.98)	226 (43.8)	
	Intermediate	87 (50.58)	287 (55.62)	
	Poor	30 (17.44)	3 (0.58)	
Social adaptation				<0.001
	Good	56 (32.56)	221 (42.83)	
	Intermediate	81 (47.09)	290 (56.2)	
	Poor	35 (20.35)	5 (0.97)	
Languageenvironment				<0.001
	Good	60 (34.88)	222 (43.02)	
	Intermediate	78 (45.35)	281 (54.46)	
	Poor	34 (19.77)	13 (2.52)	
Neglect environment				0.003
	Good	62 (36.05)	230 (44.57)	
	Intermediate	91 (52.91)	276 (53.49)	
	Poor	19 (11.05)	10 (1.94)	

**Conclusion:** The highest prevalence of ECLD delay was at age 2 years, and precise interventions was the key for decreasing the abnormal ECLD rate before age 2. Strategies of optimizing family rearing environment, family learning environment, and parent–child interactions for improving family cognitive environment was significant to promote ECLD. It encouraged to provide interventions and guidance of parent–child activities and communications for children with premature birth and mothers with lower educational level as early as possible to decrease ECLD delay.

# Abstract: 48. Trajectory Analysis of Physiological Signals for Patients with Intraoperative Anaphylaxis Using Real-World Time Series Data

**Authors:** Haoran Su, Bailin Jiang, Guilan Kong, Yi Feng

**Affiliations:** ^1^ National Institute of Health Data Science, Peking University, Beijing, China.; ^2^ Beijing Jishuitan Hospital, Beijing, China.; ^3^ Peking University People’s Hospital, Beijing, China.; ^4^ Advanced Institute of Information Technology, Peking University, Hangzhou, Zhejiang, China.; * Address correspondence to: guilan.kong@hsc.pku.edu.cn (G.K.); fengyi@pkuoh.edu.cn (Y.F.); † These authors equally contributed to this work.

**Background:** Intraoperative anaphylaxis is a rare and life-threatening acute event [1]. A presumed clinical diagnosis can be made at the time of presentation, and it depends on whether there are suspected allergen infusion and clinical features (e.g., hypotension, rash, and erythema) [2]. However, anesthesiologists may not recognize the onset of anaphylaxis until the rapidly progressing symptoms emerge. The delayed detection of anaphylaxis exacerbates the near-fatal events and compromises the effect of intervention [3]. In practice, multiple intraoperative physiological signals are usually monitored in real time. Scrutinizing these signals may be conducive of the timely recognition of anaphylaxis. However, the value of time series data (TSD) and the clinical significance they represent have not yet been explored.

**Objectives:** This study aimed to analyze the trajectory characteristics of physiological signals in patients undergoing intraoperative anaphylaxis using the real-world TSD and to develop a strategy for detecting anaphylaxis before significant hemodynamic disturbances happen.

**Methods:** The data of patients who have undergone surgeries at Peking University People,s Hospital between 2011 January 1 and 2023 January 1 were used for analysis. The inclusion criteria were as follows: (a) having intraoperative hypotension [defined as invasive systolic blood pressure (ISBP) or non-invasive systolic blood pressure (NSBP) <80 mmHg]; (b) with suspected allergen infusion before hypotension; (c) BP returned to normal after discontinuation of suspected allergen infusion if it was ongoing (e.g., plasma and antibiotic) and epinephrine administration. Suspected allergens were identified by an experienced expert. Patients who developed hypotension before the infusion of suspected allergens were excluded. The time point of intraoperative anaphylaxis occurrence was defined as the time when ISBP fell below 80 mmHg and failed to recover for at least 5 min, and NSBP was used as a substitute if ISBP was not recorded; if epinephrine was administrated before ISBP hit the criterion, the time point of epinephrine administration was considered as intraoperative anaphylaxis occurrence time. TSD of physiological signals including ISBP, invasive diastolic blood pressure (IDBP), and heart rate (HR) within the last 5 min before anaphylaxis occurrence was used for analysis. If anaphylaxis occurred in less than 5 min after suspected allergen infusion, we selected the TSD recorded during the period from suspected allergen infusion to anaphylaxis occurrence. Trajectories were fitted by locally weighted regression method.

**Results:** A total of 84 patients with intraoperative anaphylaxis were included in the final analysis. There were 58 patients having completely recorded ISBP and IDBP data in the study period. As shown in Fig. 30, 5-min signal trajectory analysis showed that ISBP and IDBP were steady from the last 300 s to 100 s and then a decrease occurred at around 100 s to the anaphylaxis occurrence, at an average decreasing rate of 2.11 mmHg/10 s and 1.09 mmHg/10 s, and the average proportion of decrease reached 17.97% and 17.85%, respectively.

**Fig. 30.** Physiological signal trajectories and spaghetti plots.

**Conclusions:** In the studied dataset, at around 100 s to the intraoperative anaphylaxis occurrence, patients’ ISBP and IDBP began to show a downward trend, at an average decreasing rate of 2.11 mmHg/10 s and 1.09 mmHg/10 s, respectively. In practice, as long as the BP signals showed the above trajectory characteristics after suspected allergen infusion, there was a strong probability that the intraoperative anaphylaxis may occur. This suggests that clinicians could identify and take interventions in advance for this serious disease by close monitoring of BP trajectory.

**Acknowledgments:** This study was supported by grants from Zhejiang Provincial Natural Science Foundation of China (grant no. LZ22F020014), Beijing Municipal Science & Technology Commission (grant no. 7212201), and Humanities and Social Science Project of Chinese Ministry of Education (grant no. 22YJA630036).

## References

[1] Volcheck GW, Hepner DL. Identification and management of perioperative anaphylaxis. J Allergy Clin Immunol Pract. 2019;7(7):2134–2142.31154032 10.1016/j.jaip.2019.05.033

[2] Manian DV, Volcheck GW. Perioperative anaphylaxis: Evaluation and management. Clin Rev Allergy Immunol. 2022;62(3):383–399.34247332 10.1007/s12016-021-08874-1

[3] Muraro A, Worm M, Alviani C, Cardona V, DunnGalvin A, Garvey LH, Riggioni C, de Silva D, Angier E, Arasi S, et al. EAACI guidelines: Anaphylaxis (2021 update). Allergy. 2022;77(2):357–377.34343358 10.1111/all.15032
